# A Genome-Wide Association Study of Wheat Spike Related Traits in China

**DOI:** 10.3389/fpls.2018.01584

**Published:** 2018-10-31

**Authors:** Jing Liu, Zhibin Xu, Xiaoli Fan, Qiang Zhou, Jun Cao, Fang Wang, Guangsi Ji, Li Yang, Bo Feng, Tao Wang

**Affiliations:** Chengdu Institute of Biology, Chinese Academy of Sciences, Chengdu, China

**Keywords:** wheat, spike length, kernels per spike, spikelet number, artificial selection, GWAS, haplotype, KASP

## Abstract

Rapid detection of allelic variation and identification of advantage haplotypes responsible for spike related traits play a crucial role in wheat yield improvement. The released genome sequence of hexaploid wheat (Chinese Spring) provides an extraordinary opportunity for rapid detection of natural variation and promotes breeding application. Here, selection signals detection and genome-wide association study (GWAS) were conducted for spike related traits. Based on the genotyping results by 90K SNP chip, 192 common wheat samples from southwest China were analyzed. One hundred and forty-six selective windows and one hundred and eighty-four significant SNPs (51 for spike length, 28 for kernels per spike, 39 for spikelet number, 30 for thousand kernel weight, and 36 for spike number per plant) were detected. Furthermore, tightly linkage and environmental stability window clusters and SNP clusters were also obtained. As a result, four SNP clusters associated with spike length were detected on chromosome 2A, 2B, 2D, and 6A. Two SNP clusters correlated to kernels per spike were detected on 2A and 2B. One pleiotropy SNP cluster correlated to spikelet number and kernels per spike was detected on 7B. According to the genome sequence, these SNP clusters and their overlapped/flanking QTLs which have been reported previously were integrated to a physical map. The candidate genes responsible for spike length, kernels per spike and spikelet number were predicted. Based on the genotypes of cultivars in south China, two advantage haplotypes associated with spike length and one advantage haplotype associated with kernels per spike/spikelet number were detected which have not been effectively transited into cultivars. According to these haplotypes, KASP markers were developed and diagnosed across landraces and cultivars which were selected from south and north China. Consequently, KASP assay, consistent with the GWAS results, provides reliable haplotypes for MAS in wheat yield improvement.

## Introduction

Bread wheat (*Triticum aestivum* L.) is the most widely grown food crop and provides the main energy requirements for about one third of the global people (Guo et al., 2018). As the world population growing continuously, yield improvement is an on-going task for wheat breeding. Three key components, spike number, kernels per spike (KPS), and thousand kernel weight (TKW), collectively determine the wheat yield. Furthermore, spike length (SL) and spikelet number (SN) which affect KPS and spike number per plant (SNPP) also play important role in improving wheat yield (Guo et al., 2017; Liu K. et al., 2018). Therefore, discovering the crucial SNPs/quantitative trait loci (QTLs) associated with spike related traits is the urgent task for further wheat breeding program.

Conventional wheat breeding (artificial selection) is mainly based on phenotypic selection which is one of the most important steps for genetic improvement. However, it is blindness, empirical, inefficiency and costs a long time. Fortunately, the hardworking and intelligence of the breeders would have kept signatures in the wheat genome during crop improvement, and this selection signal could be detected using different methods (Gao et al., 2017). Identification of selection signal has been reported in many plants, such as soybean (Zhou et al., 2015; Wang J. et al., 2016), rice (Xu et al., 2012), peach (Cao et al., 2016), tomato (Lin et al., 2014), and wheat (Cao et al., 2016). In consideration of selection signal could not associated with phenotype, GWAS would be conducted to annotate the signatures in depth (Zhou et al., 2015). Based on the linkage disequilibrium (LD), GWAS is effective method to explore complex quantitative trait loci and allelic variation for a particular trait. SNPs with more high association scores were more likely to be close to the candidate genes, which mean the possibility by using GWAS to detect candidate genes (Brachi et al., 2011). GWAS has been widely used in crops to predict phenotypic related candidate genes (Si et al., 2016; Wang X. et al., 2016; Liu J. et al., 2018).

Compared to the rice and *Arabidopsis*, geneticist and breeders have been looking forward to the wheat genome sequence for 14 years (Kaul et al., 2000; Goff et al., 2002). The annotated reference wheat genome, IWGSC RefSeq v1.0, released on the IWGSC website (http://www.wheatgenome.org/) opened a new avenue in exploring genome sequences, isolating novel genes and rapidly detecting natural variations. Consideration of the forward genetics, IWGSC RefSeq v1.0 with precise scaffold ordering and annotation, integrated assembly and complete gene models, could fully resolve problems (Appels et al., 2018). Take GWAS for example, candidate genes can be easily obtained around the leading SNP based on the LD decay. According to the annotation of the wheat reference genome, candidate genes could be further screened. Then, the function of targeted genes can be confirmed by gene editing, and the advantage genotype could be used for selection breeding directly (Appels et al., 2018).

Genes and QTLs associated with spike related traits spread all over the 21 chromosomes of wheat (Liu K. et al., 2018). The *Q* gene located on chromosome 5A confers a free threshing spike and pleiotropically influences many other domestication-related traits such as spike length, plant height, and spike emergence time (Simons et al., 2006). The *TaSnRK2* gene encoded sucrose non-fermenting 1-related protein kinase was detected on chromosome 4A/4B/4D. It plays crucial roles in response to various environment stimuli and shows significant correlation to spike length and thousand kernel weight (Miao et al., 2017; Zhang et al., 2017). *AGO1d* gene mutant in a tetraploid durum wheat produced shorter spikes and fewer kernels per spike than wild-type controls (Feng et al., 2017). The compactum (*C*) gene locates on the long arm of chromosome 2D near the centromere and affects spike compactness, grain size, grain shape and grain number per spike (Johnson et al., 2008). *TaCKX6-D1* was found to be significantly associated with TKW and KPS by controlling cytokinin levels (Zhang L. et al., 2012). Also, *TaSAP1* was confirmed to be associated with TKW and KPS by involving in response to stresses (Chang et al., 2013). *Ppd-1* on 2D was identified as an inhibitor of paired spikelet formation by regulating the expression of *FT* gene, consequently decreased the number of spikelet (Boden et al., 2015). *TaMOCI-7A* and *TaTEF-7A* also have been found to be stably associated with spikelet number per spike (Zheng et al., 2014; Zhang et al., 2015). Tiller inhibition gene (*tin*) mapped on the short arm of chromosome 1A and productive tiller number gene (PTN) were notedly associated with spike number per plant (Spielmeyer and Richards, 2004; Naruoka et al., 2011). The classical grain weight related genes, such as *TaGW2-A1, TaTGW6-A1, TaCwi, TaGS5-A1, TaGS-D1, TaSus1* and *TaSus2* were located on 6A, 3A, 2A/4A/5D, 3A, 7D, and homoeolouous groups 7 and 2 (Jiang et al., 2011, 2015; Su et al., 2011; Hou et al., 2014; Rasheed et al., 2014; Zhang et al., 2014; Wang et al., 2015; Hanif et al., 2016; Zhai et al., 2018). In addition, a lot of QTLs associated with spike-related traits (Huang et al., 2006; Naruoka et al., 2011; Cui et al., 2014, 2017; Azadi et al., 2015; Fan et al., 2015; Gao et al., 2015; Li et al., 2016; Luo et al., 2016; Zhai et al., 2016; Guo et al., 2017; Liu et al., 2017; Lozada et al., 2017; Mwadzingeni et al., 2017; Ogbonnaya et al., 2017; Schulthess et al., 2017; Shi et al., 2017; Sun et al., 2017; Xu et al., 2017; Zhou et al., 2017), have been reported in previous studies. However, few genes or QTLs associated with spike related traits has been used for wheat breeding.

In this study, 192 wheat lines were genotyped by using the 90K Illumina iSelect SNP Array (Wang et al., 2014). Based on multi-environmental trial data, GWAS were conducted to identify favorable SNP clusters for yield-related traits, such as spike length, spikelet number, kernels per spike, thousand kernel weight and spike number per spike. SNPs overlapping these haplotypes were used to develop KASP (Kompetitive Allele Specific PCR) markers, which were subsequently validated on a large sample panel. These KASP markers could ultimately used in genome selection for wheat yield improvement.

## Materials and methods

### Plant material and phenotype analysis

The natural population used for GWAS including 25 synthetic hexaploid wheat lines, 80 landraces and 87 cultivars (Supplementary Table 1). They were planted in four environments: 2014–2015 in Shuangliu (E4); 2015–2016 in Shuangliu (E3); 2015–2016 Shifang with high nitrogen treatment (E2); 2015–2016 Shifang with low nitrogen treatment (E1). In the high nitrogen field, 60 kg N ha^−1^ were applied after sowing. In the low nitrogen field, no nitrogen was applied during the whole growing period. The natural population used for KASP assay including 135 landraces and 141 cultivars were planted in the 2017–2018 growing seasons in Shuangliu (Supplementary Table 2). Wheat lines were planted in a randomized complete block with three replications per location. Every block had two rows, with a single row of 1.2 m long and 0.2 m apart. Twelve seeds were hand-planted in each row. Crop management followed local agricultural practice. After sowing, approximately 40 kg N ha^−1^ were applied except the high and low nitrogen fields. Fungicide was applied at seedling stage and heading period to control diseases and pests, but no irrigation was used.

Main spikes of six plants were randomly selected for phenotype analysis. Spike length was measured from the base of the rachis to the topmost spikelet, excluding the awns. Spikelet number was counted from the basal sterile spikelet to the top fertile spikelet. Kernels per spike were estimated by hand-threshing the maturity spike. Thousand kernel weight was measured by SC-E software (Handzhou Wanshen Detection Technology Co., Ltd., Hangzhou, China) from weighting more than 200 random kernels with two technical repeats. Spike number per plant was counted the spike with more than five kernels in one plant. Statistical data were analyzed using SPSS 19.0 software (https://www.ibm.com/analytics/cn/zh/technology/spss/). Outliers were deleted before analysis. Analysis of variance (ANOVA) was performed to test the differences caused by the influence of genotype and environment for each spike trait. Pearson's correlation analyses were conducted to pinpoint the relationships among the spike related traits.

### SNP calling and LD estimation

Genomic DNA was extracted from the fresh leaves of seedling wheat using the modified cetyl trimethyl ammonium bromide (CTAB) method (Murray and Thompson, 1980). At least 5 μg genomic DNA of each line was used for genotyping by the wheat 90K Illumina iSelect SNP Array at the Compass Biotechnology Co., Ltd. After quality control (filter criteria: sample call rate > 0.8, MAF > 0.05, SNP call rate > 0.9, HWE < 0.000001), 13,154 polymorphic SNPs were selected for follow-up analysis.

Based on the pruned data, linkage disequilibrium (LD) was calculated using PLINK60 (Version 1.90) software and the LD decay graphs were plotted using an R script. The pairwise *r*^2^ (squared allele frequency correlation) values were calculated using SNPs within 200 Mb for cultivars, landraces and all samples, respectively. The distance that the LD decays to half of its maximum value was estimated.

### Detection of artificial selection signals

The genetic differentiation (*F*_ST_) and reduction of nucleotide diversity (*ROD* = 1–π_*cultivar*_/π_*landrance*_) were calculated for non-overlapping 100-kb sliding windows across the genome using VCFtools v0.1.14 (https://github.com/vcftools/vcftools). The windows in the upper 99% of the pool's empirical distribution for both *F*_ST_ statistics and *ROD* values were selected as candidate regions. These genes, located in the windows detected by two methods or existed in the window clusters according to the distance less than 1 Mb based on the LD, were chosen as candidate genes. The annotation information of these candidate genes were extracted from the IWGSC website (http://www.wheatgenome.org/). Meanwhile, *Arabidopsis* (https://www.arabidopsis.org/) and rice (http://rapdb.dna.affrc.go.jp/) functional gene databases were used for annotation.

### Genome-wide association study

GWAS for spike related traits were performed in 192 wheat lines using the compressed mixed linear model (CMLM) by the GAPIT package, which took the results of population stratification and kinship as covariate to minimize false positives (Lipka et al., 2012). A threshold *P*-value of 0.001 (–log_10_*P* = 3) was used to declare significant SNPs for GWAS results. To uncover the candidate clusters, stable SNPs (based on LD decay) existed in more than two environments underlying association signals were selected.

### An integrated map based on the wheat genome sequence

The stable SNP clusters were mapped on the wheat genome. Together with previous report QTLs, regions of interest (spike length, spikelet number, kernels per spike) were positioned onto the newly released reference genome sequence of Chinese Spring by blasting their flanking or peaking marker sequences against the IWGSC RefSeq v1.0 (https://urgi.versailles.inra.fr/blast_iwgsc/blast.php). MapChart Ver. 2.3 was used for map drawing (https://www.wageningenur.nl/en/show/Mapchart.htm). (Voorrips, 2002)

### KASP assay

Based on LD decay, KASP markers were developed by using the SNPs overlapping these candidate haplotypes. A total of 276 wheat lines were genotyped, including 146 landraces (60 southern lines and 86 northern lines) and 130 cultivars (67 southern lines and 73 northern lines), collected from diverse wheat zoning in China (Supplementary Table 2). In the southern wheat lines, 39 landraces and 49 cultivars have been used for GWAS. Four pairs of KASP markers were developed for detection three haplotypes (Table 1). The KASP assay was carried out according to the manufacturer's recommendation (LGC Genomics, Beverly, MA, USA) and the reference Patterson et al. (2017). Amplification was carried out starting with 15 min at 94°C, followed by 10 touchdown cycles of 20 s at 94°C and 60 s at 65–57°C, and 26–35 cycles of 94°C for 20 s and 60°C for 1 min. End point genotyping was done using the CFX Manager 3.1 software. The specificity and sensitivity of all tested markers were listed on Supplementary Table 3.

**Table 1:** Candidate SNP clusters associated with spike related traits detected in this study.

**Trait**	**SNP**	**SNP ID**	**Chr**.	**Pos. (Mb)**	**-Log_10_*P***	**Alleles**	**Effect alleles**	**MAF**	***R*^2^**	**Environment**
SL	BobWhite_c17476_149	IWB1047	2A	77.94	3.80	T/C	T	0.204	0.068	E1
	Kukri_rep_c71876_521	IWB50153	2A	78.33	3.03	A/G	A	0.194	0.052	E1
	wsnp_Ex_c15325_23565935	IWA2007	2A	78.33	3.76	A/G	G	0.202	0.067	E1
	Kukri_c7818_2581	IWB47747	2A	78.34	3.80	T/C	T	0.204	0.068	E1
	wsnp_Ex_c15325_23564654	IWA2005	2A	78.34	3.80	A/G	A	0.204	0.068	E1
SL	CAP7_c3814_303	IWB14055	2B	233.27	3.09/3.42	T/C	C	0.304/0.309	0.051/0.061	E3/E4
	BS00066546_51	IWB9834	2B	233.27	3.09/3.42	T/G	T	0.304/0.309	0.051/0.061	E3/E4
	CAP11_c2947_204	IWB12818	2B	233.27	3.13/3/38	T/G	T	0.301/0.306	0.052/0.060	E3/E4
	*kSL-2B	IWB12818-A010074	2B	233.27		A/C	A		
	Tdurum_contig52063_1077	IWB72239	2B	233.27	3.09/3.42	A/C	A	0.304/0.309	0.051/0.061	E3/E4
	Excalibur_c58566_284	IWB27823	2B	233.55	3.25	T/C	T	0.324	0.057	E4
SL	Excalibur_c44325_638	IWB26541	2D	608.20	3.10	T/C	T	0.220	0.051	E3
	RAC875_c26979_266	IWB56014	2D	608.57	3.76/3.45	T/C	C	0.212/0.210	0.064/0.061	E3/E4
	RAC875_c14766_1063	IWB53992	2D	608.77	3.87	A/G	G	0.107	0.067	E3
	tplb0021a02_817	IWB74078	2D	608.92	3.16	T/C	C	0.209	0.052	E3
SL	RAC875_c49875_405	IWB58592	6A	60.17	3.91	A/G	G	0.161	0.071	E2
	RAC875_c60695_112	IWB59541	6A	60.41	3.53/3.38/3.92/4.98	A/G	G	0.191/0.192/0.186/0.184	0.063/0.059/0.068/0.095	E1/E2/E3/E4
	*kSL-6A.1	IWB68523-A010085	6A	60.74		A/C	C		
	BS00079942_51	IWB11102	6A	61.02	3.26	A/G	A	0.137	0.057	E2
	wsnp_CAP7_c1839_908093	IWA1050	6A	61.21	3.42	A/G	G	0.153	0.060	E2
	wsnp_CAP7_c1839_908011	IWA1049	6A	61.21	3.42	A/G	G	0.153	0.060	E2
	wsnp_CAP7_c1839_907899	IWA1048	6A	61.21	3.42	T/C	C	0.153	0.060	E2
	kSL-6A.2	IWB42599-A010081	6A	61.39		A/G	G		
	Kukri_c45898_147	IWB45584	6A	61.39	3.42	T/C	C	0.153	0.060	E2
KPS	Excalibur_c6660_746	IWB28454	2A	691.22	3.19	T/C	C	0.223	0.046	E4
	wsnp_Ku_c8927_15048149	IWA7339	2A	691.22	3.21	A/C	C	0.214	0.047	E4
	Kukri_c7193_3176	IWB47525	2A	691.22	3.44	A/G	G	0.225	0.051	E4
	Kukri_c7193_5892	IWB47527	2A	691.23	3.73	A/G	G	0.253	0.056	E4
KPS	Excalibur_c74622_265	IWB28725	2B	775.83	4.35	T/G	T	0.069	0.058	E1
	Kukri_c16161_231	IWB41532	2B	776.70	4.40	T/C	T	0.128	0.059	E1
	Excalibur_c40335_454	IWB26132	2B	776.75	4.96	T/C	T	0.125	0.068	E1
	Kukri_c26697_366	IWB43329	2B	776.98	3.11	T/C	C	0.186	0.039	E1
	Ra_c1597_2503	IWB43329	2B	776.98	4.76	A/G	G	0.117	0.065	E1
	Excalibur_c16329_370	IWB22772	2B	777.33	4.96	T/C	T	0.125	0.068	E1
	Ex_c27573_778	IWB20376	2B	777.51	4.08	T/C	T	0.125	0.054	E1
KPS (SN)	Excalibur_c36221_1031	IWB25689	7B	716.59	4.18(4.00/3.61)	T/C	T	0.184(0.181/0.183)	0.056(0.054/0.057)	E1(E1/E4)
	IACX5767	IWB36018	7B	716.96	3.63(3.81/3.67)	A/G	G	0.207(0.204/0.208)	0.047(0.051/0.058)	E1(E1/E4)
	BS00003630_51	IWB5853	7B	716.96	3.63(3.81/3.67)	T/C	C	0.207(0.204/0.208)	0.047(0.051/0.058)	E1(E1/E4)
	BS00009879_51	IWB6129	7B	716.97	3.45(3.76/3.18)	A/G	A	0.186(0.183/0.186)	0.044(0.050/0.049)	E1(E1/E4)
	BS00089322_51	IWB11608	7B	716.97	3.61(3.92/3.23)	T/G	T	0.184(0.181/0.183)	0.047(0.053/0.050)	E1(E1/E4)
	*kKPS/SN-7B	IWB10891-A010071	7B	717.20		A/G	G		

*SNP loci used for KASP assay.

## Results

### Phenotypic assessment

One hundred and ninety-two bread wheat lines including synthetic hexaploid, landraces and cultivars were tested in the present study. The phenotypic performance of the investigated traits for the wheat natural population in four environments was shown in Supplementary Figure 1, Supplementary Table 4. The coefficients of variation for these traits in each environment ranged from 10.63 to 41.07%, indicating broad phenotypic variation and a large improvement potential. Significant differences were detected among environments for these traits by ANOVA. (Supplementary Tables 4, 5). The variation of SL, SN KPS and SNPP was prominently impacted by environments, which explained 19.35, 6.96, 7.18, and 32.18% of the phenotypic variation, respectively (Supplementary Table 5). Significant Person's correlation coefficients were found among these traits in entire wheat lines (All) and different groups (SH, L, and C) (*P* < 0.05, Supplementary Table 6). A significant negative correlation was observed between TKW and SNPP in the entire wheat lines. However, TKW and SL were found significant positive correlated in the entire wheat lines. Interestingly, the significant negative correlations were observed between TKW and SN, TKW and KPS in the entire wheat lines, but significant positive correlations were found in cultivars. Here, the selective pressure may play an important role for the change of correlation from landraces to cultivars (Zhang D. et al., 2012). Significant positive correlation was detected between SN and KPS in all the groups. However, significant positive correlations were only observed between SL and SN, SL and KPS in cultivars.

### Artificial selection signals during wheat improvement

Wheat lines used in this study include synthetic hexaploid, landraces and cultivars. No obvious population structure among these samples was detected which has been proved by the previous study (Liu K. et al., 2018). Linkage disequilibrium (LD) analysis was performed in cultivars, landraces and all samples, respectively. Compared to a higher LD dropped to half of its maximum value of cultivars (1,053 kb), the value of landraces was lower (785 kb) (Supplementary Figure 2). The higher LD value in cultivated wheat is consistent with the fact that the effect of artificial selection exists in this population.

**Figure 1:**
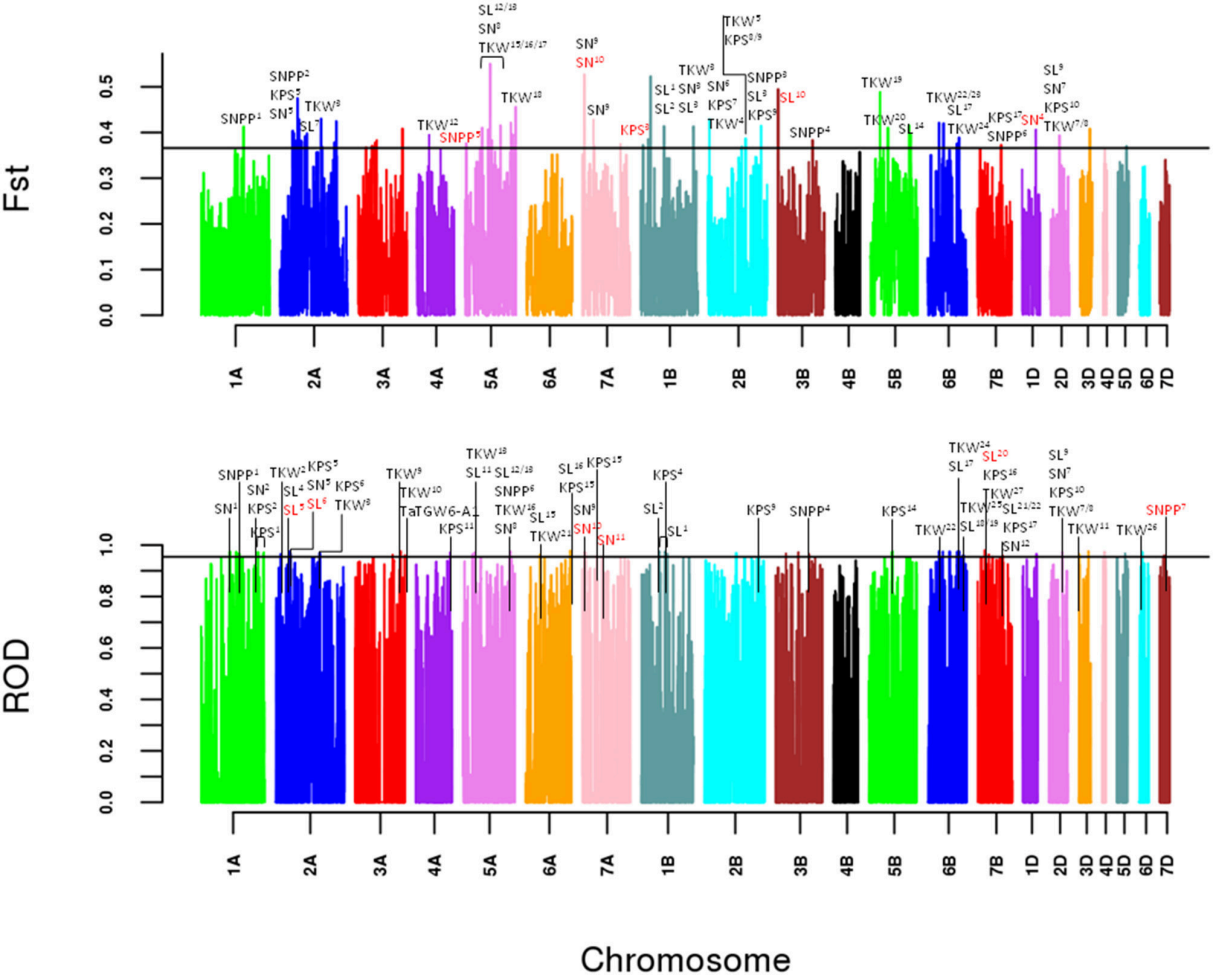
Artificial selection signals detection between landraces and cultivars. Genome-wide scan in a 100 kb non-overlapping sliding window of *F*_ST_ and *ROD*. The lines represent the 99% tails for the empirical distribution of *F*_ST_ and *ROD* statistics, respectively. Selection signals that overlap the currently and previously reported spike related QTLs or markers (Supplementary Table 8) are labeled above.

To identify potential artificial selection signals at the genomic level, genetic differentiation (*F*_ST_) and polymorphism levels (*ROD*) between cultivars and landraces were calculated (Figure 1). The results show that there were 75 and 71 non-overlapping windows detected to be potential selective sweep regions, respectively (Supplementary Table 7). These selective windows accounted for only 0.8% of the whole wheat genome and were not covered all the 21 chromosomes. Fortunately, 10 windows were detected by the two methods. In addition, 19 window clusters were obtained according to the distance less than 1 Mb based on the LD which might be genetic linkage regions to some loci affecting important agronomic traits.

In our analysis, 146 putative selection sweeps were compared with previously reported spike related QTLs or markers based on the LD decay. As a result, 81 selective sweeps were located within the known spike related QTLs or markers (Figure 1, Supplementary Table 8). Primarily, 26 TKW related QTLs and one gene were found in the overlapped regions of 37 selective sweeps. Then, 35, 23, and 22 selective windows overlapped with 18 SL related QTLs, 15 KPS related QTLs and 9 SN related QTLs, respectively. Only 8 sweeps overlapped with 5 SNPP related QTLs. Notably, 2 window clusters on chromosome 2A and 6B were also discovered by the two methods. The window cluster on 2A overlapped with the previously reported *Rht* gene *TaUBP24* (Liu J. et al., 2018). Meanwhile, the window cluster on 6B located in the confidence interval of previously reported QTLs for thousand kernel weight and spike length (Wang et al., 2011; Mir et al., 2012).

### GWAS

**Figure 2:**
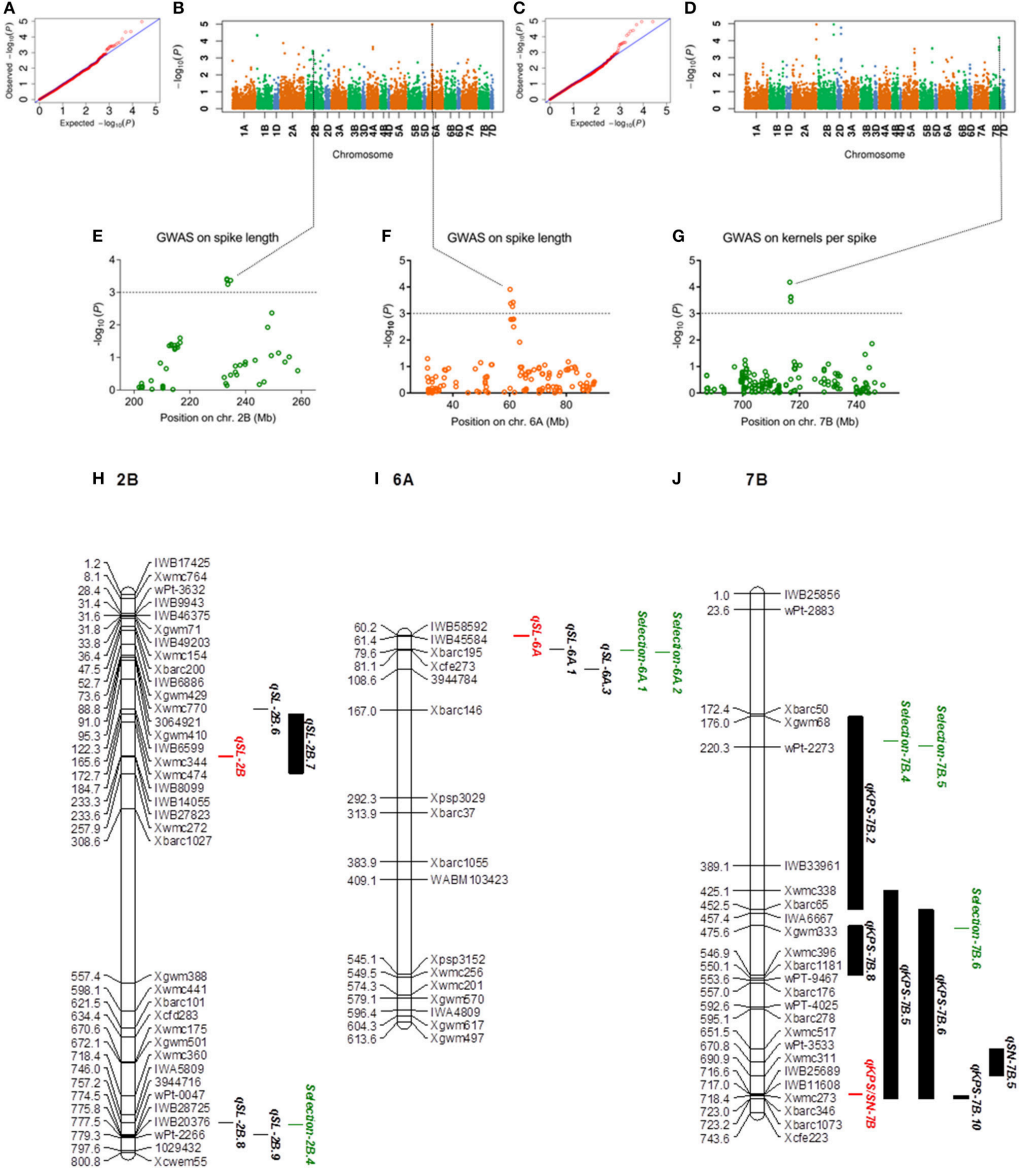
GWAS results for spike length and kernels per spike. **(A–D)** Q-Q plot and Manhattan plot of SNPs associated with spike length and kernels per spike in two environments. **(E–G)** Manhattan plots of the SNP clusters on chromosomes 2B, 6A, and 7B. The SNP clusters on 7B represented cKPS/SN-7B. The dashed horizontal line depicted a significant threshold level. **(H–J)** The integrated physical map of SNP clusters and reported QTLs. The short arms of the chromosomes are located at the top. The physical positions of the marker loci are listed on the left side of the corresponding chromosomes. The names of the marker loci and QTLs are listed on the right side of the corresponding chromosomes. Red bar: SNP clusters; green bar: selection regions; black bar: reported QTLs.

The five spike related traits in four environments were used to perform GWAS. QQ-plots and Manhattan plots of the GWAS results are shown in Figures 2A–G, Supplementary Figures 3–7. Fifty-one, Twenty-eight, Thirty-nine, Thirty, and Thirty-six significant SNPs were detected for SL, SN, KPS, TKW, and SNPP in all environments, respectively (Supplementary Tables 9–13). Among these significant SNPs, 18 loci were detected in two or more environments. Meanwhile, 31 SNP clusters (two more SNP in one LD decay distance) were found, and five SNPs in one cluster were multi-trait loci. Clusters contained four more SNPs or multi-environment loci as candidates were further studied. Therefore, four SNP clusters correlated to spike length located on chromosome 2A, 2B, 2D, 6A, and three SNP clusters associated with kernels per spike (including one multi-trait loci which also correlated to spikelet number) located on chromosome 2A, 2B, 7B were analyzed (Table 1).

**Figure 3:**
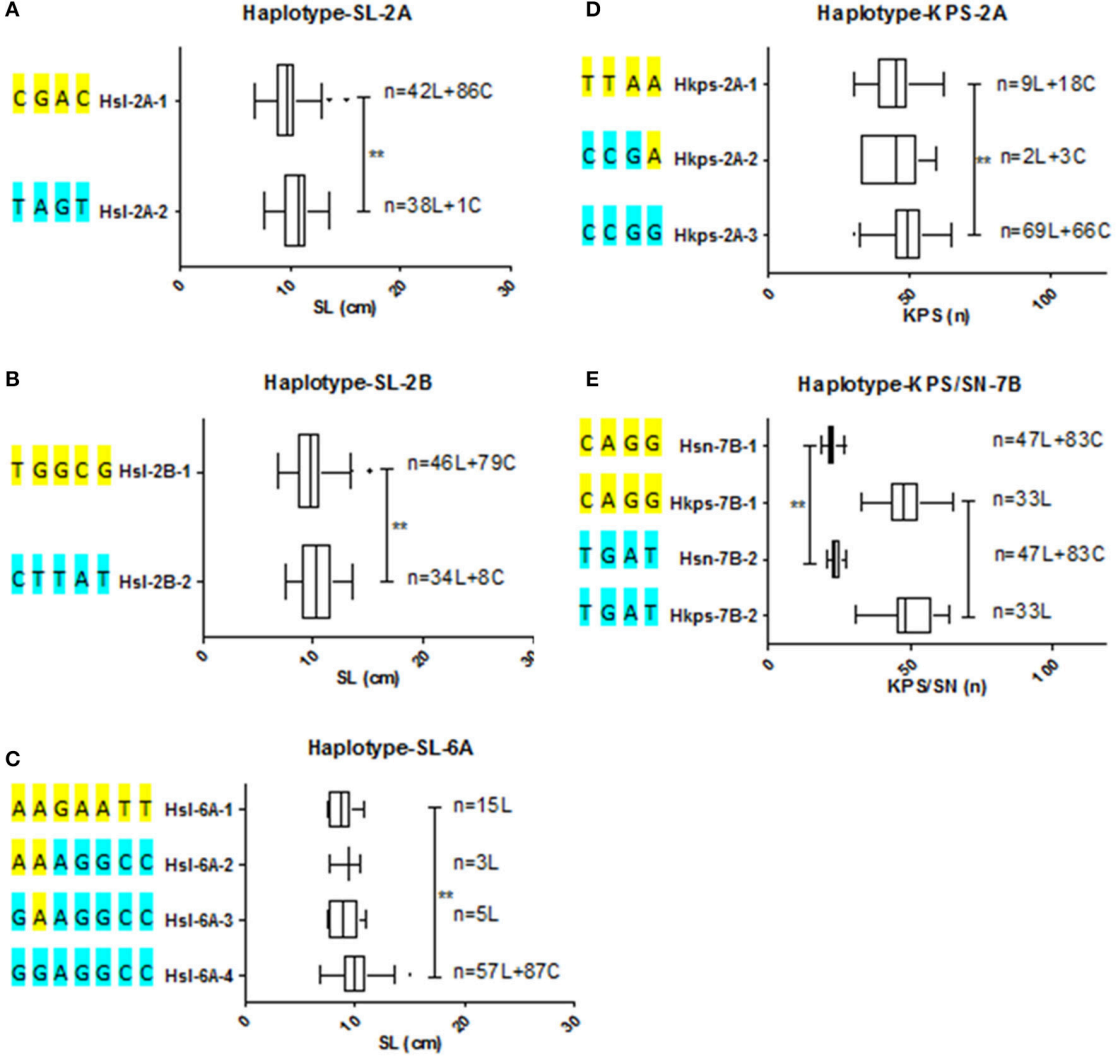
Haplotypes and their distribute frequency of SNP clusters among wheat natural population **(A–C)** Haplotypes of SNP clusters for SL on chromosome 2A, 2B, and 6A, respectively. **(D,E)** Haplotypes of SNP clusters for KPS and KPS/SN on chromosome 2A and 7B, respectively. Extermun value and mean value of traits are displayed by the box plot. Statistical significance was determined by LSD test: **P < 0.01. *n* denoted the number of genotypes belonging to the haplotype. SL, spike length; SN, spikelet number; KPS, kernels per spike; L, landrace; C, cultivar.

According to the types of these SNP clusters, haplotypes associated with spike related traits were detected. Meanwhile, the frequency of haplotypes distributed in germplasm was analyzed (Figure 3, Supplementary Figure 8). Two haplotypes associated with SL were identified for each cluster on chromosome 2A, 2B, and 2D, respectively. Hsl-2A-2 and Hsl-2B-2, the advantage haplotypes, show significant longer spike than that of Hsl-2A-1 and Hsl-2B-1. However, the spike length of the two haplotypes Hsl-2D-1 and Hsl-2D-2 did not display statistically difference. Hsl-2A-1 contained 42 landraces and 86 cultivars, while the advantage haplotype Hsl-2A-2 included 38 landraces and only one cultivar. Similarly, 46 landraces and 79 cultivars belonged to Hsl-2B-1, while the advantage haplotype Hsl-2B-2 contained 34 landraces and 8 cultivars. Four associated haplotypes were found for spike length related cluster on 6A (Hsl-6A-1~4). Hsl-6A-4 showed significant longer spike than that of Hsl-6A-1/2/3. The advantage haplotype (Hsl-6A-4) included all the cultivars (87) and more than half of the landraces (57). Meanwhile, 15, 3, and 5 landraces belong to Hsl-6A-1, Hsl-6A-2, and Hsl-6A-3, respectively. Three haplotypes associated with KPS were detected for clusters on 2A and 2B, respectively. Hkps-2A-3 shows significant more kernels per spike than that of Hkps-2A-1. However, the three haplotypes of Hkps-2B did not show significant difference. Hkps-2A-1 contained 9 landraces and 18 cultivars. Only 2 landraces and 3 cultivars belonged to Hkps-2A-2. The advantage haplotype, Hkps-2A-3, contained 69 landraces and 66 cultivars. The multi-trait cluster including two associated haplotypes with KPS/SN was detected on 7B (Hkps/sn-7B-1, Hkps/sn-7B-2). Forty-seven landraces and 83 cultivars were observed with hapoltype Hkps/sn-7B-1. None cultivars, but 33 landraces were found contains the advantage haplotype Hkps/sn-7B-2.

### SNP clusters and their overlapped QTLs were integrated

The significantly associated SNPs detected in this research were compared with the previously reported QTLs, markers or genes based on the physical positions. Five clusters and one SNP related to SL found in this study were located within the known QTL regions. Two multi-environment loci associated with SN overlapped with the reported QTLs. Three related clusters and one related SNP to KPS were mapped on the reported QTL regions. Two clusters and five SNPs related to TKW overlapped with the previously reported QTLs. One cluster, one multi-environment loci and one SNP related to SNPP were located within reported QTL regions, respectively (Supplementary Tables 9–13). In addition, the *TaGW2* homoeologues on 6D and 6A were physically covered one KPS and one TKW related SNP, respectively. Meanwhile, the *Vrn-A1* gene was physically located to one SNPP related SNP (Supplementary Tables 11–13).

The SNP clusters mentioned above (located on the chromosome 2A, 2B, 2D, 6A, and 7B) and spike related QTLs reported previously were integrated on the physical maps (Figures 2H,J, Supplementary Figure 9, Supplementary Table 14). The results showed that SNP cluster *qSL-2B* (SNP cluster associated with spike length on 2B) was covered by the reported QTL *qSL-2B.7* (Figure 2H)*. qKPS/SN-7B* (SNP cluster associated with KPS/SN on 7B) was covered by the reported QTLs *qKPS-7B.5* and *qKPS-7B.6* (Figure 2J). *qSL-2A* and *qKPS-2A* detected in this research overlapped with the reported QTL *qSL-2A.2* and *qKPS-2A.8*, respectively. *qKPS-2B* found in this study was covered by the reported QTL *qKPS-2B.12*. the SNP cluster *qSL-2D* overlapped with the reported QTL *qSL-2D.9* (Supplementary Figure 9). Meanwhile, the SNP cluster *qSL-6A* was close to the reported QTL *qSL-6A.1*, and *qTKW-2D* reported herein was close to the reported QTLs *qTKW-2D.23* and *qTKW-2D.25* (Supplementary Figure 9, Figure 2I). In addition, the artificial selection regions (on 2A, 2B, 2D, 6A, and 7B) covered by the reported QTLs were also integrated on the maps. Notably, the selection region *Selection-2A.7* was covered by the cluster *qSL-2A* reported herein and the QTL *qSL-2A.2* reported previously. Similarly, the *selection-2D.2* and *selection-2D.3* overlapped with the reported QTLs (*qSL-2D.9* and *qTKW-2D.25*). Meanwhile, they were physically closed to the SNP clusters, *qSL-2D* and *qTKW-2D*.

### Candidate genes for artificial selection and GWAS

Genes in the selected window clusters or windows detected by two methods were considered as candidate genes for artificial selection during wheat improvement. To annotate these genes, the wheat, rice and *Arabidopsis* functional gene databases were used. The result revealed that some of these genes were involved in selection-related agronomic traits such as stress response, development (seed size, seed number, seed maturation, seed dormancy, and flowering time) (Supplementary Figure 10A, Supplementary Table 15).

One Mb flanking regions of the above-mentioned SNP clusters that detected by GWAS were defined as candidate regions based on LD decay. Genes located in the candidate regions were identified as candidate genes. These genes were annotated by using the same methods mentioned above. The results showed that these genes were mainly involved in the function such as metabolism, transcription, stress response, development and so on (Supplementary Figure 10B, Supplementary Table 16).

### KASP assay

**Figure 4:**
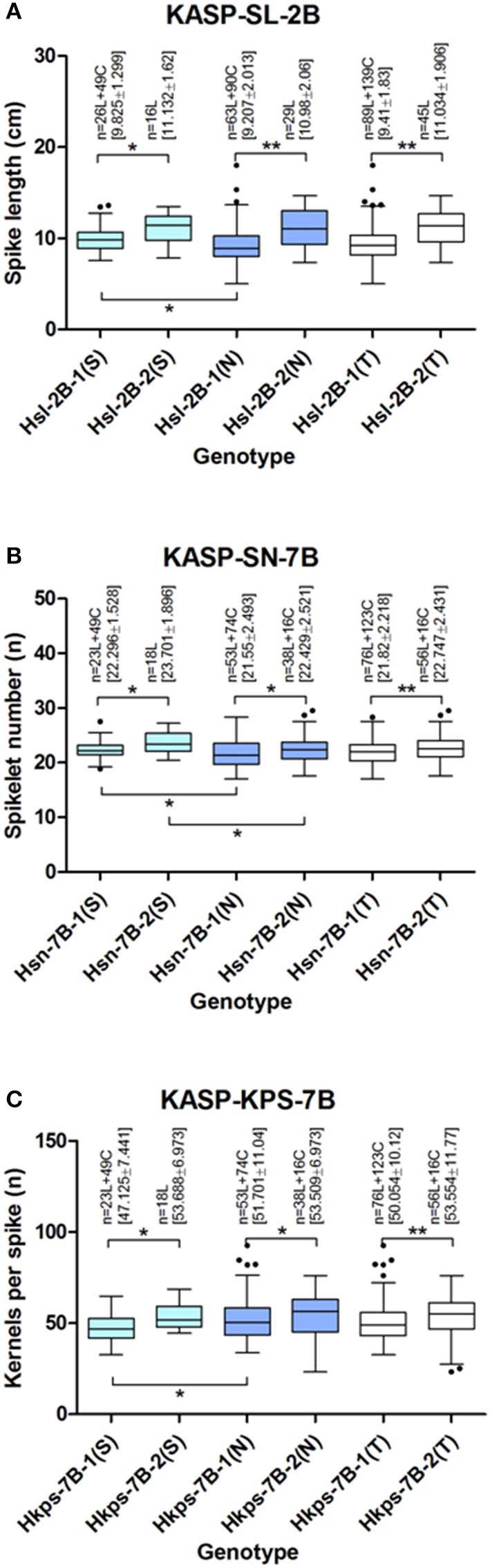
Frequency of haplotypes detected by KASP markers in wheat germplasm **(A–C)** Frequency of haplotypes related to SL, SN/KPS on chromosome 2B and 7B, respectively. Extermun value and mean value of traits are displayed by the box plot. Statistical significance was determined by LSD test: *P < 0.05, **P < 0.01. *N* denoted the number of genotypes belonging to each haplotype. SL, spike length; SN, spikelet number; KPS, kernels per spike; L, landrace; C, cultivar; S, south China samples; N, north China samples; T, total samples.

SNPs from these hapoltypes were used to develop KASP markers (Supplementary Table 3). A total of 276 wheat lines across south and north China were genotyped by these KASP markers (Supplementary Figure 11). The results demonstrated that genotypes from KASP test were identical to the chip assay. The frequency and significant difference of these haplotypes in germplasm were analyzed (Figure 4). Compared to Hsl-2B-1 (9.41 ± 1.83 cm) which contains 89 landraces and 139 cultivars, Hsl-2B-2 contains 45 landraces and shows significantly longer spike (11.034 ± 1.906 cm). The average kernels per spike (53.554 ± 11.77) and spikelet number (22.747 ± 2.431) of Hkps/sn-7B-2, the advantage allele, showed significantly higher than that of Hkps/sn-7B-1 (kernels per spike: 50.054 ± 10.12; spikelet number: 21.82 ± 2.218). Hkps/sn-7B-2 included 56 landraces and 16 cultivars, while 76 landraces and 124 cultivars were identified to Hkps/sn-7B-1. In addition, the spike length of south China lines was significantly longer than that of north China for Hsl-2B-1. The similar result was found in Hkps-7B-1, but the spikelet number of south China lines was significantly higher than that of north China for both haplotypes of Hsn-7B.

## Discussion

Conventional breeding, mostly based on the phenotypic selection, has undergone several centuries. Despite the huge improvement of artificial selection, blindness, empirical, inefficiency and long time costs of conventional breeding blocked the wheat yield increasing. The wheat genome sequence, released on the IWGSC website, could promote the rapid improvement of cultivars by efficient using genetic resources and genomic breeding. Molecular breeding would lead the trend of technological development from SSR to SNP level (Liu et al., 2017). In consideration of the modularity of biological process, rational design modules resulting in predictable functions might become the key step for molecular breeding (Gavin et al., 2006; Silver et al., 2014). For example, 14 engineered *CLV3* promoter alleles targeted editing by CRISPR/Cas9 causes a continuum of locule number variation in tomato (Somssich et al., 2016; Rodríguez-Leal et al., 2017).

### Wheat yield improvement is the main aim of artificial selection

During the past several centuries, crop has undergone the domestication and selection in order to increase the yield. As a result, lots of advantage genes/hapoltypes were picked up and inherited. In this research, artificial selection signal analysis was conducted in the wheat natural population which contained landraces and cultivars. As the result, a lot of window clusters were detected for the selective sweep candidates that tended to occur in clusters (Gao et al., 2017). Fortunately, lots of candidate genes involved in improvement-related agronomic traits such as stress response, seed size, seed number, seed maturation, seed dormancy and flowering time were obtained in the selective sweep regions. The results suggest that increasing wheat yield, as the most powerful evolutionary force, has created superior genotypes by phenotype selection and fixed in cultivars such as bigger seed size, longer spike, shorter plant height, and more kernels number per spike (Yan et al., 2018).

To testify the usefulness of the selection analysis in the worldwide collection, the candidate sweep regions were compared with the previously reported QTLs, markers and genes related with the investigated traits. In total, 81 windows were overlapped with the known improvement agricultural related traits in different wheat populations from all over the world (Figure 1, Supplementary Table 8). Fortunately, two window clusters on 2A and 6B overlapped with the reported yield related gene *TaUBP24* (Liu K. et al., 2018) and QTL for thousand kernel weight and spike length (Wang et al., 2011; Mir et al., 2012), respectively. This would be the strong evidence for the general applicability of this method.

### SNP clusters related to spike traits and overlapped QTLs were integrated

A lot of QTLs associated with spike related traits have been detected from genetic populations. These QTLs spread all over the 21 chromosomes (Liu K. et al., 2018). However, because of the genetic background and lack of genomic information, few of these QTLs have been used in wheat improvement. In this study, interested SNP clusters related to spike traits on chromosome 2A, 2B, 2D, 6A, and 7B detected by GWAS which were analyzed based on a broad genetic background. According to the genome sequence information, these SNP clusters were located on wheat genome. Meanwhile, at these regions, several QTLs associated with the same phenotype were found.

KPS is the key component of wheat yield and spikelet number is the main factor affected the kernels per spike (Liu K. et al., 2018). *qKPS/SN-7B*, the multi-trait locus located on 7B, significantly associated with both KPS and SN. Pleiotropy, a single gene or QTL associated with multi-trait, has been proved in many previous reports (Neumann et al., 2011). The haplotype Hkps/sn-7B-2 only contained landraces, which indicated the advantage haplotype has not been transit into cultivars and the necessary usage of the haplotype in breeding. The multi-trait locus *qKPS/SN-7B* has been confirmed in previous studies and overlapped with *qKPS-7B.1, qKPS-7B.5*, and *qKPS-7B.6* (Wang et al., 2011; Cui et al., 2014; Liu et al., 2014; Yu et al., 2018). However, the QTL regions were too wide (270~571 Mb) to predict candidate genes. SNP cluster *qKPS/SN-7B* reported herein covered a very narrow region (0.37 Mb). Based on the LD decay, the candidate region contained only 55 candidate genes (Supplementary Tables 14, 16). The candidate gene, TraesCS7B01G456300, overlapped with the leading SNP encoded a BURP domain-containing protein. BURP genes broadly exist in plants, which have been proved to be the contributors for seed development, seed size, seed mass and seed number (Van Son et al., 2009; Xu et al., 2013). According to the results, TraesCS7B01G456300 which encoded the BURP domain-containing protein might be the candidate gene to control KPS and SN.

By the same way, advantage haplotype Hkps-2A-3 for the SNP cluster associated with KPS on 2A contained the majority landraces and cultivars, which indicated that the advantage haplotype has been effectively transit into modern cultivars. Compared with the reported QTLs associated with KPS on chromosome 2A, *qKPS-2A.8* overlapped with the cluster (Jia et al., 2013). The QTL region defined by the flanking markers for *qKPS-2A.8* was relatively narrow (42.9 Mb), but it was also difficult to detect the candidate genes. By contrast, the SNP cluster *qKPS-2A* covered region (0.01 Mb) together with the LD region only including 33 candidate genes (Supplementary Tables 14, 16).

Spike length as an indirect factor also affects KPS and plays important role in improving wheat yield (Guo et al., 2017). The advantage haplotypes of Hsl-2A and Hsl-2B contained only few cultivars, which mean the invalid transit into cultivars and the necessary usage of the two haplotypes in breeding. All modern cultivars contained this advantage haplotype Hsl-6A-4 which suggested that this haplotype has been effectively used in artificial selection. Spike length related QTLs on chromosome 2A, 2B, and 6A were integrated on a physical map. Interestingly, the SNP cluster *qSL-2A* was overlapped with reported QTL *qSL-2A.2* (Liu et al., 2017) and annotated the selection signal *Selection-2A.7*. Among the candidate genes, there were two genes, TraesCS2A01G130200 and TraesCS2A01G130600, overlapped with the leading SNPs (Supplementary Table 16). The homologous genes of rice were *OsHAD1* and *OsGS1*, respectively. Overexpresion of *OsHAD1* in rice resulted in enhanced phosphatase activity and biomass (Pandey et al., 2017). Co-overexpression of *OsGSA1* and *OsGSA2* in rice could increase tiller number, panicle number, and grain filling, and result in yield improvement (James et al., 2018). According the results of rice, these two genes may be both important for wheat to improve the SL trait.

### Two advantage haplotypes for wheat yield improvement

Detection of allelic variations is the first step to crop improvement and identification of advantage haplotype is crucial for breeding (Hou et al., 2014). Based on the GWAS results, several SNP clusters related to spike traits were detected and haplotypes were found in a 192 wheat collections. In order to use the advantage haplotypes, four pairs of KASP markers around Hsl-2B, Hsl-6A Hkps/sn-7B were successfully developed based on the SNPs. Larger wheat population including partial of the GWAS accessions and some northern China wheat germplasm was tested. The genotypes identified by KASP platform were consistent with the 90 K SNP chip which indicates KASP markers could be used for haplotypes detection (Tan et al., 2017).

According to the history of artificial selection, north China breeding paid more attention to spike number and south China is mainly on kernels per spike. This selection strategy is adapted to the humid environment as well as wheat disease which not usually occurred in north China (Kang et al., 2011). As a result, cultivars in south China show a larger spike (longer SL, higher SN, and KPS) than that of north China after the artificial selection. Hsl-2B-2, the advantage haplotype for increasing spike length on chromosome 2B, has not been detected in modern cultivars. Wheat lines contain this advantage haplotype show significant longer spike both in south and north China. After transition of this haplotype into the modern cultivars by using the KASP marker in breeding program, the spike length would be significantly increased in both south and north China. Hsn-7B-2, the advantage haplotype for increasing the spikelet number, has been partially transited into lines in north China but none into lines in south China. Meanwhile, both in south and north China, lines contain this advantage haplotype show significant higher spikelet number. Furthermore, for lines contain this advantage haplotype, the spikelet number in south China is significant more than that in north China. This result suggested transition of this haplotype in south cultivars could significantly increase their spikelet number, which similar to north China. Similar to Hsn-7B-2, the transition of Hkps-7B-2 into south cultivars, the advantage haplotype for increasing the kernels per spike, would significantly increase its KPS in our breeding program.

Hkps/sn-7B, multi-trait locus, detected in this research could both increase the spikelet number and kernels per spike. This result could be proved by the significantly correlation between kernels per spike and spikelet number (coevolution) during selection (Michel et al., 2018). The advantage haplotype of Hkps/sn-7B could be introduced into modern cultivar to increase kernels per spike and spikelet number once by KASP marker.

## Conclusion

In summary, seven SNP clusters involved in wheat spike traits were detected by selection signal analysis and GWAS. Based on the released wheat genome sequence, an integrated map which contains the SNP clusters and their overlapped/flanking QTLs was constructed. KASP markers to identify two advantage haplotypes were developed for increasing SL, KPS and SN in further breeding program.

## Author contributions

JL, BF, and ZX designed the research. JL, ZX, BF, XF, QZ, JC, FW, GJ, and LY conducted phenotype of the wheat population. JL analyzed the data and wrote the manuscript; Funding was acquired by TW. BF and TW had primary responsibility for final content. All authors contributed to manuscript revision, read and approved the submitted version.

### Conflict of interest statement

The authors declare that the research was conducted in the absence of any commercial or financial relationships that could be construed as a potential conflict of interest.

## Abbreviations

ANOVA: Analysis of Variance
CTAB: Cetyl Trimethyl Ammonium Bromide
CMLM: Compressed Mixed Linear Model
GWAS: Genome-Wide Association Study
HWE: Hardy-Weinberg Equilibrium
KASP: Kompetitive Allele Specific PCR
KPS: Kernels per Spike
LD: Linkage Disequilibrium
MAF: Minor Allele Frequency
MAS: Marker-Assisted Selection
QTL: Quantitative Trait Locus
SL: Spike Length
SN: Spikelet Number
SNP: Single Nucleotide Polymorphism
SNPP: Spike number per plant
TKW: Thousand kernel weight.
