# Supplementary material for: A Genome-Wide Association Study of Wheat Spike Related Traits in China
Source: Front Plant Sci. 2018 Oct 31;9:1584. doi: 10.3389/fpls.2018.01584 (PMC6220075; doi:10.3389/fpls.2018.01584)
Supplement: Supplementary file 1 [file Data_Sheet_1.docx]

*Supplementary Material*

**A genome-wide association study of wheat spike related traits in China**

Jing Liu^1^, Zhibin Xu^1^, Xiaoli Fan^1^, Qiang Zhou^1^, Jun Cao^1^, Fang Wang^1^, Guangsi Ji^1^, Li Yang^1^, Bo Feng^1*^ and Tao Wang^1*^

^1^Chengdu Institute of Biology, Chinese Academy of Sciences, Chengdu, China.

***Correspondence:**Dr. Bo Feng
[fengbo@cib.ac.cn](mailto:fengbo@cib.ac.cn).

Dr. Tao Wang
[wangtao@cib.ac.cn](mailto:wangtao@cib.ac.cn).

# Supplementary Figures

**Supplementary Figure 1** Frequency distribution of phenotypic variation for tested traits in different environments. SL: spike length, SN: spikelet number, KPS: kernels per spike, TKW: thousand kernel weight, SNPP: spike number per plant.

**
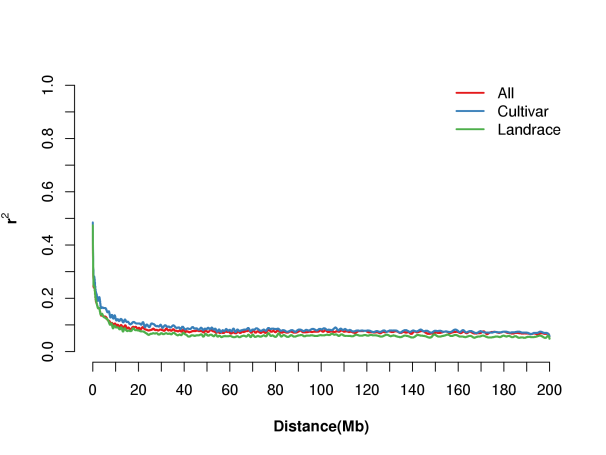
**

**Supplementary Figure 2** LD Decay in wheat genome. The decay of LD was measured by the *r*^2^ against the distance between polymorphic sites in all samples (red), cultivars (blue) and landraces (green). LD dropped to half of its maximum value at 888 kb, 785 kb and 1053 kb for all the samples, landraces and cultivars, respectively.

**
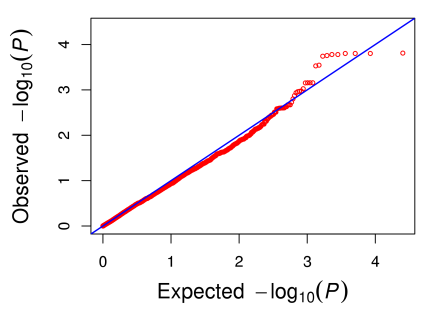

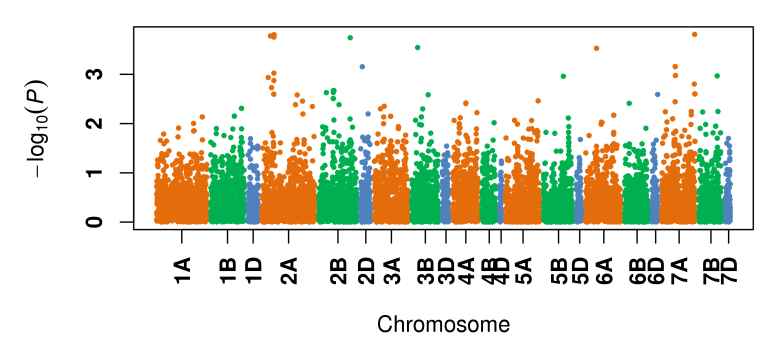

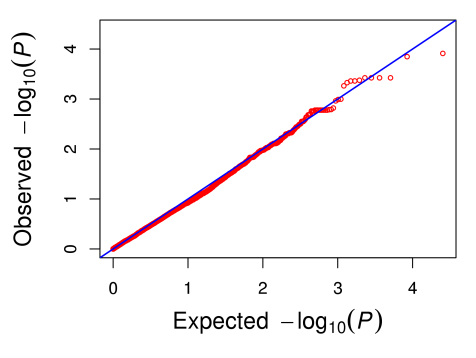

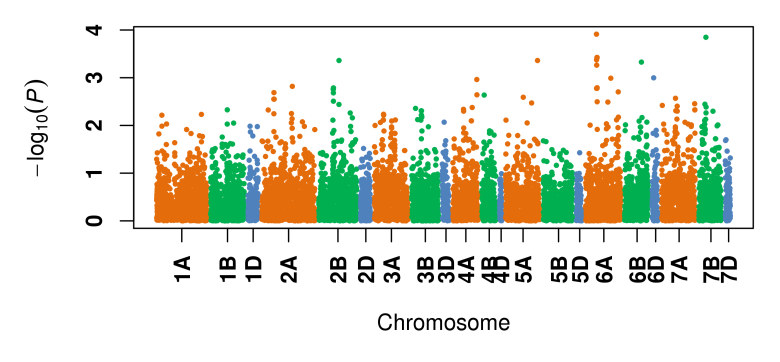
**

**E2**

**E1**

**
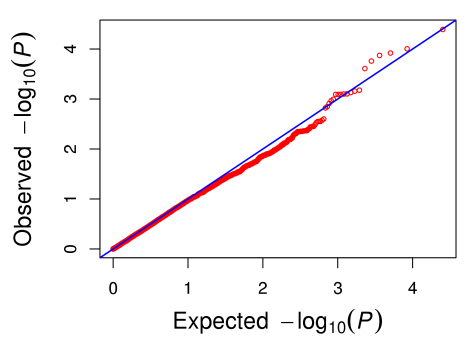

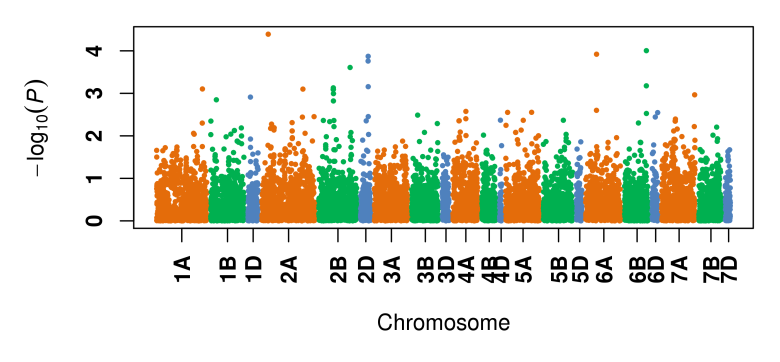
**

**E3**

**Supplementary Figure 3** Q-Q plot and Manhattan plot of SNPs associated with spike length in other three environments. E1: 2015-2016 Shifang with low nitrogen treatment, E2: 2015-2016 Shifang with high nitrogen treatment, E3: 2015-2016 Shuangliu.

**
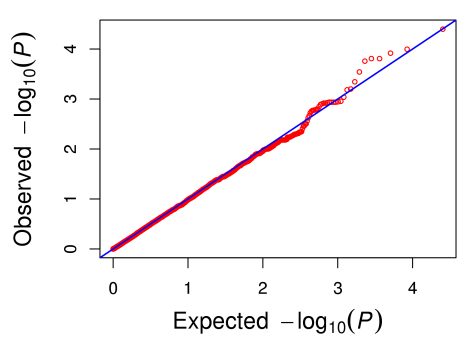

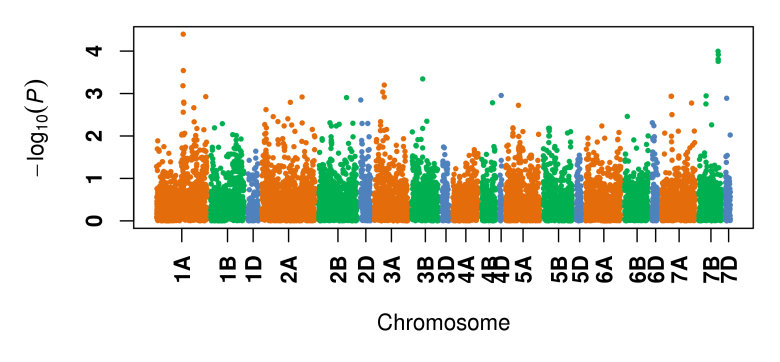
**

**E1**

**
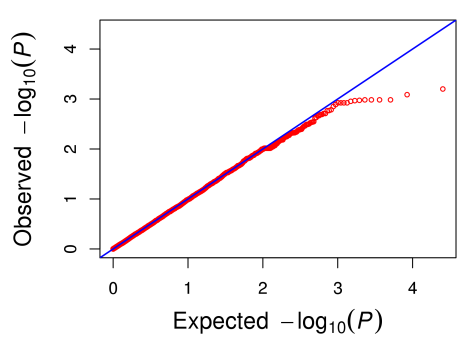

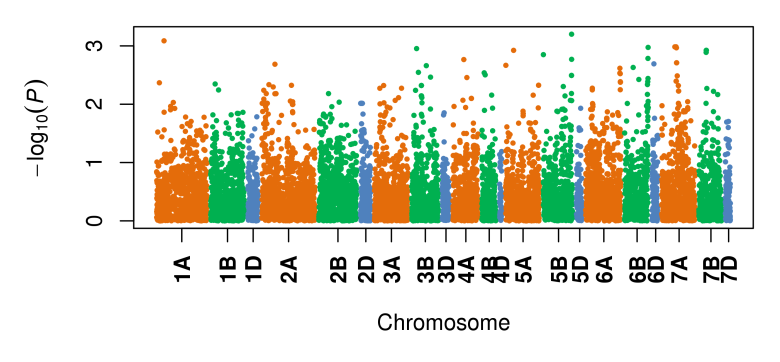
**

**E2**

**
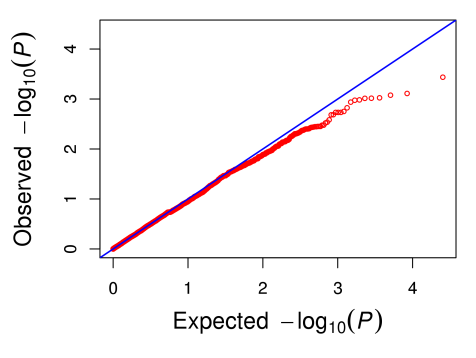

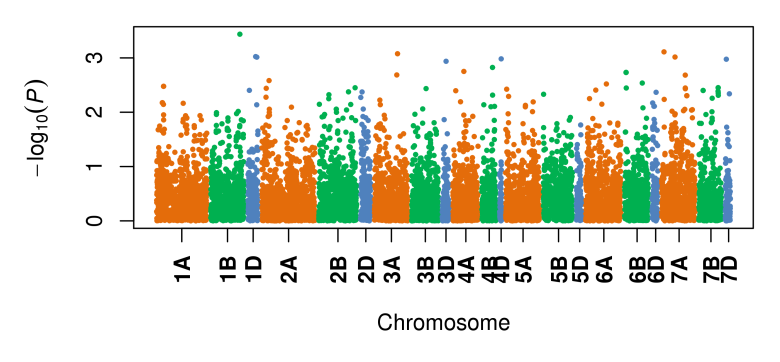
**

**E3**

**
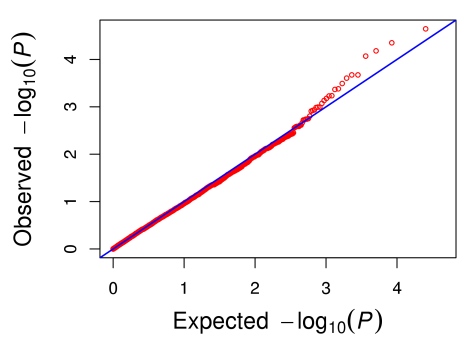

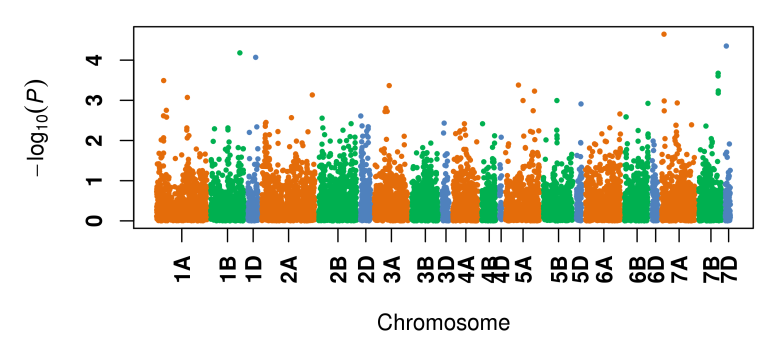
**

**E4**

**Supplementary Figure 4** Q-Q plot and Manhattan plot of SNPs associated with spikelet number in four environments. E1: 2015-2016 Shifang with low nitrogen treatment, E2: 2015-2016 Shifang with high nitrogen treatment, E3: 2015-2016 Shuangliu, E4: 2014-2015 Shuangliu.

**
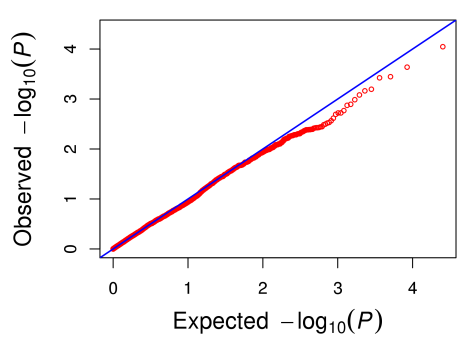

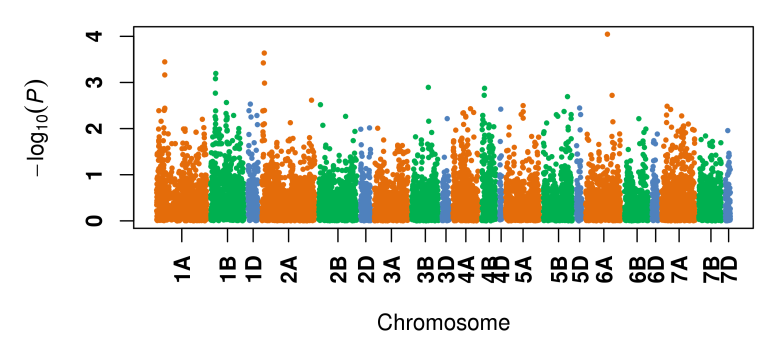
**

**E2**

**
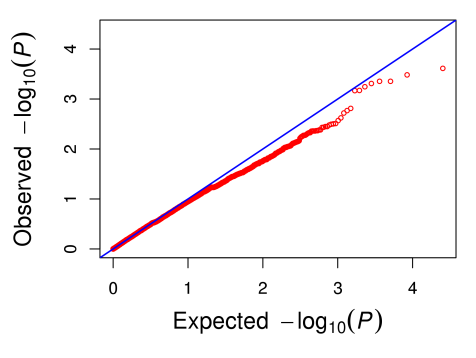

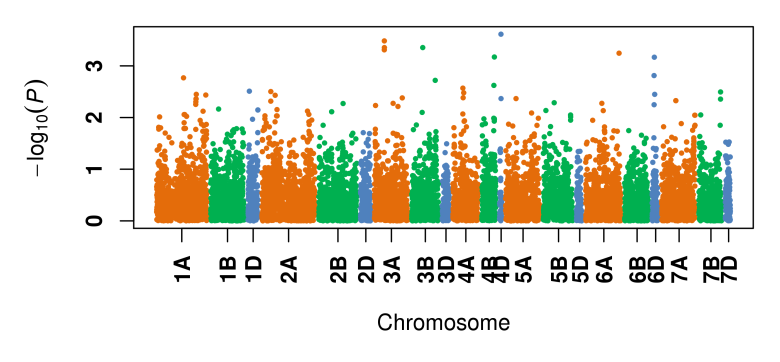
**

**E3**

**
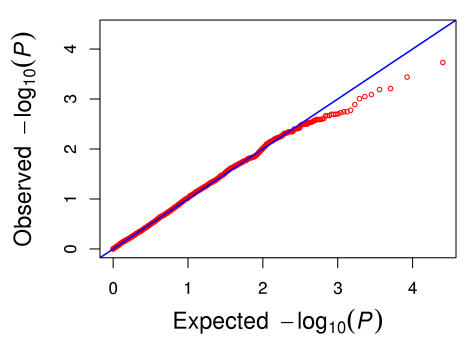

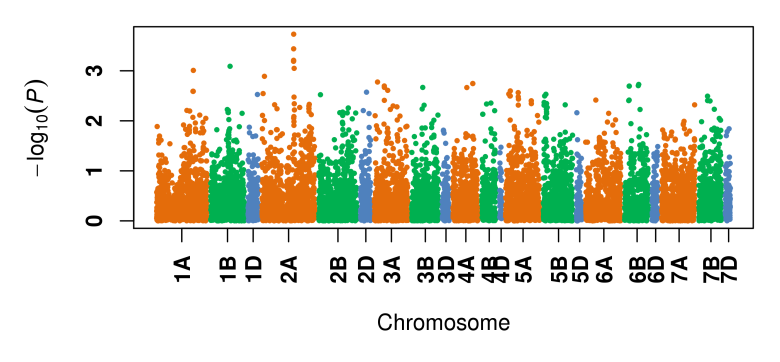
**

**E4**

**Supplementary Figure 5** Q-Q plot and Manhattan plot of SNPs associated with kernels per spike in other three environments. E2: 2015-2016 Shifang with high nitrogen treatment, E3: 2015-2016 Shuangliu, E4: 2014-2015 Shuangliu.

**
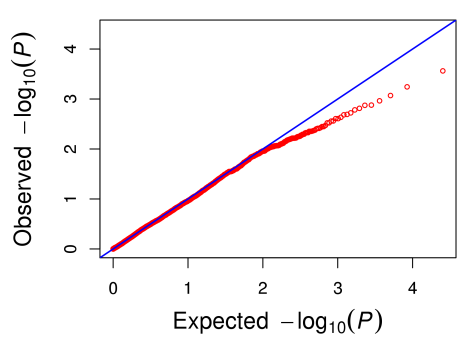

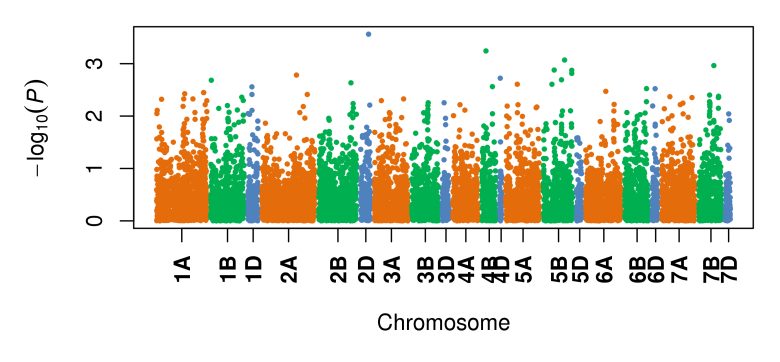
**

**E1**

**
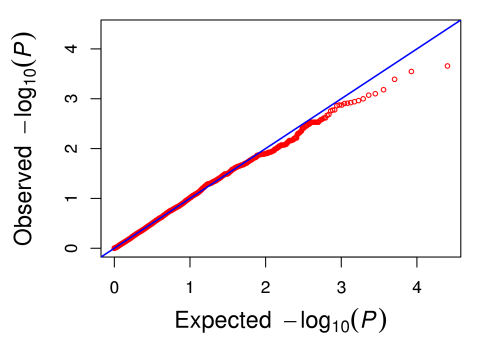

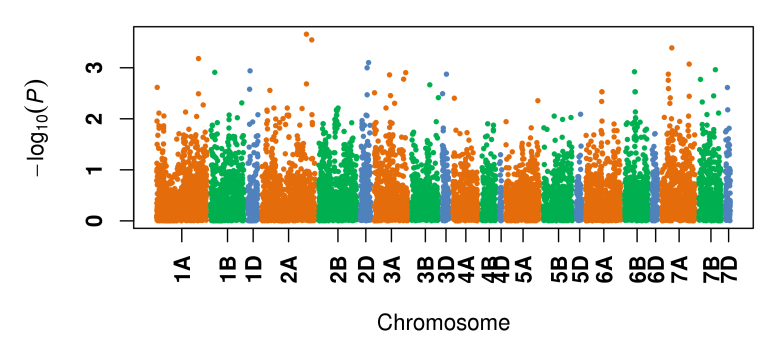
**

**E2**

**
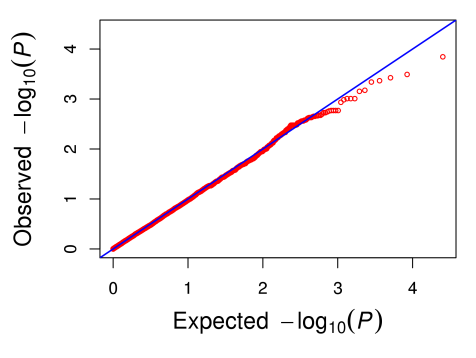

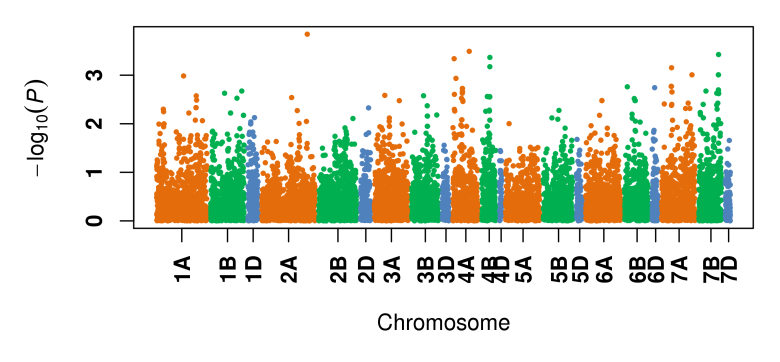
**

**E3**

**
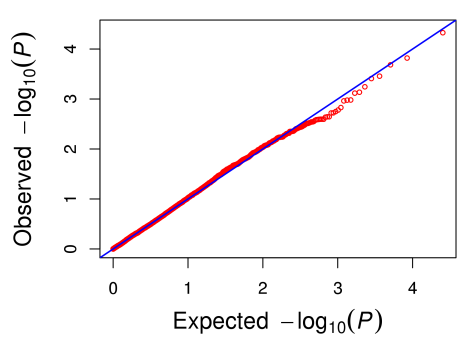

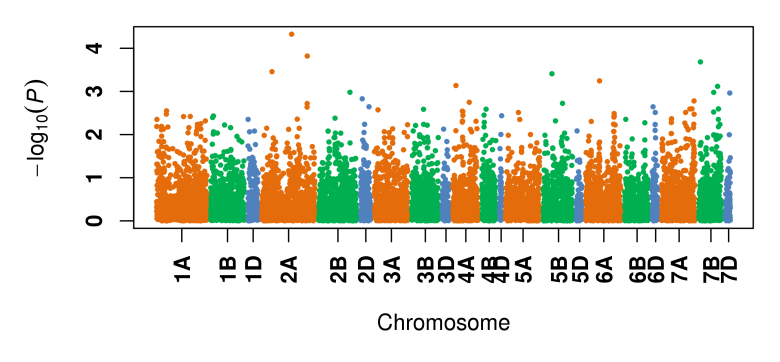
**

**E4**

**Supplementary Figure 6** Q-Q plot and Manhattan plot of SNPs associated with thousand kernel weight in other three environments. E1: 2015-2016 Shifang with low nitrogen treatment, E2: 2015-2016 Shifang with high nitrogen treatment, E3: 2015-2016 Shuangliu, E4: 2014-2015 Shuangliu.

**
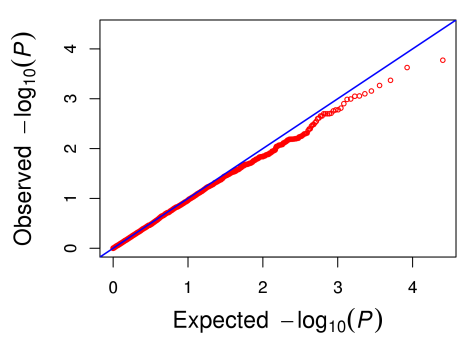

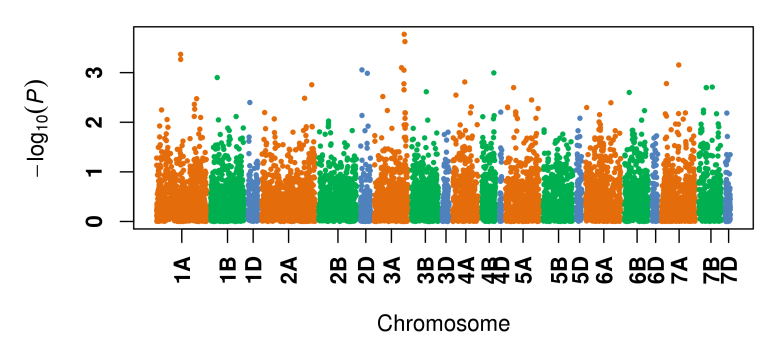
**

**E1**

**
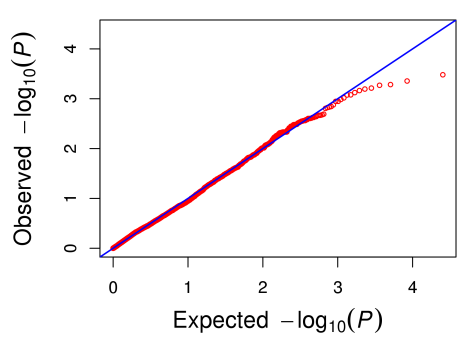

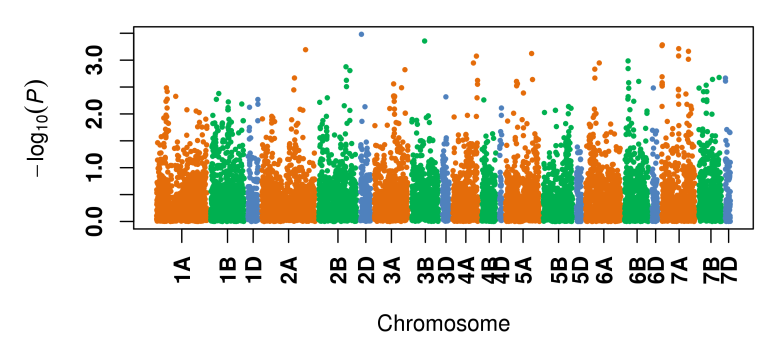
**

**E2**

**
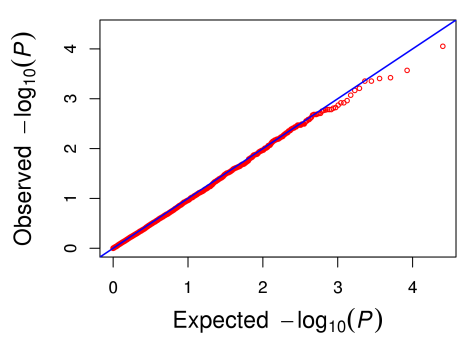

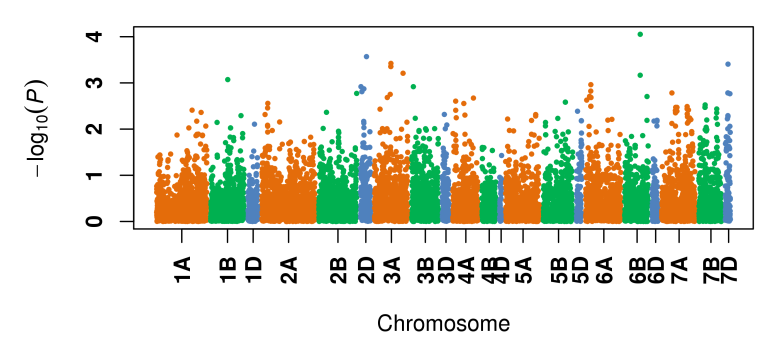
**

**E3**

**
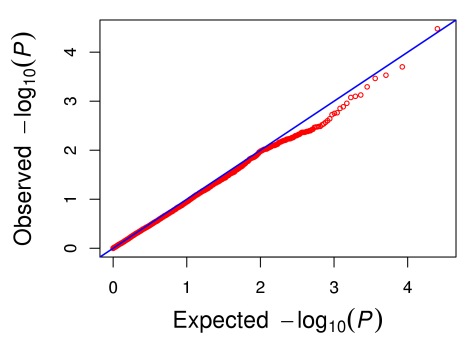

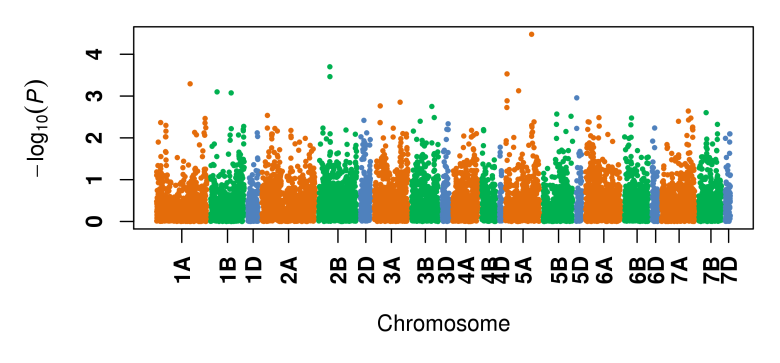
**

**E4**

**Supplementary Figure 7** Q-Q plot and Manhattan plot of SNPs associated with spike number per plant in other three environments. E1: 2015-2016 Shifang with low nitrogen treatment, E2: 2015-2016 Shifang with high nitrogen treatment, E3: 2015-2016 Shuangliu, E4: 2014-2015 Shuangliu.

**Supplementary Figure 8** Haplotypes and their distribute frequency of SNP clusters among wheat natural population**.** (a) Haplotypes of SNP clusters for SL on chromosome 2D. (b) Haplotypes of SNP clusters for KPS on chromosome 2B. Extermun value and mean value of traits are displayed by the box plot. Statistical significance was determined by LSD test: ** *P*<0.01. n denoted the number of genotypes belonging to the haplotype. SL: spike length, SN: spikelet number, KPS: kernels per spike, L: landrace, C: cultivar.

**Supplementary Figure 9** The integrated physical map of SNP clusters and reported QTLs. The short arms of the chromosomes are located at the top. The physical positions of the marker loci are listed on the left side of the corresponding chromosomes. The names of the marker loci and QTLs are listed on the right side of the corresponding chromosomes. Red bar: SNP clusters; green bar: selection region; black bar: reported QTLs.

(a)

(b)

**Supplementary Figure 10** (a) Bar chart for biological function annotations of candidate genes identified by selection signal analysis (b) Bar chart for biological function annotations of candidate genes identified by GWAS.

**
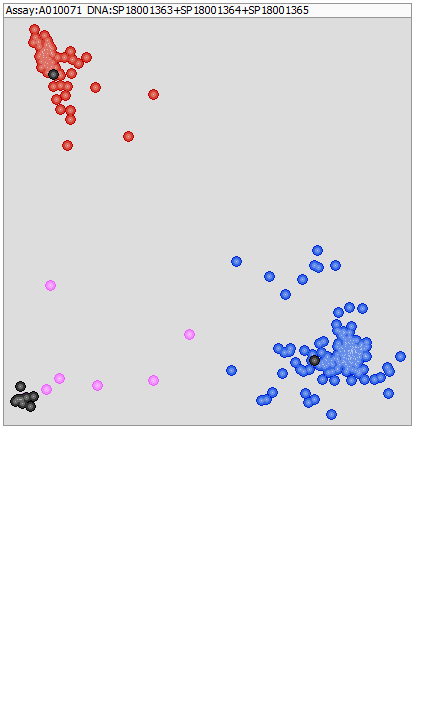

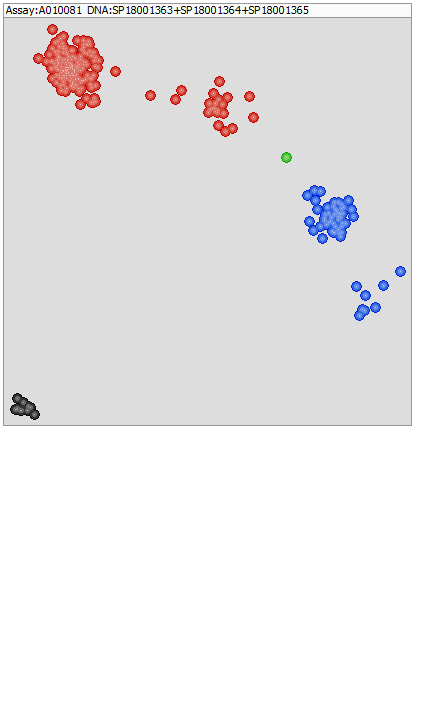

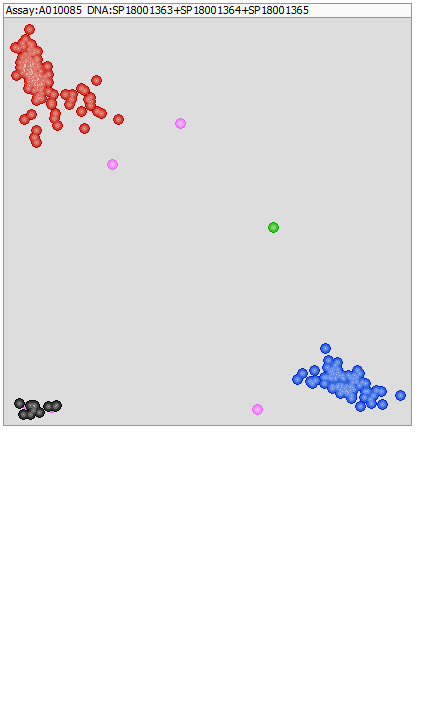

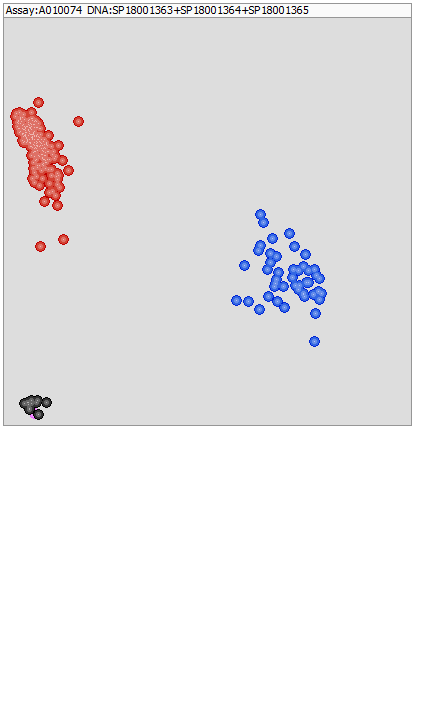
**

**Supplementary Figure 11** Scatter plots of representative KASP-assays. Blue and red dots indicate genotypes with homozygous alleles, green dots for heterozygous, pink dots for samples with no signals, and black dots shows NTC (No template controls).

# Supplementary Tables

**Supplementary Table 1** The information of the 192 wheat lines used for GWAS

| **NO.** | **Lines** | **Name** | **Evolutionary Types** | **AVE-SL (cm)** | **AVE-SN (n)** | **AVE-KPS (n)** | **AVE-TKW (g)** | **AVE-SNPP (n)** |
| --- | --- | --- | --- | --- | --- | --- | --- | --- |
| 1 | WT-001 | ZM08807 | landrace | 9.96 | 27.00 | 45.87 | 24.14 | 8.93 |
| 2 | WT-002 | ZM08814 | landrace | 11.11 | 22.83 | 57.42 | 33.83 | 8.33 |
| 3 | WT-003 | ZM08815 | landrace | 9.98 | 20.33 | 46.38 | 40.01 | 5.83 |
| 4 | WT-004 | ZM08816 | landrace | 8.80 | 22.44 | 53.56 | 27.98 | 7.36 |
| 5 | WT-005 | ZM08817 | landrace | 10.47 | 24.50 | 62.33 | 22.76 | 11.11 |
| 6 | WT-006 | ZM08818 | landrace | 10.73 | 19.00 | 47.17 | 43.51 | 5.78 |
| 7 | WT-007 | ZM08820 | landrace | 9.86 | 21.89 | 58.75 | 21.42 | 12.61 |
| 8 | WT-008 | ZM08824 | landrace | 10.67 | 22.72 | 60.58 | 25.47 | 8.44 |
| 9 | WT-009 | Guangtoumai | landrace | 9.62 | 20.28 | 44.56 | 30.59 | 7.50 |
| 10 | WT-010 | ZM11532 | landrace | 9.46 | 20.11 | 56.50 | 30.47 | 10.44 |
| 11 | WT-011 | wumanghonghuamai | landrace | 10.89 | 22.11 | 57.89 | 35.54 | 10.22 |
| 12 | WT-012 | Bendihuanghuamai | landrace | 13.44 | 25.44 | 59.72 | 23.52 | 9.31 |
| 13 | WT-013 | ZM11799 | landrace | 9.60 | 22.33 | 54.31 | 31.43 | 10.22 |
| 14 | WT-014 | ZM11805 | landrace | 7.70 | 24.00 | 38.03 | 21.07 | 7.44 |
| 15 | WT-015 | ZM11806 | landrace | 8.86 | 27.44 | 55.75 | 23.57 | 5.67 |
| 16 | WT-016 | ZM11812 | landrace | 10.93 | 25.00 | 56.86 | 25.17 | 8.28 |
| 17 | WT-017 | ZM11855 | landrace | 11.53 | 22.00 | 52.75 | 29.88 | 7.44 |
| 18 | WT-018 | ZM11856 | landrace | 9.81 | 20.67 | 46.03 | 31.45 | 8.11 |
| 19 | WT-019 | ZM11857 | landrace | 9.71 | 21.94 | 48.06 | 28.93 | 8.83 |
| 20 | WT-020 | ZM11870 | landrace | 7.64 | 22.61 | 46.72 | 27.24 | 8.22 |
| 21 | WT-021 | ZM11891 | landrace | 11.30 | 23.07 | 61.63 | 34.17 | 7.07 |
| 22 | WT-022 | ZM11930 | landrace | 12.49 | 23.39 | 49.61 | 27.45 | 11.44 |
| 23 | WT-023 | ZM11931 | landrace | 9.58 | 20.83 | 45.14 | 42.03 | 6.61 |
| 24 | WT-024 | ZM11947 | landrace | 8.19 | 25.11 | 64.06 | 30.76 | 8.33 |
| 25 | WT-025 | ZM11948 | landrace | 9.36 | 23.50 | 54.06 | 31.06 | 8.42 |
| 26 | WT-026 | ZM11955 | landrace | 11.24 | 23.67 | 57.22 | 32.19 | 8.39 |
| 27 | WT-027 | ZM11960 | landrace | 11.45 | 21.11 | 38.00 | 37.99 | 7.94 |
| 28 | WT-028 | ZM11994 | landrace | 9.72 | 27.17 | 57.53 | 21.10 | 7.17 |
| 29 | WT-029 | ZM12064 | landrace | 10.27 | 21.06 | 57.44 | 32.89 | 8.50 |
| 30 | WT-030 | ZM12070 | landrace | 8.66 | 25.11 | 48.58 | 25.45 | 7.17 |
| 31 | WT-031 | ZM12072 | landrace | 7.98 | 23.11 | 50.11 | 30.14 | 7.39 |
| 32 | WT-032 | ZM12073 | landrace | 8.76 | 26.00 | 45.89 | 30.58 | 7.39 |
| 33 | WT-033 | ZM12075 | landrace | 10.48 | 21.89 | 56.67 | 34.47 | 6.94 |
| 34 | WT-034 | Dayouzi | landrace | 7.70 | 25.44 | 44.86 | 25.37 | 7.39 |
| 35 | WT-035 | Wuhuatou | landrace | 12.15 | 26.22 | 52.58 | 29.33 | 8.47 |
| 36 | WT-036 | Hongyouzi | landrace | 9.57 | 25.00 | 59.25 | 30.26 | 8.44 |
| 37 | WT-037 | Hongchunmai | landrace | 10.02 | 20.39 | 32.64 | 36.09 | 8.44 |
| 38 | WT-038 | Hongmangmai | landrace | 11.38 | 22.00 | 35.14 | 33.18 | 6.28 |
| 39 | WT-039 | Heshangtou | landrace | 11.82 | 22.42 | 35.64 | 34.33 | 10.69 |
| 40 | WT-040 | Jinghuangmai | landrace | 11.87 | 22.39 | 39.61 | 36.53 | 8.94 |
| 41 | WT-041 | Dahongmai | landrace | 13.05 | 25.36 | 34.78 | 34.17 | 7.94 |
| 42 | WT-042 | Hongxiaomai | landrace | 12.71 | 27.50 | 40.69 | 28.34 | 7.94 |
| 43 | WT-044 | Huoshaobairimai | landrace | 9.01 | 21.44 | 53.00 | 32.01 | 6.72 |
| 44 | WT-045 | Jizitou | landrace | 8.24 | 22.11 | 50.78 | 33.74 | 8.61 |
| 45 | WT-046 | Hongmangmai2 | landrace | 11.48 | 20.44 | 44.44 | 32.35 | 9.22 |
| 46 | WT-047 | Hongmangmai3 | landrace | 11.37 | 25.28 | 57.33 | 27.50 | 10.00 |
| 47 | WT-048 | Haiyanzhong | landrace | 11.88 | 20.31 | 40.08 | 41.16 | 9.58 |
| 48 | WT-049 | Cuodaosiguatou | landrace | 8.18 | 22.60 | 49.63 | 38.63 | 7.33 |
| 49 | WT-050 | Hongkeyangmai | landrace | 13.60 | 25.33 | 48.03 | 31.16 | 11.78 |
| 50 | WT-051 | ZM5574 | landrace | 12.57 | 23.39 | 49.86 | 31.71 | 8.22 |
| 51 | WT-052 | Guanyindumai | landrace | 9.40 | 22.69 | 46.83 | 38.44 | 8.92 |
| 52 | WT-053 | Daguchui | landrace | 8.10 | 21.72 | 47.61 | 31.90 | 11.61 |
| 53 | WT-054 | Dahuangpi | landrace | 11.34 | 19.87 | 35.93 | 30.75 | 7.22 |
| 54 | WT-055 | Hongmangxiaomai | landrace | 13.48 | 23.83 | 45.19 | 32.19 | 8.50 |
| 55 | WT-056 | Luziqing | landrace | 7.84 | 21.44 | 48.47 | 34.45 | 7.33 |
| 56 | WT-057 | Fang | landrace | 11.12 | 23.00 | 48.08 | 28.69 | 10.78 |
| 57 | WT-058 | Luopangtou | landrace | 7.84 | 21.67 | 44.83 | 32.24 | 6.00 |
| 58 | WT-059 | Heshangtou2 | landrace | 9.67 | 19.17 | 33.22 | 33.79 | 6.78 |
| 59 | WT-060 | Maopu | landrace | 7.57 | 22.44 | 52.78 | 34.17 | 7.17 |
| 60 | WT-061 | Dahongmang | landrace | 9.72 | 19.67 | 41.83 | 26.85 | 9.00 |
| 61 | WT-062 | Hongmai | landrace | 7.78 | 22.56 | 42.39 | 25.76 | 8.06 |
| 62 | WT-063 | Dahongpao | landrace | 11.49 | 23.22 | 47.83 | 29.91 | 9.83 |
| 63 | WT-064 | Hongmai2 | landrace | 10.93 | 21.78 | 51.53 | 39.65 | 6.56 |
| 64 | WT-065 | Honglumai | landrace | 8.24 | 22.11 | 55.42 | 30.30 | 8.33 |
| 65 | WT-066 | Hongkemai | landrace | 12.64 | 23.50 | 50.79 | 28.29 | 8.39 |
| 66 | WT-067 | Jinchaizhong | landrace | 13.58 | 23.07 | 57.93 | 26.38 | 9.60 |
| 67 | WT-068 | Jiangbeidalao | landrace | 7.78 | 20.75 | 40.46 | 26.94 | 5.92 |
| 68 | WT-069 | 13F10 | cultivar | 12.33 | 21.78 | 49.00 | 48.68 | 6.89 |
| 69 | WT-070 | W168 | cultivar | 8.76 | 21.40 | 48.20 | 43.86 | 5.53 |
| 70 | WT-071 | 64002/Yr5-1 | cultivar | 11.37 | 23.93 | 52.67 | 53.23 | 5.80 |
| 71 | WT-072 | 64002/Yr5-2 | cultivar | 11.05 | 23.80 | 52.33 | 54.46 | 5.07 |
| 72 | WT-073 | 64002/Yr5-3 | cultivar | 10.05 | 25.07 | 59.33 | 50.46 | 5.53 |
| 73 | WT-074 | 64002/Yr5-4 | cultivar | 10.11 | 24.73 | 55.56 | 54.92 | 4.20 |
| 74 | WT-075 | 64002/Yr5-5 | cultivar | 10.55 | 25.40 | 55.36 | 51.52 | 4.93 |
| 75 | WT-076 | 64002/Yr5-6 | cultivar | 9.55 | 22.40 | 49.07 | 45.19 | 6.07 |
| 76 | WT-077 | 64002/Yr5-7 | cultivar | 9.10 | 22.33 | 41.61 | 47.61 | 4.04 |
| 77 | WT-078 | 64002/Yr5-8 | cultivar | 10.51 | 23.60 | 47.87 | 49.29 | 5.07 |
| 78 | WT-079 | 64002/Yr5-9 | cultivar | 15.22 | 22.87 | 48.87 | 47.89 | 4.80 |
| 79 | WT-080 | 64002/Yr5-10 | cultivar | 10.39 | 24.13 | 55.27 | 51.79 | 5.07 |
| 80 | WT-081 | 64002/Yr5-11 | cultivar | 9.51 | 26.53 | 50.81 | 50.81 | 5.53 |
| 81 | WT-082 | 64002/Yr5-12 | cultivar | 9.60 | 20.20 | 44.56 | 48.23 | 3.87 |
| 82 | WT-083 | 64002/Yr5-13 | cultivar | 9.93 | 23.63 | 48.73 | 47.92 | 4.27 |
| 83 | WT-084 | 64002/Yr5-14 | cultivar | 10.31 | 24.00 | 48.32 | 44.83 | 5.00 |
| 84 | WT-085 | 64002/Yr5-15 | cultivar | 10.60 | 24.60 | 52.60 | 45.74 | 4.07 |
| 85 | WT-086 | 64002/Yr5-16 | cultivar | 10.77 | 25.27 | 51.60 | 47.90 | 5.27 |
| 86 | WT-087 | 64002/Yr5-17 | cultivar | 10.13 | 24.07 | 48.45 | 46.25 | 4.60 |
| 87 | WT-088 | 64002/Yr5-18 | cultivar | 10.00 | 24.13 | 53.30 | 48.89 | 5.00 |
| 88 | WT-089 | 7268/yingli | cultivar | 11.69 | 25.13 | 57.90 | 39.68 | 3.07 |
| 89 | WT-090 | W12R | cultivar | 7.79 | 20.40 | 50.23 | 36.91 | 4.00 |
| 90 | WT-091 | W12S | cultivar | 8.43 | 21.87 | 44.17 | 31.83 | 4.67 |
| 91 | WT-092 | W138 | cultivar | 9.17 | 23.27 | 41.80 | 41.09 | 6.27 |
| 92 | WT-093 | W139 | cultivar | 9.26 | 22.93 | 57.47 | 51.01 | 6.60 |
| 93 | WT-094 | W1718 | cultivar | 10.91 | 22.00 | 47.17 | 44.89 | 7.13 |
| 94 | WT-095 | W29 | cultivar | 10.45 | 22.07 | 45.07 | 44.38 | 7.00 |
| 95 | WT-096 | W465 | cultivar | 8.78 | 19.80 | 44.87 | 38.74 | 7.47 |
| 96 | WT-097 | W47 | cultivar | 9.10 | 22.60 | 54.30 | 41.83 | 4.87 |
| 97 | WT-098 | W5239 | cultivar | 9.87 | 23.80 | 45.83 | 45.00 | 5.73 |
| 98 | WT-099 | WJ36 | cultivar | 10.19 | 22.87 | 52.67 | 49.42 | 6.27 |
| 99 | WT-100 | Yr10 | cultivar | 9.11 | 19.00 | 33.93 | 35.46 | 8.33 |
| 100 | WT-101 | YR15 | cultivar | 9.15 | 20.60 | 35.03 | 35.27 | 10.13 |
| 101 | WT-102 | YR5 | cultivar | 9.49 | 20.00 | 34.87 | 40.45 | 10.07 |
| 102 | WT-103 | Chuanmai107 | cultivar | 8.53 | 22.28 | 55.06 | 46.21 | 4.83 |
| 103 | WT-104 | Chuanmai28 | cultivar | 9.84 | 22.47 | 49.10 | 45.68 | 4.20 |
| 104 | WT-105 | Chuanmai36 | cultivar | 9.89 | 21.03 | 43.88 | 44.33 | 8.47 |
| 105 | WT-106 | Chuanmai41 | cultivar | 10.79 | 25.53 | 64.79 | 43.51 | 5.47 |
| 106 | WT-107 | Chuanmai42 | cultivar | 11.57 | 21.94 | 53.69 | 51.66 | 5.78 |
| 107 | WT-108 | Chuanmai44 | cultivar | 7.73 | 22.00 | 40.13 | 36.67 | 4.93 |
| 108 | WT-109 | Chuanmai45 | cultivar | 9.95 | 20.93 | 46.53 | 56.84 | 5.87 |
| 109 | WT-110 | Chuanmai46 | cultivar | 10.97 | 22.87 | 62.00 | 51.68 | 5.47 |
| 110 | WT-111 | Chuanmai51 | cultivar | 8.84 | 21.80 | 46.63 | 41.01 | 4.63 |
| 111 | WT-112 | Chuanmai54 | cultivar | 9.02 | 18.87 | 39.89 | 39.22 | 6.67 |
| 112 | WT-113 | Chuanmai56 | cultivar | 10.49 | 20.67 | 45.20 | 45.69 | 7.73 |
| 113 | WT-114 | Chuanmai58 | cultivar | 12.77 | 22.30 | 55.80 | 46.41 | 6.20 |
| 114 | WT-115 | Chuannong12 | cultivar | 9.34 | 22.13 | 45.90 | 43.35 | 6.00 |
| 115 | WT-116 | Chuannong16 | cultivar | 8.54 | 20.00 | 39.30 | 41.03 | 8.07 |
| 116 | WT-117 | Chuannong17 | cultivar | 8.57 | 24.47 | 47.03 | 43.79 | 6.40 |
| 117 | WT-118 | Chuannong18 | cultivar | 9.17 | 22.80 | 49.60 | 44.53 | 5.87 |
| 118 | WT-119 | Chuannong19 | cultivar | 11.13 | 22.33 | 57.72 | 41.69 | 5.60 |
| 119 | WT-120 | Chuannong20 | cultivar | 9.11 | 20.60 | 33.83 | 46.42 | 5.87 |
| 120 | WT-121 | Chuannong21 | cultivar | 9.63 | 21.73 | 45.27 | 50.38 | 7.47 |
| 121 | WT-122 | Chuannong22 | cultivar | 9.91 | 23.20 | 47.17 | 40.44 | 5.80 |
| 122 | WT-123 | Chuannong23 | cultivar | 9.80 | 23.47 | 46.37 | 42.19 | 6.20 |
| 123 | WT-124 | Chuannong27 | cultivar | 9.91 | 21.87 | 46.05 | 49.22 | 6.07 |
| 124 | WT-125 | Chuanyu12 | cultivar | 9.37 | 22.07 | 51.30 | 40.94 | 4.13 |
| 125 | WT-126 | Chuanyu16 | cultivar | 9.86 | 21.90 | 33.83 | 50.46 | 4.60 |
| 126 | WT-127 | Chuanyu17 | cultivar | 9.01 | 24.47 | 57.87 | 44.10 | 7.47 |
| 127 | WT-128 | Chuanyu18 | cultivar | 10.16 | 23.67 | 47.23 | 47.48 | 6.20 |
| 128 | WT-129 | Chuanyu19 | cultivar | 10.04 | 23.87 | 45.43 | 46.76 | 6.07 |
| 129 | WT-130 | Chuanyu20 | cultivar | 11.27 | 24.67 | 59.37 | 49.78 | 5.00 |
| 130 | WT-131 | Chuanyu21 | cultivar | 9.87 | 23.33 | 51.23 | 49.03 | 6.00 |
| 131 | WT-132 | Chuanyu23 | cultivar | 8.55 | 20.47 | 45.40 | 57.47 | 6.07 |
| 132 | WT-133 | Chuanyu24 | cultivar | 10.69 | 23.80 | 58.33 | 52.13 | 6.67 |
| 133 | WT-134 | Chongzu104 | cultivar | 11.14 | 21.47 | 49.80 | 47.39 | 6.87 |
| 134 | WT-135 | 1522 | cultivar | 9.93 | 21.00 | 47.30 | 48.60 | 6.73 |
| 135 | WT-140 | Bailangmai | landrace | 11.10 | 22.07 | 37.37 | 38.54 | 7.13 |
| 136 | WT-141 | Bangdadongmai-1 | landrace | 10.51 | 23.33 | 30.50 | 30.25 | 7.07 |
| 137 | WT-142 | Bendihongmai | landrace | 12.11 | 21.73 | 47.73 | 53.48 | 7.57 |
| 138 | WT-143 | Zangchun6 | landrace | 9.87 | 22.40 | 30.96 | 26.61 | 7.80 |
| 139 | WT-144 | Zangdong6 | landrace | 7.88 | 21.93 | 32.95 | 27.73 | 9.60 |
| 140 | WT-145 | Fulanni | landrace | 8.07 | 21.50 | 42.88 | 34.12 | 5.87 |
| 141 | WT-146 | Jiachaanshaobailimai | landrace | 8.84 | 22.50 | 40.83 | 21.08 | 6.20 |
| 142 | WT-147 | Jiachabangdadongmai3 | landrace | 11.73 | 26.80 | 35.37 | 32.64 | 7.40 |
| 143 | WT-148 | Jiachabangdadongmai4 | landrace | 10.17 | 22.25 | 30.52 | 33.65 | 6.03 |
| 144 | WT-149 | Jiachabangdadongmai5 | landrace | 9.83 | 22.25 | 53.17 | 30.51 | 7.53 |
| 145 | WT-150 | Jianweihudouhuang | landrace | 9.17 | 23.13 | 60.28 | 36.07 | 14.07 |
| 146 | WT-151 | Luozhen1 | cultivar | 9.86 | 22.75 | 40.50 | 44.43 | 5.25 |
| 147 | WT-152 | Mian26 | cultivar | 10.49 | 21.00 | 50.96 | 47.07 | 4.40 |
| 148 | WT-153 | Mian33 | cultivar | 9.99 | 22.73 | 50.07 | 47.75 | 6.40 |
| 149 | WT-154 | Mian37 | cultivar | 8.88 | 21.00 | 39.27 | 43.25 | 5.67 |
| 150 | WT-155 | Mian38 | cultivar | 8.27 | 24.07 | 40.10 | 46.32 | 7.40 |
| 151 | WT-156 | Mian45 | cultivar | 10.18 | 24.00 | 46.10 | 51.25 | 6.50 |
| 152 | WT-157 | Mianyang1848 | cultivar | 10.07 | 24.47 | 52.17 | 43.46 | 6.40 |
| 153 | WT-158 | Mianyang20 | cultivar | 9.79 | 22.13 | 38.60 | 44.81 | 5.63 |
| 154 | WT-159 | Mianyang28 | cultivar | 10.86 | 21.53 | 44.20 | 41.94 | 3.37 |
| 155 | WT-160 | Nei2889 | cultivar | 8.93 | 20.40 | 45.03 | 45.17 | 4.80 |
| 156 | WT-161 | Rikaze2 | cultivar | 8.19 | 21.87 | 38.50 | 35.95 | 5.87 |
| 157 | WT-165 | Zhengzhou9023 | cultivar | 9.38 | 21.93 | 51.53 | 44.26 | 5.20 |
| 158 | WT-166 | Qubaichun | landrace | 11.57 | 19.56 | 39.22 | 59.08 | 7.33 |
| 159 | WT-167 | Chinese spring | landrace | 8.52 | 23.56 | 53.89 | 31.82 | 10.89 |
| 160 | WT-168 | SH018-2 | synthetic hexaploid | 11.06 | 16.67 | 28.31 | 49.04 | 4.72 |
| 161 | WT-169 | SH023-2 | synthetic hexaploid | 12.82 | 17.73 | 28.63 | 37.71 | 4.92 |
| 162 | WT-170 | SH031 | synthetic hexaploid | 12.91 | 21.80 | 26.27 | 45.04 | 7.43 |
| 163 | WT-171 | SH046-4 | synthetic hexaploid | 12.61 | 22.50 | 48.83 | 44.71 | 11.89 |
| 164 | WT-172 | SH052-3 | synthetic hexaploid | 11.16 | 17.00 | 23.19 | 49.77 | 8.94 |
| 165 | WT-173 | SH057-3 | synthetic hexaploid | 12.03 | 18.27 | 28.13 | 45.60 | 6.67 |
| 166 | WT-174 | SH060-2 | synthetic hexaploid | 10.91 | 16.25 | 27.58 | 45.35 | 8.25 |
| 167 | WT-175 | SH068-1 | synthetic hexaploid | 10.93 | 18.94 | 28.39 | 49.88 | 7.72 |
| 168 | WT-176 | SH076-2 | synthetic hexaploid | 11.14 | 18.94 | 45.36 | 36.67 | 9.39 |
| 169 | WT-177 | SH077-4 | synthetic hexaploid | 11.44 | 20.11 | 44.64 | 37.68 | 8.94 |
| 170 | WT-178 | SH103-2 | synthetic hexaploid | 11.40 | 22.61 | 47.31 | 32.94 | 7.67 |
| 171 | WT-179 | SH118-2 | synthetic hexaploid | 12.00 | 17.89 | 27.28 | 52.50 | 7.78 |
| 172 | WT-180 | SH120-4 | synthetic hexaploid | 12.28 | 18.00 | 38.67 | 47.26 | 6.72 |
| 173 | WT-181 | SH121-1 | synthetic hexaploid | 10.35 | 15.92 | 26.33 | 50.42 | 4.42 |
| 174 | WT-182 | SH122-1 | synthetic hexaploid | 11.43 | 18.22 | 27.08 | 54.15 | 10.94 |
| 175 | WT-183 | SH181-5 | synthetic hexaploid | 12.90 | 21.60 | 44.50 | 40.00 | 8.60 |
| 176 | WT-184 | SH182-2 | synthetic hexaploid | 14.10 | 22.28 | 55.61 | 32.58 | 9.11 |
| 177 | WT-185 | SH200-1 | synthetic hexaploid | 10.78 | 19.19 | 32.74 | 41.46 | 6.03 |
| 178 | WT-186 | SH204-3 | synthetic hexaploid | 11.81 | 20.53 | 37.97 | 42.18 | 8.64 |
| 179 | WT-187 | SH205-3 | synthetic hexaploid | 12.44 | 17.93 | 30.80 | 57.03 | 8.06 |
| 180 | WT-188 | SH222 | synthetic hexaploid | 11.14 | 17.80 | 35.33 | 52.09 | 5.06 |
| 181 | WT-189 | SH316-1 | synthetic hexaploid | 9.26 | 19.61 | 35.78 | 44.71 | 6.08 |
| 182 | WT-190 | SH352-1 | synthetic hexaploid | 21.24 | 18.73 | 25.47 | 49.62 | 7.07 |
| 183 | WT-191 | SH357-4 | synthetic hexaploid | 11.89 | 22.89 | 47.56 | 40.09 | 7.44 |
| 184 | WT-192 | SH365-3 | synthetic hexaploid | 11.31 | 21.50 | 35.79 | 46.66 | 7.00 |
| 185 | WT-194 | Wx-abd | cultivar | 8.71 | 22.20 | 47.40 | 35.41 | 5.47 |
| 186 | WT-200 | Wx-ABD | cultivar | 10.83 | 24.53 | 46.23 | 27.97 | 3.53 |
| 187 | WT-202 | Wx-aBD | cultivar | 8.35 | 21.47 | 49.17 | 34.59 | 4.13 |
| 188 | WT-205 | Wx-aBd | cultivar | 6.80 | 21.67 | 44.30 | 27.91 | 3.27 |
| 189 | WT-211 | Wx-ABd | cultivar | 8.47 | 22.27 | 48.87 | 33.94 | 4.73 |
| 190 | WT-214 | Wx-AbD | cultivar | 8.47 | 21.60 | 49.37 | 34.30 | 4.20 |
| 191 | WT-215 | Wx-abD | cultivar | 7.83 | 21.60 | 46.07 | 31.88 | 5.47 |
| 192 | WT-217 | Wx-Abd | cultivar | 8.37 | 21.67 | 49.70 | 35.58 | 3.80 |

AVE-SL: the average spike length, AVE-SN: the average spikelet number, AVE-KPS: the average kernels per spike, AVE-TKW: the average thousand kernel weight, AVE-SPN: the average spike number per plant.

**Supplementary Table 2** The information of the 276 wheat lines used for KASP assay

| **NO.** | **KASP code** | **Variety name** | **Zoning & Types** | **AVE-SL (cm)** | **AVE-SN (n)** | **AVE-KPS (n)** | **AVE-TKW** **(g)** |
| --- | --- | --- | --- | --- | --- | --- | --- |
| 1 | K1-01 | Honghuamai | Northwest spring wheat, landrace | 8.33 | 23.00 | 54.33 | 36.11 |
| 2 | K1-02 | Baimaizi | Northwest spring wheat, landrace | 9.17 | 22.33 | 64.00 | 31.29 |
| 3 | K1-03 | Chengduguangtou | Northwest spring wheat, landrace | 8.83 | 20.67 | 59.33 | 34.40 |
| 4 | K1-04 | Huanxiangguo | Northwest spring wheat, landrace | 7.00 | 22.50 | 68.67 | 33.00 |
| 5 | K1-05 | Chinese spring | Southwest winter wheat, landrace | 8.80 | 26.67 | 73.17 | 34.30 |
| 6 | K1-06 | ZM08817 | Southwest winter wheat, landrace | 10.47 | 24.50 | 62.33 | 22.76 |
| 7 | K1-07 | ZM08820 | Southwest winter wheat, landrace | 9.86 | 21.89 | 58.75 | 21.42 |
| 8 | K1-08 | ZM08824 | Southwest winter wheat, landrace | 10.67 | 22.72 | 60.58 | 25.47 |
| 9 | K1-09 | Guangtoumai | Southwest winter wheat, landrace | 9.62 | 20.28 | 44.56 | 30.59 |
| 10 | K1-10 | Wumanghonghuamai | Southwest winter wheat, landrace | 10.89 | 22.11 | 57.89 | 35.54 |
| 11 | K1-11 | Bendihuanghuamai | Southwest winter wheat, landrace | 13.44 | 25.44 | 59.72 | 23.52 |
| 12 | K1-12 | ZM11994 | Southwest winter wheat, landrace | 9.72 | 27.17 | 57.53 | 21.10 |
| 13 | K1-13 | Dayouzi | Southwest winter wheat, landrace | 7.70 | 25.44 | 44.86 | 25.37 |
| 14 | K1-14 | Wuhuatou | Southwest winter wheat, landrace | 12.15 | 26.22 | 52.58 | 29.33 |
| 15 | K1-15 | Hongyouzi | Southwest winter wheat, landrace | 9.57 | 25.00 | 59.25 | 30.26 |
| 16 | K1-16 | Hongchunmai | Southwest winter wheat, landrace | 10.02 | 20.39 | 32.64 | 36.09 |
| 17 | K1-17 | Hongmangmai | Southwest winter wheat, landrace | 11.38 | 22.00 | 35.14 | 33.18 |
| 18 | K1-18 | Heshangtou | Southwest winter wheat, landrace | 11.82 | 22.42 | 35.64 | 34.33 |
| 19 | K1-19 | Jinghuangmai | Southwest winter wheat, landrace | 11.87 | 22.39 | 39.61 | 36.53 |
| 20 | K1-20 | Dahongmai | Southwest winter wheat, landrace | 13.05 | 25.36 | 34.78 | 34.17 |
| 21 | K1-21 | Hongxiaomai | Southwest winter wheat, landrace | 12.71 | 27.50 | 40.69 | 28.34 |
| 22 | K1-22 | Huoshaobairimai | Southwest winter wheat, landrace | 9.01 | 21.44 | 53.00 | 32.01 |
| 23 | K1-23 | Jizitou | Southwest winter wheat, landrace | 8.24 | 22.11 | 50.78 | 33.74 |
| 24 | K1-24 | Hongmangmai2 | Southwest winter wheat, landrace | 11.48 | 20.44 | 44.44 | 32.35 |
| 25 | K1-25 | Hongmangmai3 | Southwest winter wheat, landrace | 11.37 | 25.28 | 57.33 | 27.50 |
| 26 | K1-26 | Haiyanzhong | Southwest winter wheat, landrace | 11.88 | 20.31 | 40.08 | 41.16 |
| 27 | K1-27 | Cuodaosiguatou | Southwest winter wheat, landrace | 8.18 | 22.60 | 49.63 | 38.63 |
| 28 | K1-28 | Hongkeyangmai | Southwest winter wheat, landrace | 13.60 | 25.33 | 48.03 | 31.16 |
| 29 | K1-29 | Guanyindumai | Southwest winter wheat, landrace | 9.40 | 22.69 | 46.83 | 38.44 |
| 30 | K1-30 | Daguchui | Southwest winter wheat, landrace | 8.10 | 21.72 | 47.61 | 31.90 |
| 31 | K1-31 | Dahuangpi | Southwest winter wheat, landrace | 11.34 | 19.87 | 35.93 | 30.75 |
| 32 | K1-32 | Hongmangxiaomai | Southwest winter wheat, landrace | 13.48 | 23.83 | 45.19 | 32.19 |
| 33 | K1-33 | Luziqing | Southwest winter wheat, landrace | 7.84 | 21.44 | 48.47 | 34.45 |
| 34 | K1-34 | Fang | Southwest winter wheat, landrace | 11.12 | 23.00 | 48.08 | 28.69 |
| 35 | K1-35 | Luopangtou | Southwest winter wheat, landrace | 7.84 | 21.67 | 44.83 | 32.24 |
| 36 | K1-36 | Heshangtou2 | Southwest winter wheat, landrace | 9.67 | 19.17 | 33.22 | 33.79 |
| 37 | K1-37 | Maopu | Southwest winter wheat, landrace | 7.57 | 22.44 | 52.78 | 34.17 |
| 38 | K1-38 | Dahongmang | Southwest winter wheat, landrace | 9.72 | 19.67 | 41.83 | 26.85 |
| 39 | K1-39 | Hongmai | Southwest winter wheat, landrace | 7.78 | 22.56 | 42.39 | 25.76 |
| 40 | K1-40 | Dahongpao | Southwest winter wheat, landrace | 11.49 | 23.22 | 47.83 | 29.91 |
| 41 | K1-41 | Hongmai2 | Southwest winter wheat, landrace | 10.93 | 21.78 | 51.53 | 39.65 |
| 42 | K1-42 | Honglumai | Southwest winter wheat, landrace | 8.24 | 22.11 | 55.42 | 30.30 |
| 43 | K1-43 | Hongkemai | Southwest winter wheat, landrace | 12.64 | 23.50 | 50.79 | 28.29 |
| 44 | K1-44 | 13F10 | Southwest winter wheat, culticar | 12.33 | 21.78 | 49.00 | 48.68 |
| 45 | K1-45 | Zinuo168 | Southwest winter wheat, culticar | 8.76 | 21.40 | 48.20 | 43.86 |
| 46 | K1-46 | Zhongkemai138 | Southwest winter wheat, culticar | 9.17 | 23.27 | 41.80 | 41.09 |
| 47 | K1-47 | Zhongkemai139 | Southwest winter wheat, culticar | 9.26 | 22.93 | 57.47 | 51.01 |
| 48 | K1-48 | Zhongkemai1718 | Southwest winter wheat, culticar | 10.91 | 22.00 | 47.17 | 44.89 |
| 49 | K1-49 | Zhongkemai29 | Southwest winter wheat, culticar | 10.45 | 22.07 | 45.07 | 44.38 |
| 50 | K1-50 | Zhongkemai47 | Southwest winter wheat, culticar | 9.10 | 22.60 | 54.30 | 41.83 |
| 51 | K1-51 | Zhongkemai5239 | Southwest winter wheat, culticar | 9.87 | 23.80 | 45.83 | 45.00 |
| 52 | K1-52 | Zhongkemai36 | Southwest winter wheat, culticar | 10.19 | 22.87 | 52.67 | 49.42 |
| 53 | K1-53 | Chuanmai107 | Southwest winter wheat, culticar | 8.53 | 22.28 | 55.06 | 46.21 |
| 54 | K1-54 | Chuanmai28 | Southwest winter wheat, culticar | 9.84 | 22.47 | 49.10 | 45.68 |
| 55 | K1-55 | Chuanmai36 | Southwest winter wheat, culticar | 9.89 | 21.03 | 43.88 | 44.33 |
| 56 | K1-56 | Chuanmai41 | Southwest winter wheat, culticar | 10.79 | 25.53 | 64.79 | 43.51 |
| 57 | K1-57 | Chuanmai42 | Southwest winter wheat, culticar | 11.57 | 21.94 | 53.69 | 51.66 |
| 58 | K1-58 | Chuanmai44 | Southwest winter wheat, culticar | 7.73 | 22.00 | 40.13 | 36.67 |
| 59 | K1-59 | Chuanmai46 | Southwest winter wheat, culticar | 10.97 | 22.87 | 62.00 | 51.68 |
| 60 | K1-60 | Chuanmai51 | Southwest winter wheat, culticar | 8.84 | 21.80 | 46.63 | 41.01 |
| 61 | K1-61 | Chuanmai54 | Southwest winter wheat, culticar | 9.02 | 18.87 | 39.89 | 39.22 |
| 62 | K1-62 | Chuanmai56 | Southwest winter wheat, culticar | 10.49 | 20.67 | 45.20 | 45.69 |
| 63 | K1-63 | Chuanmai58 | Southwest winter wheat, culticar | 12.77 | 22.30 | 55.80 | 46.41 |
| 64 | K1-64 | Chuannong12 | Southwest winter wheat, culticar | 9.34 | 22.13 | 45.90 | 43.35 |
| 65 | K1-65 | Chuannong16 | Southwest winter wheat, culticar | 8.54 | 20.00 | 39.30 | 41.03 |
| 66 | K1-66 | Chuannong17 | Southwest winter wheat, culticar | 8.57 | 24.47 | 47.03 | 43.79 |
| 67 | K1-67 | Chuannong18 | Southwest winter wheat, culticar | 9.17 | 22.80 | 49.60 | 44.53 |
| 68 | K1-68 | Chuannong19 | Southwest winter wheat, culticar | 11.13 | 22.33 | 57.72 | 41.69 |
| 69 | K1-69 | Chuannong20 | Southwest winter wheat, culticar | 9.11 | 20.60 | 33.83 | 46.42 |
| 70 | K1-70 | Chuannong21 | Southwest winter wheat, culticar | 9.63 | 21.73 | 45.27 | 50.38 |
| 71 | K1-71 | Chuannong22 | Southwest winter wheat, culticar | 9.91 | 23.20 | 47.17 | 40.44 |
| 72 | K1-72 | Chuannong23 | Southwest winter wheat, culticar | 9.80 | 23.47 | 46.37 | 42.19 |
| 73 | K1-73 | Chuannong27 | Southwest winter wheat, culticar | 9.91 | 21.87 | 46.05 | 49.22 |
| 74 | K1-74 | Chuanyu12 | Southwest winter wheat, culticar | 9.37 | 22.07 | 51.30 | 40.94 |
| 75 | K1-75 | Chuanyu16 | Southwest winter wheat, culticar | 9.86 | 21.90 | 33.83 | 50.46 |
| 76 | K1-76 | Chuanyu17 | Southwest winter wheat, culticar | 9.01 | 24.47 | 57.87 | 44.10 |
| 77 | K1-77 | Chuanyu18 | Southwest winter wheat, culticar | 10.16 | 23.67 | 47.23 | 47.48 |
| 78 | K1-78 | Chuanyu19 | Southwest winter wheat, culticar | 10.04 | 23.87 | 45.43 | 46.76 |
| 79 | K1-79 | Chuanyu20 | Southwest winter wheat, culticar | 11.27 | 24.67 | 59.37 | 49.78 |
| 80 | K1-80 | Chuanyu21 | Southwest winter wheat, culticar | 9.87 | 23.33 | 51.23 | 49.03 |
| 81 | K1-81 | Chuanyu23 | Southwest winter wheat, culticar | 8.55 | 20.47 | 45.40 | 57.47 |
| 82 | K1-82 | Chuanyu24 | Southwest winter wheat, culticar | 10.69 | 23.80 | 58.33 | 52.13 |
| 83 | K1-83 | Chuanchongzu104 | Southwest winter wheat, culticar | 11.14 | 21.47 | 49.80 | 47.39 |
| 84 | K1-84 | 1522 | Southwest winter wheat, culticar | 9.93 | 21.00 | 47.30 | 48.60 |
| 85 | K1-85 | Mian26 | Southwest winter wheat, culticar | 10.49 | 21.00 | 50.96 | 47.07 |
| 86 | K1-86 | Mian33 | Southwest winter wheat, culticar | 9.99 | 22.73 | 50.07 | 47.75 |
| 87 | K1-87 | Mian37 | Southwest winter wheat, culticar | 8.88 | 21.00 | 39.27 | 43.25 |
| 88 | K1-88 | Mian38 | Southwest winter wheat, culticar | 8.27 | 24.07 | 40.10 | 46.32 |
| 89 | K1-89 | Mian45 | Southwest winter wheat, culticar | 10.18 | 24.00 | 46.10 | 51.25 |
| 90 | K1-90 | Mianyang1848 | Southwest winter wheat, culticar | 10.07 | 24.47 | 52.17 | 43.46 |
| 91 | K1-91 | Mianyang20 | Southwest winter wheat, culticar | 9.79 | 22.13 | 38.60 | 44.81 |
| 92 | K1-92 | Mianyang28 | Southwest winter wheat, culticar | 10.86 | 21.53 | 44.20 | 41.94 |
| 93 | K2-01 | Xiaobaimai | Northern spring wheat, landrace | 12.33 | 26.33 | 59.00 | 32.88 |
| 94 | K2-02 | Dabaipi | Northern spring wheat, landrace | 13.33 | 25.67 | 47.50 | 27.14 |
| 95 | K2-03 | Xiaohongpi | Northern spring wheat, landrace | 11.75 | 24.50 | 46.00 | 23.30 |
| 96 | K2-04 | Dingxingzhai | Northern spring wheat, landrace | 11.50 | 21.50 | 50.00 | 30.52 |
| 97 | K2-05 | Honglidangnianlao | Northern spring wheat, landrace | 8.25 | 17.50 | 48.00 | 32.96 |
| 98 | K2-06 | Chunxiaomai | Northern spring wheat, landrace | 8.83 | 22.00 | 30.33 | 10.82 |
| 99 | K2-07 | Huoliaomai | Northern spring wheat, landrace | 13.67 | 25.67 | 48.00 | 18.31 |
| 100 | K2-08 | Dahongmai | Northern spring wheat, landrace | 13.00 | 20.50 | 50.50 | 57.31 |
| 101 | K2-09 | Shanxibaimai | Northern winter wheat, landrace | 10.00 | 23.67 | 48.00 | 12.70 |
| 102 | K2-10 | Niuzhijia | Northern winter wheat, landrace | 8.00 | 20.00 | 34.00 | 11.96 |
| 103 | K2-11 | Mahuaban | Northern winter wheat, landrace | 8.83 | 21.33 | 63.33 | 11.74 |
| 104 | K2-12 | Niuzhijia | Northern winter wheat, landrace | 9.33 | 23.00 | 69.00 | 20.22 |
| 105 | K2-13 | Hongjinmai | Northern winter wheat, landrace | 6.67 | 20.50 | 45.50 | 26.81 |
| 106 | K2-14 | Baiqimai | Northern winter wheat, landrace | 5.00 | 21.00 | 51.67 | 23.65 |
| 107 | K2-15 | Xiaokouhong | Northern winter wheat, landrace | 10.25 | 22.50 | 57.00 | 28.21 |
| 108 | K2-16 | Lanhuamai | Northern winter wheat, landrace | 6.83 | 22.33 | 68.33 | 23.16 |
| 109 | K2-17 | Daimanghongmai | Northern winter wheat, landrace | 11.67 | 22.00 | 45.33 | 24.54 |
| 110 | K2-18 | Zhuoludongmai | Northern winter wheat, landrace | 9.33 | 23.67 | 63.33 | 30.94 |
| 111 | K2-19 | Youmangbaifu | Northern winter wheat, landrace | 7.33 | 20.67 | 33.33 | 8.00 |
| 112 | K2-20 | Hongpidongmai | Northern winter wheat, landrace | 8.33 | 18.67 | 23.33 | 6.74 |
| 113 | K2-21 | Panshiwumang | Northern winter wheat, landrace | 11.50 | 23.00 | 27.50 | 10.56 |
| 114 | K2-22 | Baiqiumai | Northern winter wheat, landrace | 12.00 | 27.50 | 53.75 | 24.18 |
| 115 | K2-23 | Laomai | Northern winter wheat, landrace | 11.00 | 22.00 | 92.67 | 39.06 |
| 116 | K2-24 | Xiaobaimang | Northern winter wheat, landrace | 13.17 | 23.00 | 38.00 | 30.29 |
| 117 | K2-25 | Xianmai | Middle-lower Yangtze winter wheat, landrace | 10.33 | 20.67 | 63.00 | 31.31 |
| 118 | K2-26 | Jiangxizao | Middle-lower Yangtze winter wheat, landrace | 9.33 | 21.50 | 59.33 | 33.10 |
| 119 | K2-27 | Honghuazao | Middle-lower Yangtze winter wheat, landrace | 11.00 | 21.67 | 58.00 | 36.83 |
| 120 | K2-28 | Jiangdongmen | Middle-lower Yangtze winter wheat, landrace | 11.67 | 28.67 | 73.33 | 28.09 |
| 121 | K2-29 | Dahuangpi | Middle-lower Yangtze winter wheat, landrace | 9.83 | 20.67 | 49.33 | 33.42 |
| 122 | K2-30 | Congyanghongmai | Middle-lower Yangtze winter wheat, landrace | 11.00 | 23.33 | 63.00 | 37.35 |
| 123 | K2-31 | Zaowutian | Middle-lower Yangtze winter wheat, landrace | 10.00 | 22.67 | 55.67 | 29.87 |
| 124 | K2-32 | Liuzhutou | Middle-lower Yangtze winter wheat, landrace | 8.17 | 24.00 | 65.00 | 36.94 |
| 125 | K2-33 | Chanbuzhi | Middle-lower Yangtze winter wheat, landrace | 7.00 | 25.00 | 75.33 | 32.45 |
| 126 | K2-34 | Zhumaiyuanzitou | Middle-lower Yangtze winter wheat, landrace | 6.67 | 22.50 | 68.00 | 32.06 |
| 127 | K2-35 | Huangshuibai | Middle-lower Yangtze winter wheat, landrace | 10.00 | 27.50 | 72.50 | 33.99 |
| 128 | K2-36 | Baipu | Middle-lower Yangtze winter wheat, landrace | 8.75 | 24.00 | 65.00 | 40.64 |
| 129 | K2-37 | Zaoxiaomai | Middle-lower Yangtze winter wheat, landrace | 7.67 | 21.67 | 59.83 | 39.63 |
| 130 | K2-38 | Lanxizaoxiaomai | Middle-lower Yangtze winter wheat, landrace | 8.67 | 22.33 | 57.50 | 41.14 |
| 131 | K2-39 | Wangshuibai | Middle-lower Yangtze winter wheat, landrace | 11.33 | 21.00 | 52.33 | 43.91 |
| 132 | K2-40 | Chejianzi | Middle-lower Yangtze winter wheat, landrace | 8.33 | 18.33 | 40.50 | 32.29 |
| 133 | K2-41 | Heshangmai | Middle-lower Yangtze winter wheat, landrace | 13.00 | 24.67 | 73.33 | 32.81 |
| 134 | K2-42 | Nuomai | Middle-lower Yangtze winter wheat, landrace | 9.83 | 19.00 | 61.67 | 39.41 |
| 135 | K2-43 | Mangxiaomai | Middle-lower Yangtze winter wheat, landrace | 13.00 | 23.00 | 54.83 | 28.50 |
| 136 | K2-44 | Sankecun | Middle-lower Yangtze winter wheat, landrace | 13.00 | 19.00 | 37.33 | 46.55 |
| 137 | K2-45 | Paozimai | Middle-lower Yangtze winter wheat, landrace | 10.50 | 24.33 | 61.50 | 46.07 |
| 138 | K2-46 | Yangmai | Noutheast spring wheat, landrace | 18.00 | 26.00 | 62.00 | 18.51 |
| 139 | K2-47 | Dunhuachunmai | Noutheast spring wheat, landrace | 14.33 | 26.67 | 56.67 | 25.27 |
| 140 | K2-48 | Huoqiu | Noutheast spring wheat, landrace | 13 | 24.33 | 48.00 | 24.59 |
| 141 | K2-49 | Daqingmang | Noutheast spring wheat, landrace | 14.67 | 27.33 | 53.17 | 21.40 |
| 142 | K2-50 | Guangtou | Noutheast spring wheat, landrace | 15.33 | 23.33 | 51.50 | 26.70 |
| 143 | K2-51 | Xinkehan 9 | Noutheast spring wheat, landrace | 13.50 | 28.33 | 42.33 | 16.68 |
| 144 | K2-52 | Kefeng 3 | Noutheast spring wheat, landrace | 14.25 | 25.00 | 40.00 | 16.64 |
| 145 | K2-53 | Kelao 4 | Noutheast spring wheat, landrace | 10.50 | 22.67 | 44.17 | 35.39 |
| 146 | K2-54 | Xinshuguang 1 | Noutheast spring wheat, landrace | 10.67 | 18.67 | 42.00 | 45.36 |
| 147 | K2-55 | Dongnong 101 | Noutheast spring wheat, landrace | 10.00 | 21.33 | 40.60 | 22.85 |
| 148 | K2-56 | Xinshuguang 6 | Noutheast spring wheat, landrace | 13.17 | 24.50 | 62.33 | 41.07 |
| 149 | K2-57 | Jichun 1016 | Noutheast spring wheat, landrace | 14.00 | 25.00 | 69.50 | 33.81 |
| 150 | K2-58 | Baimangmai 2 | Huang-huai winter wheat, landrace | 9.83 | 25.00 | 49.00 | 15.22 |
| 151 | K2-59 | Huangguaxian | Huang-huai winter wheat, landrace | 10.00 | 24.00 | 57.00 | 26.61 |
| 152 | K2-60 | Banjiemang | Huang-huai winter wheat, landrace | 7.67 | 23.00 | 64.67 | 25.17 |
| 153 | K2-61 | Laolaixia | Huang-huai winter wheat, landrace | 6.83 | 20.33 | 45.00 | 24.68 |
| 154 | K2-62 | Louguding | Huang-huai winter wheat, landrace | 7.00 | 24.00 | 50.67 | 23.97 |
| 155 | K2-63 | Xishanbiansui | Huang-huai winter wheat, landrace | 7.67 | 22.00 | 53.00 | 30.54 |
| 156 | K2-64 | Honggoudou | Huang-huai winter wheat, landrace | 6.50 | 23.67 | 58.67 | 26.13 |
| 157 | K2-65 | Baihuomai | Huang-huai winter wheat, landrace | 8.50 | 21.33 | 46.00 | 23.38 |
| 158 | K2-66 | Sanyuehuang | Huang-huai winter wheat, landrace | 8.00 | 23.00 | 43.67 | 24.10 |
| 159 | K2-67 | Hongqiangchang | Huang-huai winter wheat, landrace | 8.50 | 23.00 | 38.00 | 26.13 |
| 160 | K2-68 | Youzimai | Huang-huai winter wheat, landrace | 5.67 | 21.33 | 59.00 | 31.92 |
| 161 | K2-69 | Pingyuan 50 | Huang-huai winter wheat, landrace | 8.00 | 23.67 | 67.33 | 35.21 |
| 162 | K2-70 | Baibiansui | Huang-huai winter wheat, landrace | 8.17 | 23.33 | 46.00 | 23.43 |
| 163 | K2-71 | Baiqimai | Huang-huai winter wheat, landrace | 5.50 | 17.67 | 33.67 | 20.96 |
| 164 | K2-72 | Baituzitou | Huang-huai winter wheat, landrace | 8.00 | 23.50 | 43.67 | 15.43 |
| 165 | K2-73 | Youmangsaogudan | Huang-huai winter wheat, landrace | 7.00 | 20.00 | 61.25 | 33.67 |
| 166 | K2-74 | Fuyanghong | Huang-huai winter wheat, landrace | 9.00 | 22.33 | 57.83 | 31.63 |
| 167 | K2-75 | Mazhamai | Huang-huai winter wheat, landrace | 7.50 | 23.00 | 63.17 | 26.53 |
| 168 | K2-76 | Qiangchangmai | Huang-huai winter wheat, landrace | 8.50 | 23.67 | 76.00 | 22.43 |
| 169 | K2-77 | Huomai | Huang-huai winter wheat, landrace | 9.33 | 22.00 | 54.33 | 20.73 |
| 170 | K2-78 | Meiqianwu | Huang-huai winter wheat, landrace | 11.67 | 22.67 | 57.33 | 28.67 |
| 171 | K2-79 | Jianmai | Huang-huai winter wheat, landrace | 10.00 | 26.67 | 51.33 | 19.93 |
| 172 | K2-80 | Sanyuehuang | Huang-huai winter wheat, landrace | 10.67 | 22.33 | 51.00 | 33.55 |
| 173 | K2-81 | Xiaofushou | Huang-huai winter wheat, landrace | 8.17 |  | 64.33 | 29.56 |
| 174 | K2-82 | Hongheshangtou | Huang-huai winter wheat, landrace | 9.67 | 24.33 | 66.67 | 34.37 |
| 175 | K2-83 | Dakoumai | Huang-huai winter wheat, landrace | 8.33 | 19.67 | 50.00 | 31.23 |
| 176 | K2-84 | Tumangmai | Huang-huai winter wheat, landrace | 6.00 | 22.00 | 62.00 | 34.32 |
| 177 | K2-85 | Baitiaoyu | Huang-huai winter wheat, landrace | 12.67 | 22.00 | 84.67 | 28.56 |
| 178 | K2-86 | Baimangmai | Huang-huai winter wheat, landrace | 9.50 | 23.50 | 58.50 | 19.74 |
| 179 | K2-87 | Dayuhua | Huang-huai winter wheat, landrace | 9.67 | 24.00 | 53.33 | 32.61 |
| 180 | K2-88 | Fumai | Huang-huai winter wheat, landrace | 10.00 | 24.00 | 67.17 | 28.21 |
| 181 | K2-89 | Laoqimai | Huang-huai winter wheat, landrace | 12.00 | 24.67 | 72.17 | 27.36 |
| 182 | K2-90 | Chushanbao | Huang-huai winter wheat, landrace | 6.33 | 18.33 | 46.83 | 34.81 |
| 183 | K2-91 | Zijiehong | Huang-huai winter wheat, landrace | 11.50 | 22.67 | 49.00 | 36.49 |
| 184 | K2-92 | Dalibanmang | Huang-huai winter wheat, landrace | 10.50 | 29.50 | 61.00 | 21.04 |
| 185 | K3-01 | Zhongmai 629 | Northern winter wheat, cultivar | 8.90 | 17.00 | 44.00 | 53.49 |
| 186 | K3-02 | Nongda 3615 | Northern winter wheat, cultivar | 8.30 | 20.00 | 50.00 | 35.61 |
| 187 | K3-03 | Jinmai 33 | Northern winter wheat, cultivar | 9.13 | 18.67 | 43.33 | 35.64 |
| 188 | K3-04 | Jinmai 31 | Northern winter wheat, cultivar | 8.17 | 20.00 | 36.83 | 37.60 |
| 189 | K3-05 | Jinmai 21 | Northern winter wheat, cultivar | 9.10 | 20.00 | 44.80 | 35.87 |
| 190 | K3-06 | Chang 6794 | Northern winter wheat, cultivar | 9.87 | 20.67 | 49.40 | 40.33 |
| 191 | K3-07 | Jinmai 98 | Northern winter wheat, cultivar | 8.33 | 18.67 | 33.83 | 31.83 |
| 192 | K3-08 | Jinmai 97 | Northern winter wheat, cultivar | 9.40 | 20.33 | 33.75 | 17.67 |
| 193 | K3-09 | Chang 6789 | Northern winter wheat, cultivar | 8.67 | 21.00 | 38.67 | 21.06 |
| 194 | K3-10 | Jinmai 91 | Northern winter wheat, cultivar | 8.67 | 17.67 | 50.50 | 34.59 |
| 195 | K3-11 | Yaomai 16 | Northern winter wheat, cultivar | 8.67 | 19.67 | 61.33 | 44.47 |
| 196 | K3-12 | Chang 6794 | Northern winter wheat, cultivar | 11.00 | 22.00 | 56.83 | 38.62 |
| 197 | K3-13 | Shimai 15 | Northern winter wheat, cultivar | 7.30 | 19.33 | 52.83 | 38.65 |
| 198 | K3-14 | Jinan 17 | Huang-huai winter wheat, cultivar | 10.30 | 20.50 | 49.50 | 27.82 |
| 199 | K3-15 | Jimai 0860299 | Huang-huai winter wheat, cultivar | 8.55 | 19.50 | 48.25 | 21.80 |
| 200 | K3-16 | Jimai 22 | Huang-huai winter wheat, cultivar | - | - | - | - |
| 201 | K3-17 | Yunhei 14207 | Huang-huai winter wheat, cultivar | 10.33 | 19.00 | 37.33 | 24.16 |
| 202 | K3-18 | Linhan 536 | Huang-huai winter wheat, cultivar | 9.20 | 21.00 | 46.50 | 32.39 |
| 203 | K3-19 | Yunfeng 198 | Huang-huai winter wheat, cultivar | 10.70 | 22.00 | 47.83 | 41.11 |
| 204 | K3-20 | Yunhan 20410 | Huang-huai winter wheat, cultivar | 10.07 | 19.67 | 59.00 | 28.51 |
| 205 | K3-21 | Linyou 2069 | Huang-huai winter wheat, cultivar | 8.07 | 18.00 | 43.83 | 34.42 |
| 206 | K3-22 | Kenong 199 | Huang-huai winter wheat, cultivar | 7.55 | 19.00 | 36.67 | 20.23 |
| 207 | K3-23 | Luyan 301 | Huang-huai winter wheat, cultivar | 7.07 | 18.00 | 39.50 | 48.40 |
| 208 | K3-24 | Qimin 6 | Huang-huai winter wheat, cultivar | 6.70 | 19.00 | 55.50 | 37.84 |
| 209 | K3-25 | Shannong 29 | Huang-huai winter wheat, cultivar | 7.45 | 19.00 | 40.25 | 35.81 |
| 210 | K3-26 | Shannong 24 | Huang-huai winter wheat, cultivar | 7.17 | 18.00 | 51.00 | 39.67 |
| 211 | K3-27 | Shannong 23 | Huang-huai winter wheat, cultivar | 9.67 | 20.00 | 59.83 | 45.56 |
| 212 | K3-28 | Shannong 20 | Huang-huai winter wheat, cultivar | 8.83 | 19.67 | 42.83 | 50.42 |
| 213 | K3-29 | Yan 999 | Huang-huai winter wheat, cultivar | 9.17 | 19.00 | 43.00 | 51.65 |
| 214 | K3-30 | Yannong 173 | Huang-huai winter wheat, cultivar | 9.27 | 18.33 | 39.67 | 44.70 |
| 215 | K3-31 | Jimai 22 | Huang-huai winter wheat, cultivar | 8.40 | 19.33 | 47.67 | 41.28 |
| 216 | K3-32 | Jimai 23 | Huang-huai winter wheat, cultivar | 8.15 | 20.50 | 41.50 | 24.21 |
| 217 | K3-33 | Liangxing 77 | Huang-huai winter wheat, cultivar | 7.43 | 18.33 | 41.00 | 45.30 |
| 218 | K3-34 | Zhengmai 583 | Middle-lower Yangtze winter wheat, cultivar | 8.90 | 22.33 | 47.33 | 33.06 |
| 219 | K3-35 | Zhoumai 27 | Middle-lower Yangtze winter wheat, cultivar | 9.87 | 22.33 | 42.17 | 26.85 |
| 220 | K3-36 | Zhoumai 22 | Middle-lower Yangtze winter wheat, cultivar | 8.83 | 18.00 | 43.33 | 39.65 |
| 221 | K3-37 | Zhoumai 18 | Middle-lower Yangtze winter wheat, cultivar | 8.90 | 20.33 | 49.67 | 46.07 |
| 222 | K3-38 | Zhoumai 16 | Middle-lower Yangtze winter wheat, cultivar | 8.97 | 22.67 | 42.20 | 44.04 |
| 223 | K3-39 | Zhengmai 7698 | Middle-lower Yangtze winter wheat, cultivar | 7.53 | 17.67 | 47.00 | 44.74 |
| 224 | K3-40 | Zhengmai 379 | Middle-lower Yangtze winter wheat, cultivar | 8.30 | 20.00 | 48.00 | 44.13 |
| 225 | K3-41 | Zhengmai 366 | Middle-lower Yangtze winter wheat, cultivar | 8.67 | 19.00 | 45.83 | 43.18 |
| 226 | K3-42 | Zhengmai 101 | Middle-lower Yangtze winter wheat, cultivar | 8.17 | 17.00 | 54.00 | 45.00 |
| 227 | K3-43 | Yumai 416 | Middle-lower Yangtze winter wheat, cultivar | 8.40 | 20.67 | 42.60 | 47.84 |
| 228 | K3-44 | Zheng 9023 | Middle-lower Yangtze winter wheat, cultivar | 9.23 | 18.00 | 43.00 | 48.51 |
| 229 | K3-45 | Jinghong 5 | Northern winter wheat, cultivar | 12.17 | 19.67 | 58.67 | 44.37 |
| 230 | K3-46 | Neimai 11 | Northern spring wheat, cultivar | 8.50 | 18.00 | 51.50 | 47.27 |
| 231 | K3-47 | Lianglaiyoubaipi | Northern spring wheat, cultivar | 12.50 | 25.67 | 49.33 | 29.99 |
| 232 | K3-48 | Bihongsui | Northern spring wheat, cultivar | 12.83 | 27.00 | 63.67 | 34.40 |
| 233 | K3-49 | Zhongyou 9507 | Northern winter wheat, cultivar | 9.00 | 23.00 | 59.00 | 51.01 |
| 234 | K3-50 | Jinmai 8 | Northern winter wheat, cultivar | 7.17 | 20.00 | 51.33 | 40.09 |
| 235 | K3-51 | Fengkang 2 | Northern winter wheat, cultivar | 9.33 | 23.00 | 58.67 | 44.81 |
| 236 | K3-52 | Changzhi 6406 | Northern winter wheat, cultivar | 8.00 | 20.33 | 46.00 | 38.15 |
| 237 | K3-53 | Beijing 8 | Northern winter wheat, cultivar | 8.00 | 18.67 | 49.67 | 38.84 |
| 238 | K3-54 | Yuandong 822 | Northern winter wheat, cultivar | 7.83 | 20.33 | 51.00 | 42.87 |
| 239 | K3-55 | Lvhan 328 | Northern winter wheat, cultivar | 8.50 | 19.33 | 69.67 | 35.31 |
| 240 | K3-56 | Yanan 11 | Northern winter wheat, cultivar | 11.00 | 19.67 | 36.83 | 32.49 |
| 241 | K3-57 | Nongda 183 | Northern winter wheat, cultivar | 8.17 | 20.67 | 35.83 | 23.33 |
| 242 | K3-58 | Nongda 311 | Northern winter wheat, cultivar | 10.00 | 22.67 | 36.50 | 26.29 |
| 243 | K3-59 | Nongda 139 | Northern winter wheat, cultivar | 8.00 | 21.33 | 39.67 | 31.81 |
| 244 | K3-60 | Mingxian 169 | Northern winter wheat, cultivar | 9.50 | 25.00 | 42.75 | 19.78 |
| 245 | K3-61 | Dongfanghong 3 | Northern winter wheat, cultivar | 13.00 | 24.00 | 59.67 | 34.75 |
| 246 | K3-62 | Huadong 6 | Middle-lower Yangtze winter wheat, cultivar | 9.67 | 21.00 | 48.83 | 34.06 |
| 247 | K3-63 | Liying 5 | Middle-lower Yangtze winter wheat, cultivar | 9.00 | 21.00 | 36.00 | 45.28 |
| 248 | K3-64 | Sumai 3 | Middle-lower Yangtze winter wheat, cultivar | 11.00 | 23.00 | 82.00 | 51.35 |
| 249 | K3-65 | Yangmai 158 | Middle-lower Yangtze winter wheat, cultivar | 10.33 | 23.50 | 64.50 | 53.65 |
| 250 | K3-66 | Enmai 4 | Middle-lower Yangtze winter wheat, cultivar | 9.33 | 24.00 | 67.50 | 48.36 |
| 251 | K3-67 | Emai 6 | Middle-lower Yangtze winter wheat, cultivar | 10.67 | 24.00 | 76.17 | 44.30 |
| 252 | K3-68 | Anhui 3 | Middle-lower Yangtze winter wheat, cultivar | 7.00 | 21.00 | 44.00 | 35.75 |
| 253 | K3-69 | Jinyang 60 | Huang-huai winter wheat, cultivar | 8.50 | 24.33 | 62.17 | 20.78 |
| 254 | K3-70 | Shite 14 | Huang-huai winter wheat, cultivar | 5.53 | 19.67 | 25.20 | 28.42 |
| 255 | K3-71 | Fuzhuang 30 | Huang-huai winter wheat, cultivar | 7.03 | 20.67 | 55.83 | 28.22 |
| 256 | K3-72 | Bima 1 | Huang-huai winter wheat, cultivar | 11.75 | 22.50 | 58.50 | 37.08 |
| 257 | K3-73 | Bima 4 | Huang-huai winter wheat, cultivar | 8.00 | 21.00 | 55.00 | 37.16 |
| 258 | K3-74 | Shijiazhuang 54 | Huang-huai winter wheat, cultivar | 7.43 | 21.67 | 49.83 | 31.74 |
| 259 | K3-75 | Pingyang 27 | Huang-huai winter wheat, cultivar | 7.70 | 20.00 | 41.67 | 46.42 |
| 260 | K3-76 | Fengchan 3 | Huang-huai winter wheat, cultivar | 9.57 | 21.67 | 53.33 | 41.02 |
| 261 | K3-77 | Taishan 1 | Huang-huai winter wheat, cultivar | 8.50 | 20.67 | 60.17 | 38.35 |
| 262 | K3-78 | Jinan 2 | Huang-huai winter wheat, cultivar | 8.93 | 22.00 | 51.17 | 46.29 |
| 263 | K3-79 | Youbao | Huang-huai winter wheat, cultivar | 6.33 | 17.00 | 36.00 | 28.38 |
| 264 | K3-80 | Bainong 3217 | Huang-huai winter wheat, cultivar | 10.67 | 24.33 | 69.00 | 34.27 |
| 265 | K3-81 | Yannong 15 | Huang-huai winter wheat, cultivar | 7.00 | 21.50 | 56.67 | 36.08 |
| 266 | K3-82 | Xinong 6028 | Huang-huai winter wheat, cultivar | 10.00 | 24.00 | 82.25 | 27.73 |
| 267 | K3-83 | Jimai 2 | Huang-huai winter wheat, cultivar | 9.33 | 19.33 | 53.17 | 40.65 |
| 268 | K3-84 | Neixiang 5 | Huang-huai winter wheat, cultivar | 11.17 | 24.00 | 57.83 | 53.67 |
| 269 | K3-85 | Zhengzhou 6 | Huang-huai winter wheat, cultivar | 13.00 | 21.33 | 53.00 | 49.20 |
| 270 | K3-86 | Xiannong 39 | Huang-huai winter wheat, cultivar | 10.33 | 21.67 | 44.50 | 48.50 |
| 271 | K3-87 | Jinan 17 | Huang-huai winter wheat, cultivar | 10.33 | 20.00 | 57.33 | 39.25 |
| 272 | K3-88 | Xiaoyan 6 | Huang-huai winter wheat, cultivar | 6.67 | 21.33 | 43.67 | 39.39 |
| 273 | K3-89 | Shannong 7859 | Huang-huai winter wheat, cultivar | 8.83 | 21.33 | 53.33 | 51.91 |
| 274 | K3-90 | Aifeng 3 | Huang-huai winter wheat, cultivar | 9.33 | 22.67 | 57.00 | 33.79 |
| 275 | K3-91 | Wenmai 6 | Huang-huai winter wheat, cultivar | 8.00 | 23.33 | 48.33 | 35.89 |
| 276 | K3-92 | Laizhou 953 | Huang-huai winter wheat, cultivar | 9.17 | 24.00 | 58.33 | 31.85 |

AVE-SL: the average spike length, AVE-SN: the average spikelet number, AVE-KPS: the average kernels per spike.

**Supplementary Table 3** The primers of KASP markers used to identify the haplotypes

| **Marker** | **SNP ID** | **Allele-specific forward primer (5′- 3′)** | **Reverse primer (5′- 3′)** |
| --- | --- | --- | --- |
| A010071 | IWB10891 | TCCAACCTGTTCTATTTGTGATCGA | GGTTGGAGCCAGTACCATTGCATTT |
|  |  | CCAACCTGTTCTATTTGTGATCGG |  |
| A010074 | IWB12818 | GACACAGGCAGTGCTAGCTCT | GGGATGGTTACCTGGGTAGGGTT |
|  |  | ACACAGGCAGTGCTAGCTCG |  |
| A010081 | IWB42599 | ACATGGTGAACATCGTCGGCTAT | GTGACGACTCTTACGAAGGGGTTAT |
|  |  | CATGGTGAACATCGTCGGCTAC |  |
| A010085 | IWB68523 | TACCCTTCGTGGCCACTATCTA | CAGGGTCAGATTGAGCATCTACGAA |
|  |  | CCCTTCGTGGCCACTATCTC |  |

**Supplementary Table 4** Phenotypic performance of the tested traits in different environments

| **Traits** | **Environments** | **Mean** | **Std.Deviation** | **ANOVA** | **Range** | **C.V.** | **h^2^** |
| --- | --- | --- | --- | --- | --- | --- | --- |
| SL | E1 | 8.52 | 1.30 | a | 6.0~12.0 | 15.30 | 0.69 |
| （cm） | E2 | 9.74 | 1.56 | b | 6.0~13.7 | 15.98 |  |
|  | E3 | 9.83 | 1.54 | b | 6.0~14.0 | 15.63 |  |
|  | E4 | 10.57 | 1.61 | c | 7.0~14.7 | 15.22 |  |
| SN | E1 | 21.31 | 2.37 | a | 14.7~27.0 | 11.12 | 0.76 |
| （n°） | E2 | 22.84 | 2.50 | b | 14.0~35.3 | 10.94 |  |
|  | E3 | 22.62 | 2.40 | b | 16.7~28.3 | 10.63 |  |
|  | E4 | 22.97 | 2.49 | b | 17.0~29.7 | 10.83 |  |
| KPS | E1 | 40.39 | 9.24 | a | 16.3~70.3 | 22.89 | 0.65 |
| （n°） | E2 | 47.73 | 10.29 | b | 19.0~68.3 | 21.56 |  |
|  | E3 | 42.83 | 10.00 | a | 17.3~71.5 | 23.36 |  |
|  | E4 | 46.08 | 11.57 | b | 16.3~70.3 | 25.12 |  |
| TKW | E1 | 43.17 | 9.40 | c | 20.61~65.33 | 21.77 | 0.82 |
| （g） | E2 | 41.08 | 8.54 | bc | 20.19~63.85 | 20.79 |  |
|  | E3 | 39.35 | 9.11 | ab | 14.92~59.46 | 23.14 |  |
|  | E4 | 37.96 | 9.68 | a | 18.01~60.69 | 25.51 |  |
| SNPP | E1 | 4.00 | 1.54 | a | 1.7~10.3 | 38.54 | 0.49 |
| （n°） | E2 | 8.38 | 2.75 | c | 3.0~20.7 | 32.79 |  |
|  | E3 | 6.94 | 2.85 | b | 2.3~26.0 | 41.07 |  |
|  | E4 | 8.23 | 2.88 | c | 2.7~19.0 | 34.96 |  |

In the environments column, E1, E2, E3, and E4 refer to average, 2015-2016 Shifang with low nitrogen treatment, 2015-2016 Shifang with high nitrogen treatment, 2015-2016 Shuangliu and 2014-2015 Shuangliu, respectively.

SL: spike length, SN: spikelet number, KPS: kernels per spike, TKW: thousand kernel weight, SNPP: spike number per plant, ANOVA: analysis of variance; C.V.: coefficient of variation; h^2^: broad sense heritability.

**Supplementary Table 5** ANOVA results of the tested traits in all the environments.

| Traits | Variation | Sum of Squares | df | Mean Square | F | *P*-value. | Phenotypic  variation explained by environment (%) |
| --- | --- | --- | --- | --- | --- | --- | --- |
| SL | Between Enviroments | 411.213 | 3 | 137.071 | 60.463 | .000 | 19.35 |
|  | Within Enviroments | 1713.883 | 756 | 2.267 |  |  |  |
|  | Total | 2125.097 | 759 |  |  |  |  |
| SN | Between Enviroments | 333.736 | 3 | 111.245 | 18.677 | .000 | 6.96 |
|  | Within Enviroments | 4461.307 | 749 | 5.956 |  |  |  |
|  | Total | 4795.043 | 752 |  |  |  |  |
| KPS | Between Enviroments | 6080.427 | 3 | 2026.809 | 19.092 | .000 | 7.18 |
|  | Within Enviroments | 78663.232 | 741 | 106.158 |  |  |  |
|  | Total | 84743.659 | 744 |  |  |  |  |
| TKW | Between Enviroments | 2888.845 | 3 | 962.948 | 11.398 | .000 | 4.32 |
|  | Within Enviroments | 63953.202 | 757 | 84.482 |  |  |  |
|  | Total | 66842.047 | 760 |  |  |  |  |
| SNPP | Between Enviroments | 2353.420 | 3 | 784.473 | 119.277 | .000 | 32.18 |
|  | Within Enviroments | 4958.975 | 754 | 6.577 |  |  |  |
|  | Total | 7312.395 | 757 |  |  |  |  |

ANOVA: analysis of variance, df: degree of freedom.

SL: spike length, SN: spikelet number, KPS: kernels per spike, TKW: thousand kernel weight, SNPP: spike number per plant.

**Supplementary Table 6** Person’s correlation coefficients between the different groups for tested traits.

| **Traits** | **TKW (ALL/ SH/ L/ C)** | **SNPP (ALL/ SH/ L/ C)** | **SL (ALL/ SH/ L/ C)** | **SN (ALL/ SH/ L/ C)** | **KPS (ALL/ SH/ L/ C)** |
| --- | --- | --- | --- | --- | --- |
| TKW-ALL | 1 | -0.419** | 0.189** | -0.280** | -0.164* |
| TKW-SH | 1 | -0.125 | 0.005 | -0.592** | -0.715** |
| TKW-L | 1 | -0.169 | 0.184 | -0.482** | -0.203 |
| TKW-C | 1 | 0.1 | 0.485** | 0.304** | 0.330** |
| SNPP-ALL | -0.419** | 1 | 0.202** | -0.006 | 0.021 |
| SNPP-SH | -0.125 | 1 | 0.101 | 0.419* | 0.391 |
| SNPP-L | -0.169 | 1 | 0.221* | 0.113 | 0.315** |
| SNPP-C | 0.1 | 1 | 0 | -0.286** | -0.315** |
| SL-ALL | 0.189** | 0.202** | 1 | -0.052 | -0.126 |
| SL-SH | 0.005 | 0.101 | 1 | 0.143 | -0.033 |
| SL-L | 0.184 | 0.221* | 1 | 0.15 | -0.022 |
| SL-C | 0.485** | 0 | 1 | 0.339** | 0.391** |
| SN-ALL | -0.280** | -0.006 | -0.052 | 1 | 0.589** |
| SN-SH | -0.592** | 0.419* | 0.143 | 1 | 0.740** |
| SN-L | -0.482** | 0.113 | 0.15 | 1 | 0.262* |
| SN-C | 0.304** | -0.286** | 0.339** | 1 | 0.583** |
| KPS-ALL | -0.164* | 0.021 | -0.126 | 0.589** | 1 |
| KPS-SH | -0.715** | 0.391 | -0.033 | 0.740** | 1 |
| KPS-L | -0.203 | 0.315** | -0.022 | 0.262* | 1 |
| KPS-C | 0.330** | -0.315** | 0.391** | 0.583** | 1 |

**Statistical significance level: *P*<0.01;

*Statistical significance level: *P*<0.05.

SL: spike length, SN: spikelet number, KPS: kernels per spike, TKW: thousand kernel weight, SNPP: spike number per plant. SH: Synthetic Hexaploid, L: Landrace, C: Cultivar.

**Supplementary Table 7** The chromosome regions of the highest 1% *F*_ST_ and *ROD* value between cultivar and landrace.

| **Chr.** | **Start (bp)** | **End (bp)** | ***F*_ST_** | ***ROD*** | **Chr.** | **Start (bp)** | **End (bp)** | ***F*_ST_** | ***ROD*** |
| --- | --- | --- | --- | --- | --- | --- | --- | --- | --- |
| 1A | *323,600,001 | 323,700,000 |  | 0.974 | 3D | **593,900,001 | 594,000,000 | 0.409 |  |
| 1A | *324,500,001 | 324,600,000 |  | 0.975 | 3D | **593,900,001 | 594,000,000 |  | 0.976 |
| 1A | 406,500,001 | 406,600,000 |  | 0.972 | 4A | 448,500,001 | 448,600,000 | 0.395 |  |
| 1A | 413,700,001 | 413,800,000 |  | 0.974 | 4A | 737,200,001 | 737,300,000 |  | 0.971 |
| 1A | 441,700,001 | 441,800,000 | 0.413 |  | 4D | 475,000,001 | 475,100,000 |  | 0.974 |
| 1A | 456,900,001 | 457,000,000 |  | 0.968 | 4D | 507,400,001 | 507,500,000 |  | 0.955 |
| 1A | 566,400,001 | 566,500,000 |  | 0.973 | 5A | 8,000,001 | 8,100,000 | 0.377 |  |
| 1A | *589,700,001 | 589,800,000 |  | 0.969 | 5A | 98,000,001 | 98,100,000 |  | 0.965 |
| 1A | *590,200,001 | 590,300,000 |  | 0.972 | 5A | 141,800,001 | 141,900,000 |  | 0.972 |
| 1B | *10,200,001 | 10,300,000 | 0.372 |  | 5A | 419,300,001 | 419,400,000 | 0.411 |  |
| 1B | *11,600,001 | 11,700,000 | 0.372 |  | 5A | 473,300,001 | 473,400,000 | 0.407 |  |
| 1B | 51,500,001 | 51,600,000 | 0.386 |  | 5A | *485,200,001 | 485,300,000 | 0.389 |  |
| 1B | 82,600,001 | 82,700,000 | 0.523 |  | 5A | *485,500,001 | 485,600,000 | **0.551** |  |
| 1B | **327,000,001 | 327,100,000 | 0.414 |  | 5A | 503,300,001 | 503,400,000 | 0.383 |  |
| 1B | **327,000,001 | 327,100,000 |  | 0.976 | 5A | 504,800,001 | 504,900,000 | 0.383 |  |
| 1B | 438,600,001 | 438,700,000 |  | 0.975 | 5A | 568,200,001 | 568,300,000 | 0.416 |  |
| 1B | 470,700,001 | 470,800,000 |  | 0.955 | 5A | *645,400,001 | 645,500,000 | 0.422 |  |
| 1B | *478,000,001 | 478,100,000 |  | 0.967 | 5A | *645,600,001 | 645,700,000 | 0.397 |  |
| 1B | *478,700,001 | 478,800,000 |  | 0.958 | 5A | **654,100,001 | 654,200,000 | 0.389 |  |
| 1B | 665,300,001 | 665,400,000 | 0.414 |  | 5A | **654,100,001 | 654,200,000 |  | 0.976 |
| 1D | 420,000,001 | 420,100,000 | 0.406 |  | 5A | 657,200,001 | 657,300,000 | 0.396 |  |
| 1D | 470,800,001 | 470,900,000 |  | 0.966 | 5A | *703,300,001 | 703,400,000 | 0.395 |  |
| 2A | 16,600,001 | 16,700,000 |  | 0.966 | 5A | *704,300,001 | 704,400,000 | 0.395 |  |
| 2A | 65,400,001 | 65,500,000 | 0.404 |  | 5A | *704,500,001 | 704,600,000 | 0.456 |  |
| 2A | ***74,100,001 | 74,200,000 | 0.406 |  | 5B | 137,200,001 | 137,300,000 | 0.489 |  |
| 2A | ***74,100,001 | 74,200,000 |  | 0.976 | 5B | 373,600,001 | 373,700,000 | 0.411 |  |
| 2A | ***75,100,001 | 75,200,000 | 0.405 |  | 5B | 471,000,001 | 471,100,000 |  | 0.974 |
| 2A | ***75,100,001 | 75,200,000 |  | 0.966 | 5B | *473,100,001 | 473,200,000 |  | 0.975 |
| 2A | *77,900,001 | 78,000,000 |  | 0.968 | 5B | *473,600,001 | 473,700,000 |  | 0.975 |
| 2A | *78,300,001 | 78,400,000 |  | 0.959 | 5B | *652,400,001 | 652,500,000 | 0.415 |  |
| 2A | 82,300,001 | 82,400,000 | 0.383 |  | 5B | *653,400,001 | 653,500,000 | 0.402 |  |
| 2A | 93,900,001 | 94,000,000 | 0.475 |  | 5B | 666,200,001 | 666,300,000 | 0.396 |  |
| 2A | 98,100,001 | 98,200,000 | 0.384 |  | 5D | 529,800,001 | 529,900,000 |  | 0.968 |
| 2A | 100,600,001 | 100,700,000 | 0.384 |  | 6A | 82,100,001 | 82,200,000 |  | 0.963 |
| 2A | 104,000,001 | 104,100,000 | 0.429 |  | 6A | 84,900,001 | 85,000,000 |  | 0.958 |
| 2A | 118,400,001 | 118,500,000 | 0.381 |  | 6A | 614,400,001 | 614,500,000 |  | 0.979 |
| 2A | 125,200,001 | 125,300,000 | 0.373 |  | 6B | **140,400,001 | 140,500,000 | 0.422 |  |
| 2A | 147,500,001 | 147,600,000 | 0.383 |  | 6B | **140,400,001 | 140,500,000 |  | 0.977 |
| 2A | 150,900,001 | 151,000,000 | 0.376 |  | 6B | 195,400,001 | 195,500,000 |  | 0.975 |
| 2A | 154,400,001 | 154,500,000 | 0.381 |  | 6B | 224,200,001 | 224,300,000 | 0.420 |  |
| 2A | *195,800,001 | 195,900,000 | 0.381 |  | 6B | 452,100,001 | 452,200,000 |  | 0.976 |
| 2A | *196,100,001 | 196,200,000 | 0.381 |  | 6B | **630,600,001 | 630,700,000 | 0.376 |  |
| 2A | *196,500,001 | 196,600,000 | 0.392 |  | 6B | **630,600,001 | 630,700,000 |  | 0.975 |
| 2A | 199,500,001 | 199,600,000 | 0.374 |  | 6B | ***661,300,001 | 661,400,000 | 0.380 |  |
| 2A | 238,700,001 | 238,800,000 | 0.398 |  | 6B | ***661,300,001 | 661,400,000 |  | 0.959 |
| 2A | 579,800,001 | 579,900,000 | 0.431 |  | 6B | ***661,500,001 | 661,600,000 | 0.389 |  |
| 2A | 608,600,001 | 608,700,000 |  | 0.967 | 6B | ***661,500,001 | 661,600,000 |  | 0.976 |
| 2A | 611,000,001 | 611,100,000 |  | 0.967 | 6B | 701,600,001 | 701,700,000 |  | 0.957 |
| 2A | 615,200,001 | 615,300,000 |  | 0.969 | 6B | 705,100,001 | 705,200,000 |  | 0.961 |
| 2A | 733,600,001 | 733,700,000 | 0.384 |  | 6D | 65,300,001 | 65,400,000 |  | 0.965 |
| 2A | 739,300,001 | 739,400,000 | 0.425 |  | 6D | 69,100,001 | 69,200,000 |  | 0.974 |
| 2B | 8,300,001 | 8,400,000 | 0.429 |  | 7A | **6,700,001 | 6,800,000 | 0.528 |  |
| 2B | 450,700,001 | 450,800,000 |  | 0.970 | 7A | **6,800,001 | 6,900,000 |  | 0.973 |
| 2B | 622,900,001 | 623,000,000 | 0.387 |  | 7A | **7,500,001 | 7,600,000 | 0.433 |  |
| 2B | 759,700,001 | 759,800,000 | 0.415 |  | 7A | 9,700,001 | 9,800,000 |  | 0.971 |
| 2B | 767,000,001 | 767,100,000 |  | 0.956 | 7A | 77,400,001 | 77,500,000 | 0.428 |  |
| 2D | 122,700,001 | 122,800,000 | 0.394 |  | 7A | 83,700,001 | 83,800,000 | 0.371 |  |
| 2D | 599,000,001 | 599,100,000 |  | 0.973 | 7A | *108,700,001 | 108,800,000 |  | 0.960 |
| 2D | 600,200,001 | 600,300,000 | 0.376 |  | 7A | *108,900,001 | 109,000,000 |  | 0.955 |
| 3A | 186,800,001 | 186,900,000 | 0.371 |  | 7A | 199,000,001 | 199,100,000 |  | 0.957 |
| 3A | 375,800,001 | 375,900,000 | 0.378 |  | 7A | 667,200,001 | 667,300,000 | 0.375 |  |
| 3A | 444,700,001 | 444,800,000 | 0.383 |  | 7B | 44,600,001 | 44,700,000 | 0.378 |  |
| 3A | 654,300,001 | 654,400,000 |  | 0.962 | 7B | 125,100,001 | 125,200,000 |  | **0.980** |
| 3A | **724,800,001 | 724,900,000 | 0.409 |  | 7B | 146,000,001 | 146,100,000 |  | 0.968 |
| 3A | **724,800,001 | 724,900,000 |  | 0.976 | 7B | 211,400,001 | 211,500,000 |  | 0.963 |
| 3A | 749,400,001 | 749,500,000 |  | 0.960 | 7B | 218,500,001 | 218,600,000 |  | 0.955 |
| 3B | 2,000,001 | 2,100,000 | 0.495 |  | 7B | 478,800,001 | 478,900,000 |  | 0.956 |
| 3B | *69,600,001 | 69,700,000 |  | 0.967 | 7B | 638,800,001 | 638,900,000 | 0.373 |  |
| 3B | *69,800,001 | 69,900,000 |  | 0.966 | 7B | 659,700,001 | 659,800,000 |  | 0.959 |
| 3B | 445,200,001 | 445,300,000 |  | 0.972 | 7D | 5,300,001 | 5,400,000 | 0.375 |  |
| 3B | 651,700,001 | 651,800,000 |  | 0.965 | 7D | 55,000,001 | 55,100,000 |  | 0.955 |
| 3B | 688,300,001 | 688,400,000 | 0.384 |  | 7D | *104,200,001 | 104,300,000 |  | 0.960 |
| 3D | 2,400,001 | 2,500,000 |  | 0.965 | 7D | *104,700,001 | 104,800,000 |  | 0.955 |

* Window clusters.

** Windows detected by two methods.

*** Window clusters detected by two methods.

**Supplementary Table 8** Overlapping regions between reported QTLs/markers and putative selection sweeps based on the LD.

| **Selective sweeps** | | | | | **Current and reported QTLs/ Markers** | | |
| --- | --- | --- | --- | --- | --- | --- | --- |
| **Chr.** | **Start (Mb)** | **End (Mp)** | ***F*_ST_** | ***ROD*** | **trait** | **Position (Mb)** | **References** |
| 1A | 323.60 | 323.70 |  | 0.974 | SN^1^ | 27.27_344.71 | Wang et al. 2011 |
| 1A | 324.50 | 324.60 |  | 0.975 | SN^1^ | 27.27_344.71 | Wang et al. 2011 |
| 1A | 441.70 | 441.80 | 0.413 |  | SNPP^1^ | 426.53_481.67 | Iehisa et al. 2014 |
| 1A | 456.90 | 457.00 |  | 0.968 | SNPP^1^ | 426.53_481.67 | Iehisa et al. 2014 |
| 1A | 566.40 | 566.50 |  | 0.973 | SN^2^ | 551.15_570.05 | Heidari et al. 2011 |
|  |  |  |  |  | KPS^1^ | 494.02_592.31 | Yuan et al. 2012 |
|  |  |  |  |  | KPS^2^ | 525.48_575.39 | Ge et al. 2012 |
| 1A | 589.70 | 589.80 |  | 0.969 | KPS^1^ | 494.02_592.31 | Yuan et al. 2012 |
| 1A | 590.20 | 590.30 |  | 0.972 | KPS^1^ | 494.02_592.31 | Yuan et al. 2012 |
| 1B | 51.50 | 51.60 | 0.386 |  | KPS^3^ | 52.35 | current research |
| 1B | 327.00 | 327.10 | 0.414 | 0.976 | SL^1^ | 213.68_668.76 | Boner et al. 2002 |
|  |  |  |  |  | SL^2^ | 306.07_394.64 | Li et al. 2002 |
| 1B | 438.60 | 438.70 |  | 0.975 | SL^1^ | 213.68_668.76 | Boner et al. 2002 |
|  |  |  |  |  | KPS^4^ | 408.80_465.47 | Jia et al. 2013 |
| 1B | 470.70 | 470.80 |  | 0.955 | SL^1^ | 213.68_668.76 | Boner et al. 2002 |
| 1B | 478.00 | 478.10 |  | 0.967 | SL^1^ | 213.68_668.76 | Boner et al. 2002 |
| 1B | 478.70 | 478.80 |  | 0.958 | SL^1^ | 213.68_668.76 | Boner et al. 2002 |
| 1B | 665.30 | 665.40 | 0.414 |  | SL^3^ | 555.77_664.52 | Cui et al. 2012 |
|  |  |  |  |  | SN^3^ | 624.36_686.76 | Ma et al. 2007 |
|  |  |  |  |  | TKW^1^ | 666.02 | Campbell et al. 1999 |
| 1D | 420.00 | 420.10 | 0.406 |  | SN^4^ | 420.07 | current research |
| 2A | 16.60 | 16.70 |  | 0.966 | TKW^2^ | 3.78_28.20 | Wang et al. 2009 |
| 2A | 75.10 | 75.20 | 0.405 | 0.966 | TaUBP24 | 75.14 | Liu et al., 2018 |
| 2A | 77.90 | 78.00 |  | 0.968 | SL^4^ | 77.94 | Liu et al. 2017 |
|  |  |  |  |  | SL^5^ | 77.94 | current research |
| 2A | 78.30 | 78.40 |  | 0.959 | SN^5^/KPS^5^ | 80.87 | Yao et al. 2009 |
|  |  |  |  |  | SL^6^ | 78.33/78.34 | current research |
| 2A | 82.30 | 82.40 | 0.383 |  | SN^5^/KPS^5^ | 80.87 | Yao et al. 2009 |
|  |  |  |  |  | SNPP^2^ | 80.87 | Wu et al. 2011 |
| 2A | 125.20 | 125.30 | 0.373 |  | SL^7^ | 123.21 | Yao et al. 2009 |
| 2A | 579.80 | 579.90 | 0.431 |  | TKW^3^ | 361.17_716.55 | McCartney et al. 2005 |
| 2A | 608.60 | 608.70 |  | 0.967 | TKW^3^ | 361.17_716.55 | McCartney et al. 2005 |
| 2A | 611.00 | 611.10 |  | 0.967 | KPS^6^ | 612.85 | Shi et al. 2017 |
|  |  |  |  |  | TKW^3^ | 361.17_716.55 | McCartney et al. 2005 |
| 2A | 615.20 | 615.30 |  | 0.969 | TKW^3^ | 361.17_716.55 | McCartney et al. 2005 |
| 2B | 8.30 | 8.40 | 0.429 |  | SN^6^ | 6.78_73.62 | Katkout et al. 2014 |
|  |  |  |  |  | KPS^7^/TKW^4^ | 8.07 | Azadi et al. 2015 |
| 2B | 622.90 | 623.00 | 0.387 |  | KPS^8^ | 557.37_670.60 | Yuan et al. 2012 |
|  |  |  |  |  | KPS^9^ | 621.47_800.85 | Yuan et al. 2012 |
|  |  |  |  |  | TKW^5^ | 621.47 | Habash et al. 2007 |
| 2B | 759.70 | 759.80 | 0.415 |  | SL^8^ | 757.22 | Mwadzingeni et al. 2017 |
|  |  |  |  |  | KPS^9^ | 621.47_800.85 | Yuan et al. 2012 |
|  |  |  |  |  | SNPP^3^ | 758.80 | Liu et al. 2017 |
| 2B | 767.00 | 767.10 |  | 0.956 | KPS^9^ | 621.47_800.85 | Yuan et al. 2012 |
| 2D | 122.70 | 122.80 | 0.394 |  | TKW^6^ | 120.03_383.00 | Yu et al. 2018/Ge et al. 2012 |
| 2D | 599.00 | 599.10 |  | 0.973 | SL^9^ | 480.17_611.21 | Fan et al. 2015 |
|  |  |  |  |  | SN^7^ | 593.74_647.43 | Ma et al. 2007 |
|  |  |  |  |  | KPS^10^ | 587.17_608.20 | Cui et al. 2014 |
|  |  |  |  |  | TKW^7^ | 580.01_608.63 | Cui et al. 2014 |
|  |  |  |  |  | TKW^8^ | 599.94 | Huang et al. 2006 |
| 2D | 600.20 | 600.30 | 0.376 |  | SL^9^ | 480.17_611.21 | Fan et al. 2015 |
|  |  |  |  |  | SN^7^ | 593.74_647.43 | Ma et al. 2007 |
|  |  |  |  |  | KPS^10^ | 587.17_608.20 | Cui et al. 2014 |
|  |  |  |  |  | TKW^7^ | 580.01_608.63 | Cui et al. 2014 |
|  |  |  |  |  | TKW^8^ | 599.94 | Huang et al. 2006 |
| 3A | 654.30 | 654.40 |  | 0.962 | TKW^9^ | 625.79_690.74 | Zhang et al. 2014 |
| 3A | 749.40 | 749.50 |  | 0.960 | TaTGW6-A1 | 749.41 | Hanif et al. 2016 |
|  |  |  |  |  | TKW^10^ | 750.32 | Boner et al. 2002 |
| 3B | 2.00 | 2.10 | 0.495 |  | SL^10^ | 0.22 | current research |
| 3B | 651.70 | 651.80 |  | 0.965 | SNPP^4^ | 630_736.75 | Naruoka et al. 2011 |
| 3B | 688.30 | 688.40 | 0.384 |  | SNPP^4^ | 630_736.75 | Naruoka et al. 2011 |
| 3D | 2.40 | 2.50 |  | 0.965 | TKW^11^ | 2.75_3.23 | Zhang et al. 2013 |
| 4A | 448.50 | 448.60 | 0.395 |  | TKW^12^ | 308.86_580.66 | Wang et al. 2009 |
| 4A | 737.20 | 737.30 |  | 0.971 | KPS^11^ | 737.43 | Mwadzingeni et al. 2017 |
| 5A | 8.00 | 8.10 | 0.377 |  | SNPP^5^ | 9.32 | current research |
| 5A | 141.80 | 141.90 |  | 0.972 | SL^11^ | 105.43_299.49 | Ma et al. 2007 |
|  |  |  |  |  | TKW^13^ | 104.23_299.49 | Li et al. 2015 |
| 5A | 419.30 | 419.40 | 0.411 |  | SL^12^ | 299.49_659.13 | Chu et al. 2008 |
| 5A | 473.30 | 473.40 | 0.407 |  | SL^12^ | 299.49_659.13 | Chu et al. 2008 |
|  |  |  |  |  | KPS^12^ | 471.71 | Liu et al. 2006 |
|  |  |  |  |  | TKW^14^ | 471.71 | Wang et al. 2009 |
|  |  |  |  |  | TKW^15^ | 471.71_503.06 | Liu et al. 2014 |
| 5A | 485.20 | 485.30 | 0.389 |  | SL^12^ | 299.49_659.13 | Chu et al. 2008 |
|  |  |  |  |  | SL^13^ | 485.83_501.41 | Yu et al. 2014 |
|  |  |  |  |  | SN^8^/TKW^16^ | 483.14_655.99 | Kato et al. 2000 |
|  |  |  |  |  | TKW^15^ | 471.71_503.06 | Liu et al. 2014 |
| 5A | 485.50 | 485.60 | **0.551** |  | SL^12^ | 299.49_659.13 | Chu et al. 2008 |
|  |  |  |  |  | SL^13^ | 485.83_501.41 | Yu et al. 2014 |
|  |  |  |  |  | SN^8^/TKW^16^ | 483.14_655.99 | Kato et al. 2000 |
|  |  |  |  |  | TKW^15^ | 471.71_503.06 | Liu et al. 2014 |
| 5A | 503.30 | 503.40 | 0.383 |  | SL^12^ | 299.49_659.13 | Chu et al. 2008 |
|  |  |  |  |  | SN^8^/TKW^16^ | 483.14_655.99 | Kato et al. 2000 |
|  |  |  |  |  | TKW^15^ | 471.71_503.06 | Liu et al. 2014 |
|  |  |  |  |  | TKW^17^ | 492.89_523.76 | Tang et al. 2011 |
| 5A | 504.80 | 504.90 | 0.383 |  | SL^12^ | 299.49_659.13 | Chu et al. 2008 |
|  |  |  |  |  | SN^8^/TKW^16^ | 483.14_655.99 | Kato et al. 2000 |
|  |  |  |  |  | TKW^17^ | 492.89_523.76 | Tang et al. 2011 |
| 5A | 568.20 | 568.30 | 0.416 |  | SL^12^ | 299.49_659.13 | Chu et al. 2008 |
|  |  |  |  |  | SN^8^/TKW^16^ | 483.14_655.99 | Kato et al. 2000 |
| 5A | 645.40 | 645.50 | 0.422 |  | SL^12^ | 299.49_659.13 | Chu et al. 2008 |
|  |  |  |  |  | SL^13^ | 642.56_659.33 | Luo et al. 2016 |
|  |  |  |  |  | SN^8^/TKW^16^ | 483.14_655.99 | Kato et al. 2000 |
| 5A | 645.60 | 645.70 | 0.397 |  | SL^12^ | 299.49_659.13 | Chu et al. 2008 |
|  |  |  |  |  | SL^13^ | 642.56_659.33 | Luo et al. 2016 |
|  |  |  |  |  | SN^8^/TKW^16^ | 483.14_655.99 | Kato et al. 2000 |
| 5A | 654.10 | 654.20 | 0.389 | 0.976 | SL^12^ | 299.49_659.13 | Chu et al. 2008 |
|  |  |  |  |  | SL^13^ | 642.56_659.33 | Luo et al. 2016 |
|  |  |  |  |  | SN^8^/TKW^16^ | 483.14_655.99 | Kato et al. 2000 |
|  |  |  |  |  | SNPP^6^ | 654.17_656 | Kato et al. 2000 |
| 5A | 657.20 | 657.30 | 0.396 |  | SL^12^ | 299.49_659.13 | Chu et al. 2008 |
|  |  |  |  |  | SL^13^ | 642.56_659.33 | Luo et al. 2016 |
|  |  |  |  |  | TKW^16^ | 483.14_655.99 | Kato et al. 2000 |
| 5A | 703.30 | 703.40 | 0.395 |  | KPS^13^ | 702.50 | Li et al. 2015 |
| 5A | 704.30 | 704.40 | 0.395 |  | TKW^18^ | 705.45 | Yu et al. 2014 |
| 5A | 704.50 | 704.60 | 0.456 |  | TKW^18^ | 705.45 | Yu et al. 2014 |
| 5B | 137.20 | 137.30 | 0.489 |  | TKW^19^ | 66.61_232.26 | Tang et al. 2011 |
| 5B | 373.60 | 373.70 | 0.411 |  | TKW^20^ | 48.85_402.79 | Tang et al. 2011 |
| 5B | 471.00 | 471.10 |  | 0.974 | KPS^14^ | 418.81_477.51 | Tang et al. 2011 |
| 5B | 473.10 | 473.20 |  | 0.975 | KPS^14^ | 418.81_477.51 | Tang et al. 2011 |
| 5B | 473.60 | 473.70 |  | 0.975 | KPS^14^ | 418.81_477.51 | Tang et al. 2011 |
| 5B | 652.40 | 652.50 | 0.415 |  | SL^14^ | 504.30_712.85 | Xu et al. 2012 |
| 5B | 653.40 | 653.50 | 0.402 |  | SL^14^ | 504.30_712.85 | Xu et al. 2012 |
| 5B | 666.20 | 666.30 | 0.396 |  | SL^14^ | 504.30_712.85 | Xu et al. 2012 |
| 6A | 82.10 | 82.20 |  | 0.963 | SL^15^ | 81.12_545.13 | Cui et al. 2011 |
|  |  |  |  |  | TKW^21^ | 80.70 | current research |
| 6A | 84.90 | 85.00 |  | 0.958 | SL^15^ | 81.12_545.13 | Cui et al. 2011 |
| 6A | 614.40 | 614.50 |  | 0.979 | SL^16^ | 292.28_613.63 | Heidari et al. 2011 |
|  |  |  |  |  | KPS^15^ | 614.80 | current research |
| 6B | 195.40 | 195.50 |  | 0.975 | TKW^22^ | 172.73_227.28 | Wang et al. 2011 |
| 6B | 224.20 | 224.30 | 0.420 |  | TKW^22^ | 172.73_227.28 | Wang et al. 2011 |
|  |  |  |  |  | TKW^23^ | 199.08_234.56 | Boner et al. 2002 |
| 6B | 661.30 | 661.40 | 0.380 | 0.959 | SL^17^ | 656.19_676.71 | Wang et al. 2011 |
|  |  |  |  |  | TKW^24^ | 656.19_674.84 | Mir et al. 2012 |
| 6B | 661.50 | 661.60 | 0.389 | 0.976 | SL^17^ | 656.19_676.71 | Wang *et al.* 2011 |
|  |  |  |  |  | TKW^24^ | 656.19_674.84 | Mir et al. 2012 |
| 6B | 705.10 | 705.20 |  | 0.961 | SL^18^ | 705.16 | Sun et al. 2017 |
|  |  |  |  |  | SL^19^ | 705.21 | Liu et al. 2017 |
|  |  |  |  |  | TKW^25^ | 704.18/705.16 | Li et al. 2015 |
| 6D | 65.30 | 65.40 |  | 0.965 | TKW^26^ | 64.77 | Liu *et al.* 2017 |
| 7A | 6.80 | 6.90 |  | 0.973 | SN^9^ | 6.80_89.97 | Ma et al. 2007 |
| 7A | 7.50 | 7.60 | 0.433 |  | SN^9^ | 6.80_89.97 | Ma et al. 2007 |
|  |  |  |  |  | SN^10^ | 8.37 | current research |
| 7A | 9.70 | 9.80 |  | 0.971 | SN^9^ | 6.80_89.97 | Ma et al. 2007 |
|  |  |  |  |  | SN^10^ | 8.37 | current research |
| 7A | 77.40 | 77.50 | 0.428 |  | SN^9^ | 6.80_89.97 | Ma et al. 2007 |
| 7A | 83.70 | 83.80 | 0.371 |  | SN^9^ | 6.80_89.97 | Ma et al. 2007 |
| 7A | 108.70 | 108.80 |  | 0.960 | KPS^15^ | 110.65 | Mwadzingeni et al. 2017 |
| 7A | 108.90 | 109.00 |  | 0.955 | KPS^15^ | 110.65 | Mwadzingeni et al. 2017 |
| 7A | 199.00 | 199.10 |  | 0.957 | SN^11^ | 199.01 | current research |
| 7B | 211.40 | 211.50 |  | 0.963 | KPS^16^ | 176.03_452.49 | Yu et al. 2018 |
| 7B | 218.50 | 218.60 |  | 0.955 | KPS^16^ | 176.03_452.49 | Yu et al. 2018 |
|  |  |  |  |  | TKW^27^ | 220.31 | Xu et al. 2017/Cui et al. 2014 |
|  |  |  |  |  | SL^20^ | 218.56 | current research |
| 7B | 478.80 | 478.90 |  | 0.956 | KPS^17^ | 452.10_723.01 | Cui et al. 2014 |
|  |  |  |  |  | KPS^18^ | 475.65_546.86 | Yuan et al. 2012 |
| 7B | 638.80 | 638.90 | 0.373 |  | KPS^17^ | 452.10_723.01 | Cui et al. 2014 |
|  |  |  |  |  | SNPP^6^ | 637.92 | Wu et al. 2011 |
| 7B | 659.70 | 659.80 |  | 0.959 | SL^21^ | 642.77_670.51 | Azadi et al. 2015 |
|  |  |  |  |  | SL^22^/SN^12^ | 651.50_690.87 | Wang et al. 2011 |
|  |  |  |  |  | KPS^17^ | 452.10_723.01 | Cui et al. 2014 |
| 7D | 104.20 | 104.30 |  | 0.960 | SNPP^7^ | 104.75 | current research |
| 7D | 104.70 | 104.80 |  | 0.955 | SNPP^7^ | 104.75 | current research |

SL: spike length, SN: spikelet number, KPS: kernels per spike, TKW: thousand kernel weight, SNPP: spike number per plant.

**Supplementary Table 9** Significant SNPs associated with spike length detected by GWAS.

| **SNP** | **Chr.** | **Pos. (Mb)** | **-Log_10_*P*** | **MAF** | ***R*^2^** | **Env.** | **Ref.** |
| --- | --- | --- | --- | --- | --- | --- | --- |
| BobWhite_c6664_644 | 1A | 574.94 | 3.10 | 0.469 | 0.051 | E3 |  |
| BS00093078_51 | 1B | 1.20 * | 4.35 | 0.221 | 0.081 | E4 |  |
| Excalibur_c21898_1423 | 1B | 1.42 * | 4.32 | 0.173 | 0.080 | E4 |  |
| RAC875_c74_204 | 2A | 24.06 | 4.39/3.87 | 0.359/0.364 | 0.077/0.071 | E3/E4 |  |
| BS00003613_51 | 2A | 41.64 * | 3.78 | 0.340 | 0.068 | E1 |  |
| IACX5750 | 2A | 41.64 * | 3.78 | 0.340 | 0.068 | E1 |  |
| Kukri_c4710_991 | 2A | 58.01 | 3.27 | 0.271 | 0.058 | E4 |  |
| BobWhite_c17476_149 | 2A | 77.94 * | 3.80 | 0.204 | 0.068 | E1 | Liu et al*.* 2017 |
| Kukri_rep_c71876_521 | 2A | 78.33 * | 3.03 | 0.194 | 0.052 | E1 | Selective region |
| wsnp_Ex_c15325_23565935 | 2A | 78.33 * | 3.76 | 0.202 | 0.067 | E1 |  |
| Kukri_c7818_2581 | 2A | 78.34 * | 3.80 | 0.204 | 0.068 | E1 |  |
| wsnp_Ex_c15325_23564654 | 2A | 78.34 * | 3.80 | 0.204 | 0.068 | E1 |  |
| BS00021712_51 | 2A | 770.36 | 3.61 | 0.117 | 0.065 | E4 |  |
| CAP7_c3814_303 | 2B | 233.27 * | 3.09/3.42 | 0.304/0.309 | 0.051/0.061 | E3/E4 | Kumar et al. 2007 |
| BS00066546_51 | 2B | 233.27 * | 3.09/3.42 | 0.304/0.309 | 0.051/0.061 | E3/E4 |  |
| CAP11_c2947_204 | 2B | 233.27 * | 3.13/3/38 | 0.301/0.306 | 0.052/0.060 | E3/E4 |  |
| Tdurum_contig52063_1077 | 2B | 233.27 * | 3.09/3.42 | 0.304/0.309 | 0.051/0.061 | E3/E4 |  |
| Excalibur_c58566_284 | 2B | 233.55 * | 3.25 | 0.324 | 0.057 | E4 |  |
| Kukri_c5711_112 | 2B | 234.64 | 3.37 | 0.322 | 0.060 | E4 |  |
| RAC875_c46788_60 | 2B | 463.28 | 3.36 | 0.276 | 0.059 | E2 |  |
| Kukri_c44769_750 | 2B | 742.47 | 3.61 | 0.204 | 0.061 | E3 |  |
| Excalibur_c17745_493 | 2B | 743.69 | 3.74 | 0.351 | 0.067 | E1 |  |
| Tdurum_contig67879_504 | 2B | 793.58 | 3.15 | 0.114 | 0.055 | E4 |  |
| BobWhite_c30520_323 | 2D | 5.99 | 3.47 | 0.298 | 0.061 | E1 |  |
| Kukri_c4949_201 | 2D | 16.49 | 3.15 | 0.296 | 0.055 | E1 |  |
| Excalibur_c44325_638 | 2D | 608.20 * | 3.10 | 0.220 | 0.051 | E3 | Fan et al. 2015 |
| RAC875_c26979_266 | 2D | 608.57 * | 3.76/3.45 | 0.212/0.210 | 0.064/0.061 | E3/E4 |  |
| RAC875_c14766_1063 | 2D | 608.77 * | 3.87 | 0.107 | 0.067 | E3 |  |
| tplb0021a02_817 | 2D | 608.92 * | 3.16 | 0.209 | 0.052 | E3 |  |
| RAC875_c7727_1547 | 3B | 0.22 | 3.38 | 0.395 | 0.059 | E2 | Selective region |
| RFL_Contig29_1062 | 3B | 40.91 | 3.54 | 0.353 | 0.063 | E1 |  |
| wsnp_Ex_c41074_47988141 | 4A | 617.95 * | 3.65 | 0.441 | 0.066 | E4 | Wang et al. 2011 |
| wsnp_Ex_c41074_47987998 | 4A | 617.95 * | 3.52 | 0.431 | 0.063 | E4 |  |
| wsnp_Ra_c11532_18688426 | 5A | 666.31 | 3.36 | 0.337 | 0.059 | E2 | Ma et al. 2012 |
| RAC875_c49875_405 | 6A | 60.17 * | 3.91 | 0.161 | 0.071 | E2 |  |
| RAC875_c60695_112 | 6A | 60.41 * | 3.53/3.38/ 3.92/4.98 | 0.191/0.192/0.186/0.184 | 0.063/0.059/0.068/0.095 | E1/E2/E3/E4 |  |
| BS00079942_51 | 6A | 61.02 * | 3.26 | 0.137 | 0.057 | E2 |  |
| wsnp_CAP7_c1839_908093 | 6A | 61.21 * | 3.42 | 0.153 | 0.060 | E2 |  |
| wsnp_CAP7_c1839_908011 | 6A | 61.21 * | 3.42 | 0.153 | 0.060 | E2 |  |
| wsnp_CAP7_c1839_907899 | 6A | 61.21 * | 3.42 | 0.153 | 0.060 | E2 |  |
| Kukri_c45898_147 | 6A | 61.39 * | 3.42 | 0.153 | 0.060 | E2 |  |
| Tdurum_contig30341_88 | 6B | 578.51 | 3.33 | 0.153 | 0.058 | E2 |  |
| JD_c20537_742 | 6B | 707.18 * | 3.22 | 0.229 | 0.056 | E4 | Mwadzingeni et al. 2017 |
| Jagger_c1231_85 | 6B | 708.02 * | 3.18 | 0.215 | 0.052 | E3 |  |
| tplb0046e21_618 | 6B | 708.03 * | 4.00 | 0.126 | 0.069 | E3 |  |
| IACX1734 | 7A | 202.72 * | 3.16 | 0.335 | 0.055 | E1 |  |
| RAC875_c20657_2535 | 7A | 202.72 * | 3.16 | 0.335 | 0.055 | E1 |  |
| GENE-4691_462 | 7A | 202.74 * | 3.16 | 0.335 | 0.055 | E1 |  |
| Tdurum_contig20378_260 | 7A | 736.43 | 3.81 | 0.296 | 0.069 | E1 |  |
| RAC875_c19880_1414 | 7B | 218.56 | 3.85 | 0.134 | 0.069 | E2 | Selective region |
| RAC875_c48891_476 | Un | 124.96 | 4.36 | 0.152 | 0.081 | E4 |  |

*SNPs belong to the clusters.

Chr.: chromosome, Env.: environment, Ref.: reference, reported QTLs in the references.

**Supplementary Table 10** Significant SNPs associated with spikelet number detected by GWAS.

| **SNP** | **Chr.** | **Pos. (Mb)** | **-Log_10_*P*** | **MAF** | ***R*^2^** | **Env.** | **Ref.** |
| --- | --- | --- | --- | --- | --- | --- | --- |
| wsnp_JD_c9290_10155594 | 1A | 28.61 | 3.49 | 0.361 | 0.055 | E4 |  |
| GENE-0507_285 | 1A | 31.78 | 3.09 | 0.361 | 0.042 | E2 |  |
| GENE-0168_7 | 1A | 179.48 | 3.51 | 0.126 | 0.050 | E2 |  |
| RAC875_c33300_141 | 1A | 354.30 | 3.18 | 0.390 | 0.041 | E1 |  |
| Kukri_rep_c103147_745 | 1A | 356.47 | 4.40 | 0.377 | 0.061 | E1 |  |
| wsnp_BF482891A_Ta_2_2 | 1A | 357.42 | 3.54 | 0.382 | 0.047 | E1 |  |
| Kukri_c88698_296 | 1A | 464.84 | 3.07 | 0.355 | 0.047 | E4 |  |
| Excalibur_c42301_54 | 1B | 643.26 | 3.44/4.18 | 0.343/0.347 | 0.052/0.068 | E3/E4 | Ma et al. 2007 |
| BS00067564_51 | 1D | 420.07 | 3.02/4.07 | 0.452/0.459 | 0.044/0.066 | E3/E4 | Selective region |
| BS00020363_51 | 1D | 459.89 | 3.01 | 0.125 | 0.044 | E3 |  |
| Ku_c105361_414 | 2A | 770.01 | 3.13 | 0.134 | 0.048 | E4 |  |
| Ku_c6050_678 | 2B | 529.10 | 3.23 | 0.157 | 0.042 | E1 |  |
| Kukri_rep_c72585_111 | 3A | 62.41 | 3.04 | 0.131 | 0.039 | E1 |  |
| Kukri_c7218_1145 | 3A | 504.80 | 3.37 | 0.317 | 0.052 | E4 |  |
| Excalibur_rep_c114451_411 | 3A | 631.58 | 3.08 | 0.160 | 0.045 | E3 |  |
| BS00090179_51 | 3B | 177.97 * | 3.20 | 0.115 | 0.041 | E1 |  |
| BS00090178_51 | 3B | 177.97 * | 3.34 | 0.110 | 0.044 | E1 |  |
| Tdurum_contig43874_1129 | 5A | 460.52 | 3.38 | 0.475 | 0.053 | E4 |  |
| Tdurum_contig11411_100 | 5A | 596.57 | 3.23 | 0.126 | 0.050 | E4 |  |
| Ex_c24031_284 | 5B | 707.13 | 3.20 | 0.251 | 0.044 | E2 |  |
| TA003860-0675 | 7A | 8.37 | 3.11/4.65 | 0.460/0.467 | 0.046/0.077 | E3/E4 | Ma et al. 2007  Selective region |
| Excalibur_c36808_228 | 7A | 199.01 | 3.02 | 0.133 | 0.044 | E3 | Selective region |
| Excalibur_c36221_1031 | 7B | 716.59 * | 4.00/3.61 | 0.181/0.183 | 0.054/0.057 | E1/E4 |  |
| IACX5767 | 7B | 716.96 * | 3.81/3.67 | 0.204/0.208 | 0.051/0.058 | E1/E4 |  |
| BS00003630_51 | 7B | 716.96 * | 3.81/3.67 | 0.204/0.208 | 0.051/0.058 | E1/E4 |  |
| BS00009879_51 | 7B | 716.97 * | 3.76/3.18 | 0.183/0.186 | 0.050/0.049 | E1/E4 |  |
| BS00089322_51 | 7B | 716.97 * | 3.92/3.23 | 0.181/0.183 | 0.053/0.050 | E1/E4 |  |
| BobWhite_c2260_168 | 7D | 9.31 | 4.35 | 0.456 | 0.071 | E4 |  |

*SNPs belong to the clusters.

Chr.: chromosome, Env.: environment, Ref.: reference, reported QTLs in the references.

**Supplementary Table 11** Significant SNPs associated with kernels per spike detected by GWAS.

| **SNP** | **Chr.** | **Pos. (Mb)** | **-Log_10_*P*** | **MAF** | ***R*^2^** | **Env.** | **Reference** |
| --- | --- | --- | --- | --- | --- | --- | --- |
| Excalibur_c94756_540 | 1A | 34.17 * | 3.45 | 0.405 | 0.048 | E2 |  |
| wsnp_Ku_rep_c70742_70379526 | 1A | 34.17 * | 3.16 | 0.411 | 0.044 | E2 |  |
| Excalibur_c44668_382 | 1A | 516.45 | 3.01 | 0.242 | 0.043 | E4 | Yuan et al. 2012 |
| Ra_c16879_977 | 1B | 52.35 | 3.08 | 0.268 | 0.042 | E2 | Selective region |
| BS00066880_51 | 1B | 54.96 | 3.20 | 0.384 | 0.044 | E2 |  |
| TA003900-0407 | 1B | 501.37 | 3.09 | 0.319 | 0.044 | E4 |  |
| BS00076039_51 | 2A | 3.80 | 3.42 | 0.192 | 0.048 | E2 |  |
| TA002095-0637 | 2A | 5.53 | 3.64 | 0.279 | 0.052 | E2 |  |
| Excalibur_c6660_746 | 2A | 691.22 * | 3.19 | 0.223 | 0.046 | E4 | Jia et al. 2013 |
| wsnp_Ku_c8927_15048149 | 2A | 691.22 * | 3.21 | 0.214 | 0.047 | E4 |  |
| Kukri_c7193_3176 | 2A | 691.22 * | 3.44 | 0.225 | 0.051 | E4 |  |
| Kukri_c7193_5892 | 2A | 691.23 * | 3.73 | 0.253 | 0.056 | E4 |  |
| Kukri_c29170_680 | 2A | 693.29 | 3.05 | 0.217 | 0.044 | E4 | Jia et al. 2013 |
| Excalibur_c74622_265 | 2B | 775.83 * | 4.35 | 0.069 | 0.058 | E1 | Yuan et al. 2012 |
| Kukri_c16161_231 | 2B | 776.70 * | 4.40 | 0.128 | 0.059 | E1 |  |
| Excalibur_c40335_454 | 2B | 776.75 * | 4.96 | 0.125 | 0.068 | E1 |  |
| Kukri_c26697_366 | 2B | 776.98 * | 3.11 | 0.186 | 0.039 | E1 |  |
| Ra_c1597_2503 | 2B | 776.98 * | 4.76 | 0.117 | 0.065 | E1 |  |
| Excalibur_c16329_370 | 2B | 777.33 * | 4.96 | 0.125 | 0.068 | E1 |  |
| Ex_c27573_778 | 2B | 777.51 * | 4.08 | 0.125 | 0.054 | E1 |  |
| wsnp_Ex_c1763_3333974 | 3A | 134.60 | 3.31 | 0.395 | 0.042 | E3 |  |
| RAC875_c16846_1247 | 3A | 135.99 | 3.48 | 0.392 | 0.045 | E3 |  |
| BS00098840_51 | 3B | 182.38 * | 3.35 | 0.400 | 0.043 | E3 |  |
| TA005199-0585 | 3B | 182.38 * | 3.35 | 0.400 | 0.043 | E3 |  |
| Kukri_rep_c101259_159 | 4B | 657.27 | 3.17 | 0.427 | 0.040 | E3 |  |
| GENE-2531_86 | 4D | 457.50 | 3.61 | 0.270 | 0.047 | E3 |  |
| BobWhite_c40643_370 | 5A | 575.60 * | 3.51 | 0.378 | 0.045 | E1 | Shi et al. 2017 |
| Tdurum_contig56267_180 | 5A | 575.60 * | 3.29 | 0.375 | 0.042 | E1 |  |
| Jagger_c5071_425 | 5B | 697.12 * | 3.56 | 0.197 | 0.046 | E1 |  |
| Kukri_c88397_242 | 5B | 697.38 * | 3.53 | 0.210 | 0.045 | E1 |  |
| Excalibur_c24213_314 | 6A | 490.50 | 4.05 | 0.203 | 0.059 | E2 |  |
| Excalibur_c51068_220 | 6A | 603.19 | 3.01 | 0.186 | 0.038 | E1 |  |
| RAC875_c21938_802 | 6A | 614.80 | 3.25 | 0.184 | 0.041 | E3 | Selective region |
| Kukri_c35951_337 | 6D | 258.36 | 3.17 | 0.092 | 0.040 | E3 | TaGW2-A1  (41.93_308.29Mb) |
| Excalibur_c36221_1031 | 7B | 716.59 * | 4.18 | 0.184 | 0.056 | E1 | Cui et al. 2014  Liu et al. 2014  Yu et al. 2018 |
| BS00003630_51 | 7B | 716.96 * | 3.63 | 0.207 | 0.047 | E1 |  |
| IACX5767 | 7B | 716.96 * | 3.63 | 0.207 | 0.047 | E1 |  |
| BS00009879_51 | 7B | 716.97 * | 3.45 | 0.186 | 0.044 | E1 |  |
| BS00089322_51 | 7B | 716.97 * | 3.61 | 0.184 | 0.047 | E1 |  |

*SNPs belong to the clusters.

**Supplementary Table 12** Significant SNPs associated with thousand kernel weight detected by GWAS.

| **SNP** | **Chr.** | **Pos. (Mb)** | **-Log_10_*P*** | **MAF** | ***R*^2^** | **Environment** | **Reference** |
| --- | --- | --- | --- | --- | --- | --- | --- |
| Tdurum_contig56538_337 | 1A | 547.73 | 3.18 | 0.126 | 0.026 | E2 |  |
| Kukri_c13329_800 | 2A | 61.47 | 3.46 | 0.321 | 0.030 | E4 |  |
| GENE-0872_343 | 2A | 657.52 | 4.33 | 0.132 | 0.039 | E4 | McCartney et al. 2005 |
| IAAV3757 | 2A | 758.42 | 3.84/3.82 | 0.174 | 0.031/0.034 | E3/E4 |  |
| Excalibur_c9525_105 | 2A | 769.22 | 3.55 | 0.055 | 0.029 | E2 |  |
| BobWhite_c1275_359 | 2D | 614.64 | 3.56/3.10 | 0.395 | 0.027/0.025 | E1/E2 |  |
| TA002948-0488 | 2D | 622.93 * | 3.48 | 0.226 | 0.029 | E2 |  |
| Kukri_c18754_67 | 2D | 622.93 * | 3.66 | 0.261 | 0.030 | E2 |  |
| Ex_c57212_719 | 4A | 21.63 | 3.34 | 0.432 | 0.026 | E3 | Liu et al. 2017 |
| wsnp_Ku_rep_c76865_75281903 | 4A | 60.66 | 3.14 | 0.289 | 0.027 | E4 |  |
| BS00062122_51 | 4A | 690.98 | 3.49 | 0.389 | 0.027 | E3 |  |
| BS00022090_51 | 4B | 139.76 | 3.24 | 0.178 | 0.024 | E1 | Wang et al. 2011 |
| BS00058659_51 | 4B | 555.21 * | 3.17 | 0.426 | 0.024 | E3 | Nezhad et al. 2012 |
| wsnp_Ex_c42895_49355806 | 4B | 556.06 * | 3.37 | 0.421 | 0.026 | E3 |  |
| Tdurum_contig37154_190 | 5B | 289.57 | 3.41 | 0.395 | 0.029 | E4 |  |
| BS00023064_51 | 5B | 595.66 | 3.07 | 0.217 | 0.022 | E1 |  |
| Tdurum_contig10412_650 | 6A | 80.70 | 3.24 | 0.405 | 0.028 | E4 | TaGW2-A1  (52.40_445.25Mb)  Selective region |
| wsnp_Ex_c2268_4251636 | 7A | 93.08 | 3.15 | 0.337 | 0.024 | E3 | Tang et al. 2011 |
| GENE-4598_467 | 7A | 106.20 * | 3.39 | 0.395 | 0.028 | E2 | Tang et al. 2011 |
| GENE-4862_674 | 7A | 106.24 * | 3.39 | 0.395 | 0.028 | E2 |  |
| TA004831-0260 | 7A | 106.25 * | 3.39 | 0.395 | 0.028 | E2 |  |
| BS00009043_51 | 7A | 701.29 | 3.07 | 0.176 | 0.025 | E2 |  |
| Kukri_rep_c79716_389 | 7A | 717.04 | 3.01 | 0.321 | 0.023 | E3 | Li et al. 2015 |
| RAC875_c10672_440 | 7B | 40.35 | 3.68 | 0.347 | 0.032 | E4 |  |
| GENE-4862_1104 | 7B | 59.65 | 3.18 | 0.392 | 0.026 | E2 |  |
| BS00022810_51 | 7B | 710.99 | 3.12 | 0.332 | 0.026 | E4 |  |
| BS00075615_51 | 7B | 718.14 * | 3.01 | 0.321 | 0.023 | E3 |  |
| Kukri_rep_c79716_287 | 7B | 718.47 * | 3.01 | 0.321 | 0.023 | E3 |  |
| Kukri_c48418_149 | 7B | 718.47 * | 3.43 | 0.384 | 0.027 | E3 |  |
| Kukri_rep_c68139_172 | Un | 124.83 | 3.36 | 0.403 | 0.025 | E1 |  |

*SNPs belong to the clusters.

**Supplementary Table 13** Significant SNPs associated with spike number per plant detected by GWAS.

| **SNP** | **Chr.** | **Pos. (Mb)** | **-Log_10_*P*** | **MAF** | ***R*^2^** | **Env.** | **Reference** |
| --- | --- | --- | --- | --- | --- | --- | --- |
| BobWhite_c15603_69 | 1A | 305.37 * | 3.37 | 0.353 | 0.060 | E1 | Naruoka et al. 2011 |
| IAAV7757 | 1A | 305.37 * | 3.27 | 0.355 | 0.058 | E1 |  |
| Kukri_c1529_462 | 1B | 92.33 | 3.10 | 0.335 | 0.044 | E4 |  |
| wsnp_Ex_c23992_33235984 | 1B | 532.17 * | 3.08 | 0.293 | 0.043 | E4 |  |
| Ra_c1652_588 | 1B | 532.17 * | 3.29 | 0.250 | 0.047 | E4 |  |
| wsnp_Ex_c9248_15372536 | 2B | 158.62 * | 3.70 | 0.277 | 0.054 | E4 |  |
| wsnp_Ex_c45094_50985067 | 2B | 158.64 * | 3.46 | 0.266 | 0.050 | E4 |  |
| BS00108016_51 | 2D | 10.91 | 3.48 | 0.434 | 0.057 | E2 |  |
| D_GBUVHFX01CSU22_382 | 2D | 13.91 | 3.06 | 0.058 | 0.053 | E1 | Suenaga et al. 2005 |
| TA002217-0946 | 2D | 545.73 | 3.57 | 0.179 | 0.060 | E3 |  |
| BobWhite_c48254_364 | 3A | 523.32 * | 3.35 | 0.153 | 0.056 | E3 |  |
| Kukri_rep_c70927_886 | 3A | 523.55 * | 3.35 | 0.153 | 0.056 | E3 |  |
| RAC875_c6932_1229 | 3A | 523.55 * | 3.42 | 0.145 | 0.057 | E3 |  |
| BobWhite_c23828_341 | 3A | 690.86 | 3.10 | 0.253 | 0.054 | E1 |  |
| wsnp_Ex_c361_707953 | 3A | 732.47 * | 3.05 | 0.205 | 0.053 | E1 |  |
| IAAV2423 | 3A | 732.78 * | 3.77 | 0.389 | 0.069 | E1 |  |
| Tdurum_contig18326_142 | 3A | 737.44 | 3.63 | 0.224 | 0.066 | E1 |  |
| RAC875_c21489_908 | 3B | 357.52 | 3.36 | 0.361 | 0.054 | E2 |  |
| Kukri_c29420_310 | 5A | 9.32 | 3.53 | 0.125 | 0.051 | E4 | Selective region |
| BobWhite_c2001_586 | 5A | 462.17 | 3.13 | 0.444 | 0.044 | E4 |  |
| BS00067170_51 | 5A | 580.49 | 3.12/4.48 | 0.132/0.128 | 0.050/0.068 | E2/E4 | Vrn-A1(587.42 Mb);  Kato et al. 2000  Quarrie et al. 2005 |
| Excalibur_c33199_1430 | 6A | 3.63 | 3.19 | 0.142 | 0.051 | E2 |  |
| BS00066872_51 | 6A | 12.14 * | 3.21 | 0.184 | 0.053 | E3 |  |
| Tdurum_contig42405_114 | 6A | 12.14 * | 3.07 | 0.179 | 0.050 | E3 |  |
| Tdurum_contig47140_554 | 6B | 567.60 * | 3.17 | 0.197 | 0.052 | E3 |  |
| Tdurum_contig47140_507 | 6B | 567.60 * | 4.05 | 0.200 | 0.070 | E3 |  |
| Tdurum_contig51001_186 | 7A | 1.72 * | 3.27 | 0.232 | 0.053 | E2 |  |
| BS00033780_51 | 7A | 2.70 * | 3.28 | 0.079 | 0.053 | E2 |  |
| Tdurum_contig75811_536 | 7A | 3.85 | 3.07 | 0.145 | 0.049 | E2 |  |
| wsnp_CAP7_c10100_4479468 | 7A | 21.35 | 3.20 | 0.471 | 0.056 | E1 |  |
| RAC875_c10841_56 | 7A | 345.21 | 3.08 | 0.200 | 0.049 | E2 |  |
| BobWhite_c26201_275 | 7A | 379.03 | 3.21 | 0.216 | 0.052 | E2 |  |
| Excalibur_s112378_98 | 7A | 392.92 | 3.16 | 0.284 | 0.055 | E1 |  |
| Excalibur_rep_c102881_88 | 7A | 694.55 * | 3.02 | 0.153 | 0.048 | E2 | TaCML20(692.90Mb) |
| CAP7_rep_c10402_310 | 7A | 694.55 * | 3.16 | 0.150 | 0.051 | E2 |  |
| D_contig25677_304 | 7D | 104.75 | 3.41 | 0.100 | 0.057 | E3 | Selective region |

*SNPs belong to the clusters.

**Supplementary Table 14** QTLs associated with SL, KPS, SN and TKW on 2A, 2B, 2D, 6A and 7B detected in this study and previously reported.

| **QTL Name** | **Flanking markers** | **Physical position (bp)** | **References** |
| --- | --- | --- | --- |
| *qSL-2A* | *IWB1047–IWA2005* | 77939322–78335426 |  |
| *Selection-2A.7* |  | 77900001–78000000 |  |
| *Selection-2A.8* |  | 78300001–78400000 |  |
| *Selection-2A.15* |  | 125200001–125300000 |  |
| *qSL-2A.1* | *Xwmc407* | 7808728 | Yao et al. 2009 |
| *qSL-2A.2* | *A28189* | 77942011 | Liu et al. 2017 |
| *qSL-2A.3* | *Xgwm339* | 112746128 | Yao et al. 2009 |
| *qSL-2A.4* | *Xwmc474* | 123205810 | Yao et al. 2009 |
| *qSL-2A.5* | *Xgdm101* | 142757065 | Yao et al. 2009 |
| *qSL-2A.6* | *Xgwm372* | 203314944 | Liu et al. 2006 |
| *qSL-2A.7* | *Xwmc702* | 696427753 | Yao et al. 2009/Jaiswal et al. 2016 |
| *qSL-2A.8* | *Xgpw2206* | 709697740 | Lee et al. 2014 |
| *qSL-2A.9* | *Xgwm294* | 716545623 | Yao et al. 2009 |
| *qSL-2B* | *IWB14055–IWB27823* | 233268307–233554407 |  |
| *Selection-2B.4* |  | 759700001–759800000 |  |
| *qSL-2B.1* | *wPt-3632* | 28412439 | Neumann et al. 2011 |
| *qSL-2B.2* | *IWB46375* | 31622741 | Zhai et al. 2016; Xu et al. 2014 |
| *qSL-2B.3* | *Xwmc154* | 36446499 | Xu et al. 2017 |
| *qSL-2B.4* | *IWB6886* | 52701712 | Sun et al. 2017 |
| *qSL-2B.5* | *3064921* | 91046000 | Mwadzingeni et al. 2017 |
| *qSL-2B.6* | *Xwmc344* | 165578567 | Liu et al. 2014 |
| *qSL-2B.7* | *Xwmc474–Xwmc272* | 172687312–257897748 | Kumar et al. 2007 |
| *qSL-2B.8* | *3944716* | 757221944 | Mwadzingeni et al. 2017 |
| *qSL-2B.9* | *wPt-0047* | 774499699 | Xu et al. 2017 |
| *qSL-2B.10* | *wPt-2266* | 779287326 | Neumann et al. 2011 |
| *qSL-2B.11* | *1029432* | 797587667 | Mwadzingeni et al. 2017 |
| *qSL-2D* | *IWB26541–IWB74078* | 608199256–608918858 |  |
| *Selection-2D.2* |  | 599000001–599100000 |  |
| *Selection-2D.3* |  | 600200001–600300000 |  |
| *qSL-2D.1* | *IWA5344–Xgwm132* | 14401034–21741656 | Li et al. 2016 |
| *qSL-2D.2* | *Xgwm296* | 17609164 | Wu et al. 2012 |
| *qSL-2D.3* | *Xgwm261–Xcfd53* | 19623173–23024763 | Heidari et al. 2011/Trkulja et al. 2012/Sourdille et al. 2000/2003/2005 |
| *qSL-2D.4* | *Xwmc503–Xwmc112* | 19631458–23024735 | Wang et al. 2011/Katkout et al. 2014 |
| *qSL-2D.5* | *Xwmc112–Xcfd53* | 23024735–23024763 | Wu et al. 2012/Suenaga et al. 2005/Xu et al. 2012/Zhai et al. 2016 |
| *qSL-2D.6* | *Xgwm515* | 120026588 | Liu et al. 2006 |
| *qSL-2D.7* | *Xwmc144* | 267082590 | Wu et al. 2012 |
| *qSL-2D.8* | *Xgpw8003* | 478111878 | Lee et al. 2014 |
| *qSL-2D.9* | *IN10–Xmag4089* | 480173412–611212329 | Fan et al. 2015 |
| *qSL-2D.10* | *4021827* | 593909373 | Mwadzingeni et al. 2017 |
| *qSL-2D.11* | *Xgwm349–Xgwm382* | 629648566–647509961 | Kumar et al. 2007 |
| *qSL-2D.12* | *Xgwm320* | 644277498 | Liu et al. 2006 |
| *qSL-2D.13* | *wPt-6343–wPt-8319* | 645090679–648059139 | Neumann et al. 2011 |
| *qSL-6A* | *IWB58592–IWB45584* | 60168056–61388308 |  |
| *Selection-6A.1* |  | 82100001–82200000 |  |
| *Selection-6A.2* |  | 84900001–85000001 |  |
| *qSL-6A.1* | *Xbarc195* | 79595520 | Yu et al. 2014 |
| *qSL-6A.2* | *Xcfe273–Xpsp3152* | 81121662–545130117 | Cui et al. 2011 |
| *qSL-6A.3* | *3944784* | 108596911 | Mwadzingeni et al. 2017 |
| *qSL-6A.4* | *Xbarc146–WABM103423* | 166990489–409134409 | Iehisa et al. 2014; Heidari et al. 2011 |
| *qSL-6A.5* | *Xpsp3029–Xgwm497* | 292281720–613628842 | Heidari et al. 2011 |
| *qSL-6A.6* | *Xbarc37–Xbarc1055* | 313925657–383858341 | Wang et al. 2011 |
| *qSL-6A.7* | *Xwmc256* | 549524079 | Lee et al. 2014 |
| *qSL-6A.8* | *Xwmc201–Xgwm570* | 574283851–579125980 | Liu et al. 2014 |
| *qSL-6A.9* | *IWA4809* | 596383684 | Guo et al. 2018 |
| *qSL-6A.10* | *Xgwm617* | 604264513 | Suenaga et al. 2005 |
| *qKPS-2A* | *IWB28454–IWB47527* | 691218436–691228555 |  |
| *Selection-2A.2* |  | 65400001–65500000 |  |
| *Selection-2A.8* |  | 78300001–78400000 |  |
| *Selection-2A.9* |  | 82300001–82400000 |  |
| *Selection-2A.26* |  | 611000001–611100000 |  |
| *qKPS-2A.1* | *Xbarc212* | 1582751 | Yao et al. 2009 |
| *qKPS-2A.2* | *IWB32223* | 12961250 | Shi et al. 2017 |
| *qKPS-2A.3* | *Xwmc522–Xcfa2263* | 59547150–88325962 | Wang et al. 2009 |
| *qKPS-2A.4* | *Xwmc296/Xgwm122* | 80871933/80872065 | Yao et al. 2009 |
| *qKPS-2A.5* | *Xpsp3029–wPt-6361* | 292281720–586067493 | Cui et al. 2014 |
| *qKPS-2A.6* | *Xbarc15* | 469117522 | Xu et al. 2017 |
| *qKPS-2A.7* | *IWB7310* | 612845080 | Shi et al. 2017 |
| *qKPS-2A.8* | *Xgwm47–Xwmc181* | 685759420–728609562 | Jia et al. 2013 |
| *qKPS-2A.9* | *Xgwm312* | 709048504 | Yao et al. 2009 |
| *qKPS-2A.10* | *Xgpw2206* | 709697740 | Lee et al. 2014 |
| *qKPS-2A.11* | *Xgwm294* | 716545623 | Yao et al. 2009 |
| *qKPS-2A.12* | *IWB73792* | 772373155 | Shi et al. 2017 |
| *qKPS-2B* | *IWB28725–IWB20376* | 775828691–777514658 |  |
| *Selection-2B.1* |  | 8300001–8400000 |  |
| *Selection-2B.3* |  | 622900001–623000000 |  |
| *qKPS-2B.1* | *Xwmc764* | 8072794 | Azadi et al. 2015 |
| *qKPS-2B.2* | *wPt-3632* | 28412439 | Neumann et al. 2011 |
| *qKPS-2B.3* | *IWB9943* | 31422132 | Guo et al. 2017 |
| *qKPS-2B.4* | *Xgwm71–Xgwm429* | 31827848–73618172 | Heidari et al. 2011/Ge et al. 2012 |
| *qKPS-2B.5* | *IWB49203* | 33839886 | Guo et al. 2017 |
| *qKPS-2B.6* | *Xbarc200–Xwmc770* | 47539141–88833296 | Yuan et al. 2012 |
| *qKPS-2B.7* | *Xgwm410* | 95335174 | Ge et al. 2012 |
| *qKPS-2B.8* | *IWB6599* | 122267555 | Shi et al. 2017 |
| *qKPS-2B.9* | *IWB8099* | 184661676 | Shi et al. 2017 |
| *qKPS-2B.10* | *Xbarc1027–Xwmc441* | 308582098–598064477 | Tang et al. 2011 |
| *qKPS-2B.11* | *Xgwm388–Xwmc175* | 557367540–670596947 | Yuan et al. 2012 |
| *qKPS-2B.12* | *Xbarc101–Xcwem55* | 621466535–800849725 | Yuan et al. 2012 |
| *qKPS-2B.13* | *Xcfd283–Xgwm501* | 634419388–672082697 | Liu et al. 2014/Ge et al. 2012 |
| *qKPS-2B.14* | *Xwmc360* | 718351630 | Ge et al. 2012 |
| *qKPS-2B.15* | *IWA5809* | 746036594 | Shi et al. 2017 |
| *qKPS/SN-7B* | *IWB25689–IWB11608* | 716594854–716966341 |  |
| *Selection-7B.4* |  | 211400001–211500000 |  |
| *Selection-7B.5* |  | 218500001–218600000 |  |
| *Selection-7B.6* |  | 478800001–478900000 |  |
| *qKPS-7B.1* | *Xbarc50–Xcfe223* | 172356933–743620415 | Wang et al. 2011 |
| *qKPS-7B.2* | *Xgwm68–Xbarc65* | 176032229–452495629 | Yu et al. 2018; Cui et al. 2014 |
| *qKPS-7B.3* | *wPt-2273* | 220309759 | Cui et al. 2014 |
| *qKPS-7B.4* | *IWB33961* | 389110565 | Shi et al. 2017 |
| *qKPS-7B.5* | *Xwmc338–Xbarc346* | 425099572–723009479 | Cui et al. 2014 |
| *qKPS-7B.6* | *Xbarc65–Xbarc1073* | 452495629–723211497 | Yu et al. 2018; Cui et al. 2014; Liu et al. 2014 |
| *qKPS-7B.7* | *IWA6667* | 457401734 | Guo et al. 2018 |
| *qKPS-7B.8* | *Xgwm333–Xwmc396* | 475645851–546855418 | Yuan et al. 2012 |
| *qKPS-7B.9* | *wPT-9467–wPT-4025* | 553623133–592615323 | Ma et al. 2012 |
| *qKPS-7B.10* | *Xwmc273–Xbarc1073* | 718432540–723211497 | Liu et al. 2014; Cui et al. 2014 |
| *qSN-7B.1* | *wPt-2883* | 23594887 | Ogbonnaya et al. 2017 |
| *qSN-7B.2* | *wPt-2273–wPt-3533* | 220309759–670840881 | Yu et al. 2014 |
| *qSN-7B.3* | *Xbarc176* | 557048410 | Yu et al. 2018 |
| *qSN-7B.4* | *Xbarc1181–Xbarc278* | 550104590–595079803 | Wang *et al.* 2011 |
| *qSN-7B.5* | *Xwmc517–Xwmc311* | 651500486–690871145 | Wang *et al.* 2011 |
| *qTKW-2D* | *IWB400–IWB42028* | 614644234–622925468 |  |
| *Selection-2D.1* |  | 122700001–122800000 |  |
| *Slection-2D.2* |  | 599000001–599100000 |  |
| *Selection-2D.3* |  | 600200001–600300000 |  |
| *qTKW-2D.1* | *Xcfd56–Xcfd11* | 6158983–79231506 | Ge et al. 2012; Groos et al. 2003 |
| *qTKW-2D.2* | *Xgpw5122–wPt-731489* | 6709919–9339991 | Yu et al. 2018 |
| *qTKW-2D.3* | *Xbcd102* | 15133811 | Boner et al. 2002 |
| *qTKW-2D.4* | *Xgwm261* | 19623173 | Ge et al. 2012; Groos et al. 2003 |
| *qTKW-2D.5* | *Xgdm6* | 23024735 | Huang et al. 2003,2004 |
| *qTKW-2D.6* | *Xcfd53–Xbarc168* | 23024763–44736965 | Heidari et al. 2011 |
| *qTKW-2D.7* | *Xcfd53–wPt-4144* | 23024763–66871862 | Cui et al. 2014; Wu et al. 2011; Heidari et al. 2011 |
| *qTKW-2D.8* | *wPt-6867–wPt-6133* | 25305584–29534516 | Yu et al. 2018 |
| *qTKW-2D.9* | *wPt-666223–Xgpw4473* | 27981473–38222772 | Yu et al. 2018; Yu et al. 2014 |
| *qTKW-2D.10* | *Xgpw4473–wPt-665328* | 38222772–43976324 | Yu et al. 2018; Yu et al. 2014 |
| *qTKW-2D.11* | *Xgwm484* | 48174728 | Mason et al. 2013 |
| *qTKW-2D.12* | *wPt-666857* | 60168498 | Azadi et al. 2015; Cuthbert et al.2008; Huang et al. 2006; Huang et al. 2004 |
| *qTKW-2D.13* | *Xgwm515–Xbarc228* | 120026697–383000396 | Yu et al. 2018; Ge et al. 2012 |
| *qTKW-2D.14* | *Xwmc18* | 130832852 | Reif *et al.* 2011 |
| *qTKW-2D.15* | *wPt-0298* | 170364558 | Azadi et al. 2015 |
| *qTKW-2D.16* | *Xwmc601* | 332519440 | Cuthbert et al. 2008 |
| *qTKW-2D.17* | *Xbarc228–Xcfd168* | 383000396–580011701 | Yu et al. 2018/Cui et al. 2014/Wang *et al.* 2009/Ge et al. 2012 |
| *qTKW-2D.18* | *Xgwm539–IWA5252* | 513098578–552192900 | Li et al. 2015/ Ramya et al. 2010 |
| *qTKW-2D.19* | *Xgwm539–Xcfd168* | 513098578–580011701 | Zhang et al. 2014; Wang et al. 2009 |
| *qTKW-2D.20* | *wPt-666518* | 537011849 | Luo et al. 2016 |
| *qTKW-2D.21* | *Xcfd233* | 561157752 | Ramya et al. 2010 |
| *qTKW-2D.22* | *wPt-2781–wPt-666332* | 574352149–594593062 | Cui et al. 2014; Wu et al. 2011; Cuthbert et al. 2008 |
| *qTKW-2D.23* | *Xcfd168–Xcfd44* | 580011701–608633134 | Cui et al. 2014; Wang et al. 2009 |
| *qTKW-2D.24* | *Xwmc181* | 593738635 | Wu et al. 2011 |
| *qTKW-2D.25* | *Xwmc112* | 599941936 | Huang et al. 2006 |
| *qTKW-2D.26* | *Xgwm382* | 647509961 | Cuthbert et al. 2008 |

SL: spike length, SN: spikelet number, KPS: kernels per spike, TKW: thousand kernel weight.

**Supplementary Table 15** The biological function of candidate genes detected by the selection signal analysis.

| **Gene_Name** | **START (bp)** | **END (bp)** | **Description** |
| --- | --- | --- | --- |
| TraesCS1A01G179000 | 323600001 | 323700000 | Development (germination and root growth) |
| TraesCS1A01G179600 | 324500001 | 324600000 | Metabolism (protein degradation) |
| TraesCS1A01G441100 | 589700001 | 589800000 | nuclease |
| TraesCS1A01G441500 | 590200001 | 590300000 | Stress response (disease resistance) |
| TraesCS1B01G022900 | 10200001 | 10300000 | Development (root, flowering time, floral organ number ) |
| TraesCS1B01G024100 | 11600001 | 11700000 | TE |
| TraesCS1B01G024200 | 11600001 | 11700000 | Stress response |
| TraesCS1B01G024300 | 11600001 | 11700000 | Development |
| TraesCS1B01G024400 | 11600001 | 11700000 | Signaling |
| TraesCS1B01G180300 | 327000001 | 327100000 | NA |
| TraesCS1B01G272000 | 478000001 | 478100000 | Signaling |
| TraesCS1B01G272100 | 478000001 | 478100000 | Metabolism (lipid catabolic) |
| TraesCS1B01G272800 | 478700001 | 478800000 | Transcription |
| TraesCS1B01G272900 | 478700001 | 478800000 | Stress response |
| TraesCS2A01G125200 | 74100001 | 74200000 | Transcription |
| TraesCS2A01G125300 | 74100001 | 74200000 | Development |
| TraesCS2A01G125400 | 74100001 | 74200000 | NA |
| TraesCS2A01G125500 | 74100001 | 74200000 | NA |
| TraesCS2A01G125600 | 74100001 | 74200000 | Development (embryo morphogenesis and apical-basal pattern formation) |
| TraesCS2A01G125700 | 74100001 | 74200000 | NA |
| TraesCS2A01G125800 | 74100001 | 74200000 | Metabolism (protein catabolic) |
| TraesCS2A01G127100 | 75100001 | 75200000 | Stress response (drought, light) |
| TraesCS2A01G127200 | 75100001 | 75200000 | Metabolism |
| TraesCS2A01G130200 | 77900001 | 78000000 | Metabolism |
| TraesCS2A01G130500 | 78300001 | 78400000 | Transcription |
| TraesCS2A01G130600 | 78300001 | 78400000 | Metabolism |
| TraesCS2A01G211600 | 195800001 | 195900000 | NA |
| TraesCS2A01G211700 | 196100001 | 196200000 | NA |
| TraesCS2A01G211900 | 196500001 | 196600000 | Signaling |
| TraesCS2A01G212000 | 196500001 | 196600000 | Development |
| TraesCS2A01G212100 | 196500001 | 196600000 | Transport |
| TraesCS3A01G500400 | 724800001 | 724900000 | Development |
| TraesCS3A01G500500 | 724800001 | 724900000 | Development |
| TraesCS3A01G500600 | 724800001 | 724900000 | Development |
| TraesCS3A01G500700 | 724800001 | 724900000 | Stress response (disease resistance) |
| TraesCS3B01G102800 | 69600001 | 69700000 | Metabolism (purine and pyrimidine catabolism) |
| TraesCS3B01G102900 | 69600001 | 69700000 | Stress response (disease resistance) |
| TraesCS3B01G103000 | 69600001 | 69700000 | Metabolism |
| TraesCS3B01G103100 | 69600001 | 69700000 | Metabolism (lipase) |
| TraesCS3B01G103200 | 69800001 | 69900000 | Transcription |
| TraesCS3B01G103200 | 69800001 | 69900000 | Transcription |
| TraesCS3D01G507400 | 593900001 | 594000000 | Development |
| TraesCS3D01G507500 | 593900001 | 594000000 | Development (pollen, seed germination and development) |
| TraesCS5A01G275800 | 485200001 | 485300000 | Membrane |
| TraesCS5A01G275900 | 485200001 | 485300000 | Transcription |
| TraesCS5A01G276500 | 485500001 | 485600000 | Metabolism |
| TraesCS5A01G467600 | 645400001 | 645500000 | Transport |
| TraesCS5A01G467700 | 645400001 | 645500000 | Metabolism |
| TraesCS5A01G467800 | 645400001 | 645500000 | Signaling |
| TraesCS5A01G467900 | 645600001 | 645700000 | Development |
| TraesCS5A01G468000 | 645600001 | 645700000 | NA |
| TraesCS5A01G468100 | 645600001 | 645700000 | NA |
| TraesCS5A01G482700 | 654100001 | 654200000 | Development (root, leaf, flower) |
| TraesCS5A01G482800 | 654100001 | 654200000 | Photosynthesis |
| TraesCS5A01G482900 | 654100001 | 654200000 | Development |
| TraesCS5A01G483000 | 654100001 | 654200000 | Transport |
| TraesCS5A01G483100 | 654100001 | 654200000 | Transport |
| TraesCS5A01G549600 | 703300001 | 703400000 | Oxidation-reduction |
| TraesCS5A01G549700 | 703300001 | 703400000 | Development (cell differentiation, integument development,) |
| TraesCS5A01G550700 | 704300001 | 704400000 | Transcription |
| TraesCS5A01G550800 | 704300001 | 704400000 | Transcription |
| TraesCS5A01G551000 | 704500001 | 704600000 | Cyclin |
| TraesCS5A01G551100 | 704500001 | 704600000 | Metabolism |
| TraesCS5B01G286800 | 473100001 | 473200000 | Metabolism |
| TraesCS5B01G286900 | 473100001 | 473200000 | Metabolism (protein catabolic) |
| TraesCS5B01G287000 | 473100001 | 473200000 | Metabolism |
| TraesCS5B01G287400 | 473600001 | 473700000 | Oxidation-reduction |
| TraesCS5B01G287500 | 473600001 | 473700000 | Stress response (oxygen) |
| TraesCS5B01G287600 | 473600001 | 473700000 | Oxidation-reduction |
| TraesCS5B01G287700 | 473600001 | 473700000 | Transport |
| TraesCS5B01G287800 | 473600001 | 473700000 | Metabolism (protein) |
| TraesCS5B01G479900 | 652400001 | 652500000 | Transport |
| TraesCS5B01G480900 | 653400001 | 653500000 | Transcription |
| TraesCS6B01G140900 | 140400001 | 140500000 | Translation |
| TraesCS6B01G141000 | 140400001 | 140500000 | Translation |
| TraesCS6B01G141100 | 140400001 | 140500000 | Metabolism |
| TraesCS6B01G386400 | 661300001 | 661400000 | Oxidation-reduction |
| TraesCS6B01G386500 | 661300001 | 661400000 | Membrane |
| TraesCS6B01G386700 | 661500001 | 661600000 | Photosynthesis |
| TraesCS6B01G386800 | 661500001 | 661600000 | NA |
| TraesCS7A01G015500 | 6700001 | 6800000 | Transcription |
| TraesCS7A01G015600 | 6700001 | 6800000 | protein dimerization |
| TraesCS7A01G015700 | 6700001 | 6800000 | Signaling |
| TraesCS7A01G015800 | 6700001 | 6800000 | Signaling |
| TraesCS7A01G015900 | 6800001 | 6900000 | Metabolism |
| TraesCS7A01G016000 | 6800001 | 6900000 | Transport |
| TraesCS7A01G016100 | 6800001 | 6900000 | Stress response |
| TraesCS7A01G016200 | 6800001 | 6900000 | Oxidation-reduction |
| TraesCS7A01G017200 | 7500001 | 7600000 | Transcription |
| TraesCS7A01G017300 | 7500001 | 7600000 | NA |
| TraesCS7A01G017400 | 7500001 | 7600000 | Development (cell division) |
| TraesCS7A01G017500 | 7500001 | 7600000 | NA |
| TraesCS7A01G017600 | 7500001 | 7600000 | Stress response (cold, hormone) |
| TraesCS7A01G017700 | 7500001 | 7600000 | Development (grain size and organ size) |
| TraesCS7A01G155700 | 108700001 | 108800000 | Development |
| TraesCS7A01G155800 | 108700001 | 108800000 | Transcription |
| TraesCS7A01G156300 | 108900001 | 109000000 | Membrane |
| TraesCS7B01G109200 | 125100001 | 125200000 | NA |
| TraesCS7B01G159900 | 218500001 | 218600000 | Transport |
| TraesCS7B01G160000 | 218500001 | 218600000 | Development (seed maturation, embryo development) |
| TraesCS7B01G160100 | 218500001 | 218600000 | Membrane |
| TraesCS7B01G258200 | 478800001 | 478900000 | Membrane |
| TraesCS7B01G258300 | 478800001 | 478900000 | Metabolism (fatty acid) |
| TraesCS7B01G373000 | 638800001 | 638900000 | [Photoinhibition](https://www.arabidopsis.org/servlets/TairObject?type=keyword&id=13435) |
| TraesCS7D01G155800 | 104200001 | 104300000 | Oxidation-reduction |
| TraesCS7D01G155900 | 104200001 | 104300000 | NA |
| TraesCS7D01G156800 | 104700001 | 104800000 | Metabolism |
| TraesCS7D01G156900 | 104700001 | 104800000 | Metabolism |
| TraesCS7D01G157000 | 104700001 | 104800000 | Transport |

**Supplementary Table 16** The biological function of candidate genes in SNP clusters detected by the GWAS.

| **Cluster** | **GeneName** | **Description** |
| --- | --- | --- |
| cSL-2A | TraesCS2A01G128600 | NA |
| cSL-2A | TraesCS2A01G128700 | Transcription |
| cSL-2A | TraesCS2A01G128800 | Oxidation-reduction |
| cSL-2A | TraesCS2A01G128900 | Development (pollen) |
| cSL-2A | TraesCS2A01G129000 | Development (multicellular organism) |
| cSL-2A | TraesCS2A01G129100 | Metabolism |
| cSL-2A | TraesCS2A01G129200 | NA |
| cSL-2A | TraesCS2A01G129300 | Stress response |
| cSL-2A | TraesCS2A01G129400 | NA |
| cSL-2A | TraesCS2A01G129500 | NA |
| cSL-2A | TraesCS2A01G129600 | NA |
| cSL-2A | TraesCS2A01G129700 | Development (Root)/Signaling |
| cSL-2A | TraesCS2A01G129800 | Stress response |
| cSL-2A | TraesCS2A01G129900 | cell division |
| cSL-2A | TraesCS2A01G130000 | NA |
| cSL-2A | TraesCS2A01G130100 | Photosynthesis |
| cSL-2A | TraesCS2A01G130200 | Metabolism |
| cSL-2A | TraesCS2A01G130300 | NA |
| cSL-2A | TraesCS2A01G130400 | Transcription |
| cSL-2A | TraesCS2A01G130500 | Transcription |
| cSL-2A | TraesCS2A01G130600 | Stress response/ Metabolism (N) |
| cSL-2A | TraesCS2A01G130700 | protein binding |
| cSL-2A | TraesCS2A01G130800 | Development/ Metabolism |
| cSL-2A | TraesCS2A01G130900 | NA |
| cSL-2A | TraesCS2A01G131000 | Stress response |
| cSL-2A | TraesCS2A01G131100 | NA |
| cSL-2A | TraesCS2A01G131200 | Stress response |
| cSL-2A | TraesCS2A01G131300 | NA |
| cSL-2A | TraesCS2A01G131400 | Signaling |
| cSL-2A | TraesCS2A01G131500 | NA |
| cSL-2A | TraesCS2A01G131600 | Development |
| cSL-2A | TraesCS2A01G131700 | protein phosphorylation |
| cSL-2A | TraesCS2A01G131800 | Translation |
| cSL-2A | TraesCS2A01G131900 | NA |
| cSL-2A | TraesCS2A01G132000 | nucleic acid binding |
| cSL-2A | TraesCS2A01G132100 | NA |
| cSL-2A | TraesCS2A01G132200 | Oxidation-reduction |
| cSL-2A | TraesCS2A01G132300 | NA |
| cSL-2A | TraesCS2A01G132400 | Transcription/Development |
| cSL-2A | TraesCS2A01G132500 | NA |
| cSL-2A | TraesCS2A01G132600 | Metabolism |
| cSL-2B | TraesCS2B01G233500 | Metabolism (lignin catabolic) |
| cSL-2B | TraesCS2B01G233600 | phosphatidylinositol phosphorylation |
| cSL-2B | TraesCS2B01G233700 | Metabolism (lignin catabolic) |
| cSL-2B | TraesCS2B01G233800 | NA |
| cSL-2B | TraesCS2B01G233900 | NA |
| cSL-2B | TraesCS2B01G234000 | Membrane |
| cSL-2B | TraesCS2B01G234100 | Development |
| cSL-2B | TraesCS2B01G234200 | nucleic acid binding |
| cSL-2B | TraesCS2B01G234300 | Transport |
| cSL-2B | TraesCS2B01G234400 | protein binding |
| cSL-2B | TraesCS2B01G234500 | Oxidation-reduction |
| cSL-2B | TraesCS2B01G234600 | Metabolism (protein) |
| cSL-2B | TraesCS2B01G234700 | Transcription/Development |
| cSL-2B | TraesCS2B01G234800 | Nucleus |
| cSL-2B | TraesCS2B01G234900 | Protein binding |
| cSL-2B | TraesCS2B01G235000 | NA |
| cSL-2D | TraesCS2D01G516100 | Mitochondrial matrix |
| cSL-2D | TraesCS2D01G516200 | Nuclease |
| cSL-2D | TraesCS2D01G516300 | Nuclease |
| cSL-2D | TraesCS2D01G516400 | NA |
| cSL-2D | TraesCS2D01G516500 | Development |
| cSL-2D | TraesCS2D01G516600 | Metabolism |
| cSL-2D | TraesCS2D01G516700 | Metabolism |
| cSL-2D | TraesCS2D01G516800 | Metabolism |
| cSL-2D | TraesCS2D01G516900 | Metabolism |
| cSL-2D | TraesCS2D01G517000 | Transcription |
| cSL-2D | TraesCS2D01G517100 | Metabolism |
| cSL-2D | TraesCS2D01G517200 | Metabolism (carbohydrate) |
| cSL-2D | TraesCS2D01G517300 | NA |
| cSL-2D | TraesCS2D01G517400 | NA |
| cSL-2D | TraesCS2D01G517500 | NA |
| cSL-2D | TraesCS2D01G517600 | Metabolism (protein) |
| cSL-2D | TraesCS2D01G517700 | NA |
| cSL-2D | TraesCS2D01G517800 | NA |
| cSL-2D | TraesCS2D01G517900 | NA |
| cSL-2D | TraesCS2D01G518000 | Protein binding |
| cSL-2D | TraesCS2D01G518100 | Transport/ Membrane |
| cSL-2D | TraesCS2D01G518200 | Metabolism (amino acid) |
| cSL-2D | TraesCS2D01G518300 | Metabolism (amino acid) |
| cSL-2D | TraesCS2D01G518400 | NA |
| cSL-2D | TraesCS2D01G518500 | NA |
| cSL-2D | TraesCS2D01G518600 | NA |
| cSL-2D | TraesCS2D01G518700 | protein binding |
| cSL-2D | TraesCS2D01G518800 | Stress response/ Transport (lipid) |
| cSL-2D | TraesCS2D01G518900 | NA |
| cSL-2D | TraesCS2D01G519000 | Transport |
| cSL-2D | TraesCS2D01G519100 | Transport |
| cSL-2D | TraesCS2D01G519200 | NA |
| cSL-2D | TraesCS2D01G519300 | NA |
| cSL-2D | TraesCS2D01G519400 | NA |
| cSL-2D | TraesCS2D01G519500 | NA |
| cSL-2D | TraesCS2D01G519600 | NA |
| cSL-2D | TraesCS2D01G519700 | NA |
| cSL-2D | TraesCS2D01G519800 | protein binding |
| cSL-2D | TraesCS2D01G519900 | Metabolism |
| cSL-2D | TraesCS2D01G520000 | Metabolism (nucleotide) |
| cSL-2D | TraesCS2D01G520100 | autophagy |
| cSL-2D | TraesCS2D01G520200 | NA |
| cSL-2D | TraesCS2D01G520300 | NA |
| cSL-2D | TraesCS2D01G520400 | NA |
| cSL-2D | TraesCS2D01G520500 | NA |
| cSL-2D | TraesCS2D01G520600 | protein binding |
| cSL-2D | TraesCS2D01G520700 | NA |
| cSL-2D | TraesCS2D01G520800 | Development |
| cSL-2D | TraesCS2D01G520900 | Transport |
| cSL-2D | TraesCS2D01G525900 | NA |
| cSL-2D | TraesCS2D01G526000 | Transcription |
| cSL-2D | TraesCS2D01G526100 | NA |
| cSL-2D | TraesCS2D01G526200 | Transcription |
| cSL-2D | TraesCS2D01G526300 | NA |
| cSL-2D | TraesCS2D01G526400 | Translation |
| cSL-2D | TraesCS2D01G526500 | NA |
| cSL-2D | TraesCS2D01G526600 | NA |
| cSL-2D | TraesCS2D01G526700 | Metabolism (P) |
| cSL-2D | TraesCS2D01G526800 | NA |
| cSL-2D | TraesCS2D01G526900 | protein binding |
| cSL-2D | TraesCS2D01G527000 | NA |
| cSL-2D | TraesCS2D01G527100 | NA |
| cSL-2D | TraesCS2D01G527200 | NA |
| cSL-2D | TraesCS2D01G527300 | Development |
| cSL-2D | TraesCS2D01G527400 | NA |
| cSL-2D | TraesCS2D01G527500 | NA |
| cSL-2D | TraesCS2D01G545100 | NA |
| cSL-2D | TraesCS2D01G545200 | NA |
| cSL-2D | TraesCS2D01G545300 | Stress response |
| cSL-2D | TraesCS2D01G545400 | Transcription |
| cSL-2D | TraesCS2D01G545500 | Oxidation-reduction |
| cSL-2D | TraesCS2D01G545600 | NA |
| cSL-2D | TraesCS2D01G545700 | Oxidation-reduction |
| cSL-2D | TraesCS2D01G545800 | Oxidation-reduction |
| cSL-2D | TraesCS2D01G545900 | Oxidation-reduction |
| cSL-2D | TraesCS2D01G546000 | NA |
| cSL-2D | TraesCS2D01G546100 | NA |
| cSL-2D | TraesCS2D01G546200 | Metabolism |
| cSL-2D | TraesCS2D01G546300 | Translation |
| cSL-2D | TraesCS2D01G546400 | NA |
| cSL-2D | TraesCS2D01G546500 | Metabolism (protein) |
| cSL-2D | TraesCS2D01G546600 | Metabolism (protein) |
| cSL-2D | TraesCS2D01G546700 | NA |
| cSL-2D | TraesCS2D01G546800 | protein phosphorylation |
| cSL-2D | TraesCS2D01G546900 | Oxidation-reduction |
| cSL-2D | TraesCS2D01G547000 | NA |
| cSL-2D | TraesCS2D01G547100 | Metabolism (protein) |
| cSL-2D | TraesCS2D01G547200 | NA |
| cSL-2D | TraesCS2D01G547300 | Metabolism (protein) |
| cSL-2D | TraesCS2D01G547400 | Oxidation-reduction |
| cSL-2D | TraesCS2D01G547500 | Oxidation-reduction |
| cSL-6A | TraesCS6A01G090600 | Metabolism |
| cSL-6A | TraesCS6A01G090700 | Oxidation-reduction |
| cSL-6A | TraesCS6A01G090800 | NA |
| cSL-6A | TraesCS6A01G090900 | Development |
| cSL-6A | TraesCS6A01G091000 | Development |
| cSL-6A | TraesCS6A01G091100 | Development |
| cSL-6A | TraesCS6A01G091200 | NA |
| cSL-6A | TraesCS6A01G091300 | NA |
| cSL-6A | TraesCS6A01G091400 | NA |
| cSL-6A | TraesCS6A01G091500 | Metabolism (protein) |
| cSL-6A | TraesCS6A01G091600 | Metabolism (protein) |
| cSL-6A | TraesCS6A01G091700 | Stress response |
| cSL-6A | TraesCS6A01G091800 | NA |
| cSL-6A | TraesCS6A01G091900 | NA |
| cSL-6A | TraesCS6A01G092000 | NA |
| cSL-6A | TraesCS6A01G092100 | NA |
| cSL-6A | TraesCS6A01G092200 | NA |
| cSL-6A | TraesCS6A01G092300 | Development (pollen) |
| cSL-6A | TraesCS6A01G092400 | NA |
| cSL-6A | TraesCS6A01G092500 | NA |
| cSL-6A | TraesCS6A01G092600 | NA |
| cSL-6A | TraesCS6A01G092700 | NA |
| cSL-6A | TraesCS6A01G092800 | NA |
| cSL-6A | TraesCS6A01G092900 | NA |
| cSL-6A | TraesCS6A01G093000 | Metabolism |
| cSL-6A | TraesCS6A01G093100 | Metabolism |
| cSL-6A | TraesCS6A01G093200 | Metabolism (carbohydrate) |
| cSL-6A | TraesCS6A01G093300 | NA |
| cSL-6A | TraesCS6A01G093400 | NA |
| cSL-6A | TraesCS6A01G093500 | NA |
| cSL-6A | TraesCS6A01G093600 | NA |
| cSL-6A | TraesCS6A01G093700 | NA |
| cSL-6A | TraesCS6A01G093800 | NA |
| cSL-6A | TraesCS6A01G093900 | NA |
| cSL-6A | TraesCS6A01G094000 | NA |
| cSL-6A | TraesCS6A01G094100 | NA |
| cSL-6A | TraesCS6A01G094200 | NA |
| cSL-6A | TraesCS6A01G094300 | NA |
| cSL-6A | TraesCS6A01G094400 | NA |
| cSL-6A | TraesCS6A01G094500 | NA |
| cSL-6A | TraesCS6A01G094600 | NA |
| cSL-6A | TraesCS6A01G094700 | NA |
| cSL-6A | TraesCS6A01G094800 | NA |
| cSL-6A | TraesCS6A01G094900 | NA |
| cSL-6A | TraesCS6A01G095000 | Metabolism (lipid) |
| cSL-6A | TraesCS6A01G095100 | Metabolism |
| cSL-6A | TraesCS6A01G095200 | NA |
| cSL-6A | TraesCS6A01G095300 | Development |
| cSL-6A | TraesCS6A01G095400 | NA |
| cSL-6A | TraesCS6A01G095500 | Metabolism (protein) |
| cKPS-2A | TraesCS2A01G439000 | NA |
| cKPS-2A | TraesCS2A01G439100 | Oxidation-reduction |
| cKPS-2A | TraesCS2A01G439200 | NA |
| cKPS-2A | TraesCS2A01G439300 | NA |
| cKPS-2A | TraesCS2A01G439400 | Oxidation-reduction |
| cKPS-2A | TraesCS2A01G439500 | Metabolism (carbohydrate) |
| cKPS-2A | TraesCS2A01G439600 | NA |
| cKPS-2A | TraesCS2A01G439700 | NA |
| cKPS-2A | TraesCS2A01G439800 | Oxidation-reduction |
| cKPS-2A | TraesCS2A01G439900 | NA |
| cKPS-2A | TraesCS2A01G440000 | protein binding |
| cKPS-2A | TraesCS2A01G440100 | NA |
| cKPS-2A | TraesCS2A01G440200 | NA |
| cKPS-2A | TraesCS2A01G440300 | NA |
| cKPS-2A | TraesCS2A01G440400 | NA |
| cKPS-2A | TraesCS2A01G440500 | NA |
| cKPS-2A | TraesCS2A01G440600 | NA |
| cKPS-2A | TraesCS2A01G440700 | NA |
| cKPS-2A | TraesCS2A01G440800 | NA |
| cKPS-2A | TraesCS2A01G440900 | Metabolism (lignin) |
| cKPS-2A | TraesCS2A01G441000 | Transcription |
| cKPS-2A | TraesCS2A01G441100 | Metabolism (lignin) |
| cKPS-2A | TraesCS2A01G441200 | Translation |
| cKPS-2A | TraesCS2A01G441300 | Transcription |
| cKPS-2A | TraesCS2A01G441400 | NA |
| cKPS-2A | TraesCS2A01G441500 | NA |
| cKPS-2A | TraesCS2A01G441600 | NA |
| cKPS-2A | TraesCS2A01G441700 | NA |
| cKPS-2A | TraesCS2A01G441800 | NA |
| cKPS-2A | TraesCS2A01G441900 | NA |
| cKPS-2A | TraesCS2A01G442000 | NA |
| cKPS-2A | TraesCS2A01G442100 | NA |
| cKPS-2A | TraesCS2A01G442200 | Metabolism |
| cKPS-2B | TraesCS2B01G589700 | Transcription |
| cKPS-2B | TraesCS2B01G589800 | Transcription |
| cKPS-2B | TraesCS2B01G589900 | NA |
| cKPS-2B | TraesCS2B01G590000 | Metabolism (lignin) |
| cKPS-2B | TraesCS2B01G590100 | NA |
| cKPS-2B | TraesCS2B01G590200 | NA |
| cKPS-2B | TraesCS2B01G590300 | NA |
| cKPS-2B | TraesCS2B01G590400 | NA |
| cKPS-2B | TraesCS2B01G590500 | NA |
| cKPS-2B | TraesCS2B01G590600 | Metabolism (protein) |
| cKPS-2B | TraesCS2B01G590700 | NA |
| cKPS-2B | TraesCS2B01G590800 | Translation |
| cKPS-2B | TraesCS2B01G590900 | Metabolism |
| cKPS-2B | TraesCS2B01G591000 | NA |
| cKPS-2B | TraesCS2B01G591100 | NA |
| cKPS-2B | TraesCS2B01G591200 | NA |
| cKPS-2B | TraesCS2B01G591300 | Metabolism |
| cKPS-2B | TraesCS2B01G591400 | NA |
| cKPS-2B | TraesCS2B01G591500 | protein binding |
| cKPS-2B | TraesCS2B01G591600 | protein binding |
| cKPS-2B | TraesCS2B01G591700 | Transcription |
| cKPS-2B | TraesCS2B01G591800 | protein binding |
| cKPS-2B | TraesCS2B01G591900 | Metabolism (carbohydrate) |
| cKPS-2B | TraesCS2B01G592000 | NA |
| cKPS-2B | TraesCS2B01G592100 | Metabolism |
| cKPS-2B | TraesCS2B01G592200 | protein binding |
| cKPS-2B | TraesCS2B01G592300 | Transcription |
| cKPS-2B | TraesCS2B01G592400 | Oxidation-reduction |
| cKPS-2B | TraesCS2B01G592500 | protein binding |
| cKPS-2B | TraesCS2B01G592600 | Transcription |
| cKPS-2B | TraesCS2B01G592700 | Transcription |
| cKPS-2B | TraesCS2B01G592800 | Transcription |
| cKPS-2B | TraesCS2B01G592900 | Transcription |
| cKPS-2B | TraesCS2B01G593000 | Oxidation-reduction |
| cKPS-2B | TraesCS2B01G593100 | Oxidation-reduction |
| cKPS-2B | TraesCS2B01G593200 | Oxidation-reduction |
| cKPS-2B | TraesCS2B01G593300 | Oxidation-reduction |
| cKPS-2B | TraesCS2B01G593400 | NA |
| cKPS-2B | TraesCS2B01G593500 | Transport |
| cKPS-2B | TraesCS2B01G593600 | Stress response |
| cKPS/SN-7B | TraesCS7B01G455000 | Metabolism |
| cKPS/SN-7B | TraesCS7B01G455100 | Metabolism |
| cKPS/SN-7B | TraesCS7B01G455200 | NA |
| cKPS/SN-7B | TraesCS7B01G455300 | Oxidation-reduction |
| cKPS/SN-7B | TraesCS7B01G455400 | Oxidation-reduction |
| cKPS/SN-7B | TraesCS7B01G455500 | Stress response |
| cKPS/SN-7B | TraesCS7B01G455600 | Metabolism |
| cKPS/SN-7B | TraesCS7B01G455700 | Metabolism (carbohydrate) |
| cKPS/SN-7B | TraesCS7B01G455800 | Metabolism (carbohydrate) |
| cKPS/SN-7B | TraesCS7B01G455900 | Metabolism (carbohydrate) |
| cKPS/SN-7B | TraesCS7B01G456000 | NA |
| cKPS/SN-7B | TraesCS7B01G456100 | Development |
| cKPS/SN-7B | TraesCS7B01G456200 | Metabolism (lignin) |
| cKPS/SN-7B | TraesCS7B01G456300 | NA |
| cKPS/SN-7B | TraesCS7B01G456400 | Metabolism (lignin) |
| cKPS/SN-7B | TraesCS7B01G456500 | Metabolism (RNA) |
| cKPS/SN-7B | TraesCS7B01G456600 | Metabolism |
| cKPS/SN-7B | TraesCS7B01G456700 | Photosynthesis |
| cKPS/SN-7B | TraesCS7B01G456800 | Metabolism (ATP) |
| cKPS/SN-7B | TraesCS7B01G456900 | Transport |
| cKPS/SN-7B | TraesCS7B01G457000 | Oxidation-reduction |
| cKPS/SN-7B | TraesCS7B01G457100 | Transport |
| cKPS/SN-7B | TraesCS7B01G457200 | Translation |
| cKPS/SN-7B | TraesCS7B01G457300 | Oxidation-reduction |
| cKPS/SN-7B | TraesCS7B01G457400 | Translation |
| cKPS/SN-7B | TraesCS7B01G457500 | Oxidation-reduction |
| cKPS/SN-7B | TraesCS7B01G457600 | Oxidation-reduction |
| cKPS/SN-7B | TraesCS7B01G457700 | Oxidation-reduction |
| cKPS/SN-7B | TraesCS7B01G457800 | Oxidation-reduction |
| cKPS/SN-7B | TraesCS7B01G457900 | Oxidation-reduction |
| cKPS/SN-7B | TraesCS7B01G458000 | Oxidation-reduction |
| cKPS/SN-7B | TraesCS7B01G458100 | NA |
| cKPS/SN-7B | TraesCS7B01G458200 | Oxidation-reduction |
| cKPS/SN-7B | TraesCS7B01G458300 | Oxidation-reduction |
| cKPS/SN-7B | TraesCS7B01G458400 | Oxidation-reduction |
| cKPS/SN-7B | TraesCS7B01G458500 | Oxidation-reduction |
| cKPS/SN-7B | TraesCS7B01G458600 | Photosynthesis |
| cKPS/SN-7B | TraesCS7B01G458700 | Stress response/ Signaling |
| cKPS/SN-7B | TraesCS7B01G458800 | Metabolism (RNA) |
| cKPS/SN-7B | TraesCS7B01G458900 | Metabolism |
| cKPS/SN-7B | TraesCS7B01G459000 | Metabolism |
| cKPS/SN-7B | TraesCS7B01G459100 | Metabolism |
| cKPS/SN-7B | TraesCS7B01G459200 | Stress response |
| cKPS/SN-7B | TraesCS7B01G459300 | Metabolism (steroid) |
| cKPS/SN-7B | TraesCS7B01G459400 | Stress response |
| cKPS/SN-7B | TraesCS7B01G459500 | protein phosphorylation |
| cKPS/SN-7B | TraesCS7B01G459600 | protein phosphorylation |
| cKPS/SN-7B | TraesCS7B01G459700 | protein phosphorylation |
| cKPS/SN-7B | TraesCS7B01G459800 | protein phosphorylation |
| cKPS/SN-7B | TraesCS7B01G459900 | protein phosphorylation |
| cKPS/SN-7B | TraesCS7B01G460000 | protein phosphorylation |
| cKPS/SN-7B | TraesCS7B01G460100 | protein phosphorylation |
| cKPS/SN-7B | TraesCS7B01G460200 | Stress response |
| cKPS/SN-7B | TraesCS7B01G460300 | Stress response |
| cKPS/SN-7B | TraesCS7B01G460400 | protein phosphorylation |

**References**

Azadi, A., Mardi, M., Hervan, E.M., Mohammadi, S.A., Moradi, F., Tabatabaee, M.T., et al. (2015). QTL Mapping of Yield and Yield Components under Normal and Salt-stress Conditions in Bread Wheat (*Triticum aestivum* L.). *Plant Molecular Biology Reporter* 33(1)**,** 102-120. doi: 10.1007/s11105-014-0726-0.

Borner, A., Schumann, E., Furste, A., Coster, H., Leithold, B., Roder, M.S., et al. (2002). Mapping of quantitative trait loci determining agronomic important characters in hexaploid wheat (*Triticum aestivum* L.). *Theoretical and Applied Genetics* 105(6-7)**,** 921-936. doi: 10.1007/s00122-002-0994-1.

Campbell, K.G., Bergman, C.J., Gualberto, D.G., Anderson, J.A., Giroux, M.J., Hareland, G., et al. (1999). Quantitative trait loci associated with kernel traits in a soft x hard wheat cross. *Crop Science* 39(4)**,** 1184-1195. doi: DOI 10.2135/cropsci1999.0011183X003900040039x.

Chu, C.G., Xu, S.S., Friesen, T.L., and Faris, J.D. (2008). Whole genome mapping in a wheat doubled haploid population using SSRs and TRAPs and the identification of QTL for agronomic traits. *Molecular Breeding* 22(2)**,** 251-266. doi: 10.1007/s11032-008-9171-9.

Cui, F., Ding, A.M., Li, J., Zhao, C.H., Wang, L., Wang, X.Q., et al. (2012). QTL detection of seven spike-related traits and their genetic correlations in wheat using two related RIL populations. *Euphytica* 186(1)**,** 177-192. doi: 10.1007/s10681-011-0550-7.

Cui, F., Li, J., Ding, A.M., Zhao, C.H., Wang, L., Wang, X.Q., et al. (2011). Conditional QTL mapping for plant height with respect to the length of the spike and internode in two mapping populations of wheat. *Theoretical and Applied Genetics* 122(8)**,** 1517-1536. doi: 10.1007/s00122-011-1551-6.

Cui, F., Zhao, C.H., Ding, A.M., Li, J., Wang, L., Li, X.F., et al. (2014). Construction of an integrative linkage map and QTL mapping of grain yield-related traits using three related wheat RIL populations. *Theoretical and Applied Genetics* 127(3)**,** 659-675. doi: 10.1007/s00122-013-2249-8.

Cuthbert, J.L., Somers, D.J., Brule-Babel, A.L., Brown, P.D., and Crow, G.H. (2008). Molecular mapping of quantitative trait loci for yield and yield components in spring wheat (*Triticum* *aestivum* L.). *Theoretical and Applied Genetics* 117(4)**,** 595-608. doi: 10.1007/s00122-008-0804-5.

Fan, X.L., Cui, F., Zhao, C.H., Zhang, W., Yang, L.J., Zhao, X.Q., et al. (2015). QTLs for flag leaf size and their influence on yield- related traits in wheat (*Triticum aestivum* L.). *Molecular Breeding* 35(1). doi: 10.1007/s11032-015-0205-9.

Ge, H.M., You, G.X., Wang, L.F., Hao, C.Y., Dong, Y.S., Li, Z.S., et al. (2012). Genome Selection Sweep and Association Analysis Shed Light on Future Breeding by Design in Wheat. *Crop Science* 52(3)**,** 1218-1228. doi: 10.2135/cropsci2010.12.0680.

Groos, C., Robert, N., Bervas, E., and Charmet, G. (2003). Genetic analysis of grain protein-content, grain yield and thousand-kernel weight in bread wheat. *Theoretical and Applied Genetics* 106(6)**,** 1032-1040. doi: 10.1007/s00122-002-1111-1.

Guo, J., Shi, W.P., Zhang, Z., Cheng, J.Y., Sun, D.Z., Yu, J., et al. (2018). Association of yield-related traits in founder genotypes and derivatives of common wheat (*Triticum aestivum* L.). *Bmc Plant Biology* 18. doi: 10.1186/s12870-018-1234-4.

Guo, Z.F., Chen, D.J., Alqudah, A.M., Roder, M.S., Ganal, M.W., and Schnurbusch, T. (2017). Genome-wide association analyses of 54 traits identified multiple loci for the determination of floret fertility in wheat. *New Phytologist* 214(1)**,** 257-270. doi: 10.1111/nph.14342.

Habash, D.Z., Bernard, S., Schondelmaier, J., Weyen, J., and Quarrie, S.A. (2007). The genetics of nitrogen use in hexaploid wheat: N utilisation, development and yield. *Theoretical and Applied Genetics* 114(3)**,** 403-419. doi: 10.1007/s00122-006-0429-5.

Hanif, M., Gao, F.M., Liu, J.D., Wen, W.E., Zhang, Y.J., Rasheed, A., et al. (2016). *TaTGW6-A1*, an ortholog of rice TGW6, is associated with grain weight and yield in bread wheat. *Molecular Breeding* 36(1). doi: ARTN 1

10.1007/s11032-015-0425-z.

Heidari, B., Sayed-Tabatabaei, B.E., Saeidi, G., Kearsey, M., and Suenaga, K. (2011). Mapping QTL for grain yield, yield components, and spike features in a doubled haploid population of bread wheat. *Genome* 54(6)**,** 517-527. doi: 10.1139/G11-017.

Huang, X.Q., Cloutier, S., Lycar, L., Radovanovic, N., Humphreys, D.G., Noll, J.S., et al. (2006). Molecular detection of QTLs for agronomic and quality traits in a doubled haploid population derived from two Canadian wheats (*Triticum aestivum* L.). *Theoretical and Applied Genetics* 113(4)**,** 753-766. doi: 10.1007/s00122-006-0346-7.

Huang, X.Q., Coster, H., Ganal, M.W., and Roder, M.S. (2003). Advanced backcross QTL analysis for the identification of quantitative trait loci alleles from wild relatives of wheat (*Triticum* *aestivum* L.). *Theoretical and Applied Genetics* 106(8)**,** 1379-1389. doi: 10.1007/s00122-002-1179-7.

Huang, X.Q., Kempf, H., Ganal, M.W., and Roder, M.S. (2004). Advanced backcross QTL analysis in progenies derived from a cross between a German elite winter wheat variety and a synthetic wheat (*Triticum aestivum* L.). *Theoretical and Applied Genetics* 109(5)**,** 933-943. doi: 10.1007/s00122-004-1708-7.

Iehisa, J.C.M., Ohno, R., Kimura, T., Enoki, H., Nishimura, S., Okamoto, Y., et al. (2014). A High-Density Genetic Map with Array-Based Markers Facilitates Structural and Quantitative Trait Locus Analyses of the Common Wheat Genome. *DNA Research* 21(5)**,** 555-567. doi: 10.1093/dnares/dsu020.

Jaiswal, V., Gahlaut, V., Meher, P.K., Mir, R.R., Jaiswal, J.P., Rao, A.R., et al. (2016). Genome Wide Single Locus Single Trait, Multi-Locus and Multi-Trait Association Mapping for Some Important Agronomic Traits in Common Wheat (*T-aestivum* L.). *Plos One* 11(7). doi: 10.1371/journal.pone.0159343.

Jia, H.Y., Wan, H.S., Yang, S.H., Zhang, Z.Z., Kong, Z.X., Xue, S.L., et al. (2013). Genetic dissection of yield-related traits in a recombinant inbred line population created using a key breeding parent in China's wheat breeding. *Theoretical and Applied Genetics* 126(8)**,** 2123-2139. doi: 10.1007/s00122-013-2123-8.

Katkout, M., Kishii, M., Kawaura, K., Mishina, K., Sakuma, S., Umeda, K., et al. (2014). QTL analysis of genetic loci affecting domestication-related spike characters in common wheat. *Genes & Genetic Systems* 89(3)**,** 121-131.

Kato, K., Miura, H., and Sawada, S. (2000). Mapping QTLs controlling grain yield and its components on chromosome 5A of wheat. *Theoretical and Applied Genetics* 101(7)**,** 1114-1121. doi: DOI 10.1007/s001220051587.

Kumar, N., Kulwal, P.L., Balyan, H.S., and Gupta, P.K. (2007). QTL mapping for yield and yield contributing traits in two mapping populations of bread wheat. *Molecular Breeding* 19(2)**,** 163-177. doi: 10.1007/s11032-006-9056-8.

Lee, H.S., Jung, J.U., Kang, C.S., Heo, H.Y., and Park, C.S. (2014). Mapping of QTL for yield and its related traits in a doubled haploid population of Korean wheat. *Plant Biotechnology Reports* 8(6)**,** 443-454. doi: 10.1007/s11816-014-0337-0.

Li, C., Bai, G., Carver, B.F., Chao, S., and Wang, Z. (2016). Mapping quantitative trait loci for plant adaptation and morphology traits in wheat using single nucleotide polymorphisms. *Euphytica* 208(2)**,** 299-312. doi: 10.1007/s10681-015-1594-x.

Li, C.L., Bai, G.H., Carver, B.F., Chao, S.M., and Wang, Z.H. (2015). Single nucleotide polymorphism markers linked to QTL for wheat yield traits. *Euphytica* 206(1)**,** 89-101. doi: 10.1007/s10681-015-1475-3.

Liu, G., Jia, L.J., Lu, L.H., Qin, D.D., Zhang, J.P., Guan, P.F., et al. (2014). Mapping QTLs of yield-related traits using RIL population derived from common wheat and Tibetan semi-wild wheat. *Theoretical and Applied Genetics* 127(11)**,** 2415-2432. doi: 10.1007/s00122-014-2387-7.

Liu, J., Feng, B., Xu, Z.B., Fan, X.L., Jiang, F., Jin, X.F., et al. (2018). A genome-wide association study of wheat yield and quality-related traits in southwest China. *Molecular Breeding* 38(1). doi: 10.1007/s11032-017-0759-9.

Liu, S.B., Zhou, R.G., Dong, Y.C., Li, P., and Jia, J.Z. (2006). Development, utilization of introgression lines using a synthetic wheat as donor. *Theoretical and Applied Genetics* 112(7)**,** 1360-1373. doi: 10.1007/s00122-006-0238-x.

Liu, Y., Lin, Y., Gao, S., Li, Z., Ma, J., Deng, M., et al. (2017). A genome-wide association study of 23 agronomic traits in Chinese wheat landraces. *Plant Journal* 91(5)**,** 861-873. doi: 10.1111/tpj.13614.

Luo, W., Ma, J., Zhou, X.H., Sun, M., Kong, X.C., Wei, Y.M., et al. (2016). Identification of Quantitative Trait Loci Controlling Agronomic Traits Indicates Breeding Potential of Tibetan Semiwild Wheat (*Triticum aestivum* ssp *tibetanum*). *Crop Science* 56(5)**,** 2410-2420. doi: 10.2135/cropsci2015.11.0700.

Ma, J., Zhang, C.Y., Yan, G.J., and Liu, C.J. (2012). Identification of QTLs Conferring Agronomic and Quality Traits in Hexaploid Wheat. *Journal of Integrative Agriculture* 11(9)**,** 1399-1408.

Ma, Z.Q., Zhao, D.M., Zhang, C.Q., Zhang, Z.Z., Xue, S.L., Lin, F., et al. (2007). Molecular genetic analysis of five spike-related traits in wheat using RIL and immortalized F-2 populations. *Molecular Genetics and Genomics* 277(1)**,** 31-42. doi: 10.1007/s00438-006-0166-0.

Mason, R.E., Hays, D.B., Mondal, S., Ibrahim, A.M.H., and Basnet, B.R. (2013). QTL for yield, yield components and canopy temperature depression in wheat under late sown field conditions. *Euphytica* 194(2)**,** 243-259. doi: 10.1007/s10681-013-0951-x.

McCartney, C.A., Somers, D.J., Humphreys, D.G., Lukow, O., Ames, N., Noll, J., et al. (2005). Mapping quantitative trait loci controlling agronomic traits in the spring wheat cross RL4452 x 'AC Domain'. *Genome* 48(5)**,** 870-883. doi: DOI 10.1139/g05-055.

Mir, R.R., Kumar, N., Jaiswal, V., Girdharwal, N., Prasad, M., Balyan, H.S., et al. (2012). Genetic dissection of grain weight in bread wheat through quantitative trait locus interval and association mapping. *Molecular Breeding* 29(4)**,** 963-972. doi: 10.1007/s11032-011-9693-4.

Mwadzingeni, L., Shimelis, H., Rees, D.J.G., and Tsilo, T.J. (2017). Genome-wide association analysis of agronomic traits in wheat under drought-stressed and non-stressed conditions. *PLoS One* 12(2). doi: 10.1371/journal.pone.0171692.

Naruoka, Y., Talbert, L.E., Lanning, S.P., Blake, N.K., Martin, J.M., and Sherman, J.D. (2011). Identification of quantitative trait loci for productive tiller number and its relationship to agronomic traits in spring wheat. *Theoretical and Applied Genetics* 123(6)**,** 1043-1053. doi: 10.1007/s00122-011-1646-0.

Neumann, K., Kobiljski, B., Dencic, S., Varshney, R.K., and Borner, A. (2011). Genome-wide association mapping: a case study in bread wheat (*Triticum aestivum* L.). *Mol Breeding* 27(1)**,** 37-58. doi: 10.1007/s11032-010-9411-7.

Nezhad, K.Z., Weber, W.E., Roder, M.S., Sharma, S., Lohwasser, U., Meyer, R.C., et al. (2012). QTL analysis for thousand-grain weight under terminal drought stress in bread wheat (*Triticum* *aestivum* L.). *Euphytica* 186(1)**,** 127-138. doi: 10.1007/s10681-011-0559-y.

Ogbonnaya, F.C., Rasheed, A., Okechukwu, E.C., Jighly, A., Makdis, F., Wuletaw, T., et al. (2017). Genome-wide association study for agronomic and physiological traits in spring wheat evaluated in a range of heat prone environments. *Theoretical and Applied Genetics* 130(9)**,** 1819-1835. doi: 10.1007/s00122-017-2927-z.

Quarrie, S.A., Steed, A., Calestani, C., Semikhodskii, A., Lebreton, C., Chinoy, C., et al. (2005). A high-density genetic map of hexaploid wheat (*Triticum aestivum* L.) from the cross Chinese Spring X SQ1 and its use to compare QTLs for grain yield across a range of environments. *Theoretical and Applied Genetics* 110(5)**,** 865-880. doi: 10.1007/s00122-004-1902-7.

Ramya, P., Chaubal, A., Kulkarni, K., Gupta, L., Kadoo, N., Dhaliwal, H.S., et al. (2010). QTL mapping of 1000-kernel weight, kernel length, and kernel width in bread wheat (*Triticum* *aestivum* L.). *Journal of Applied Genetics* 51(4)**,** 421-429. doi: Doi 10.1007/Bf03208872.

Reif, J.C., Gowda, M., Maurer, H.P., Longin, C.F.H., Korzun, V., Ebmeyer, E., et al. (2011). Association mapping for quality traits in soft winter wheat. *Theor Appl Genet* 122(5)**,** 961-970. doi: 10.1007/s00122-010-1502-7.

Shi, W.P., Hao, C.Y., Zhang, Y., Cheng, J.Y., Zhang, Z., Liu, J., et al. (2017). A Combined Association Mapping and Linkage Analysis of Kernel Number Per Spike in Common Wheat (*Triticum aestivum* L.). *Frontiers in Plant Science* 8. doi: 10.3389/fpls.2017.01412.

Suenaga, K., Khairallah, M., William, H.M., and Hoisington, D.A. (2005). A new intervarietal linkage map and its application for quantitative trait locus analysis of "gigas" features in bread wheat. *Genome* 48(1)**,** 65-75. doi: 10.1139/G04-092.

Sun, C.W., Zhang, F.Y., Yan, X.F., Zhang, X.F., Dong, Z.D., Cui, D.Q., et al. (2017). Genome-wide association study for 13 agronomic traits reveals distribution of superior alleles in bread wheat from the Yellow and Huai Valley of China. *Plant Biotechnology Journal* 15(8)**,** 953-969. doi: 10.1111/pbi.12690.

Tang, Y.L., Li, J., Wu, Y.Q., Wei, H.T., Li, C.S., Yang, W.Y., et al. (2011). Identification of QTLs for Yield-Related Traits in the Recombinant Inbred Line Population Derived from the Cross Between a Synthetic Hexaploid Wheat-Derived Variety Chuanmai 42 and a Chinese Elite Variety Chuannong 16. *Agricultural Sciences in China* 10(11)**,** 1665-1680. doi: 10.1016/S1671-2927(11)60165-X.

Wang, J.S., Liu, W.H., Wang, H., Li, L.H., Wu, J., Yang, X.M., et al. (2011). QTL mapping of yield-related traits in the wheat germplasm 3228. *Euphytica* 177(2)**,** 277-292. doi: 10.1007/s10681-010-0267-z.

Wang, R.X., Hai, L., Zhang, X.Y., You, G.X., Yan, C.S., and Xiao, S.H. (2009). QTL mapping for grain filling rate and yield-related traits in RILs of the Chinese winter wheat population Heshangmai X Yu8679. *Theoretical and Applied Genetics* 118(2)**,** 313-325. doi: 10.1007/s00122-008-0901-5.

Wu, X.S., Chang, X.P., and Jing, R.L. (2011). Genetic Analysis of Carbon Isotope Discrimination and its Relation to Yield in a Wheat Doubled Haploid Population. *Journal of Integrative Plant Biology* 53(9)**,** 719-730. doi: 10.1111/j.1744-7909.2011.01067.x.

Wu, X.S., Chang, X.P., and Jing, R.L. (2012). Genetic Insight into Yield-Associated Traits of Wheat Grown in Multiple Rain-Fed Environments. *Plos One* 7(2). doi: 10.1371/journal.pone.0031249.

Xu, X., Liu, X., Ge, S., Jensen, J.D., Hu, F.Y., Li, X., et al. (2012). Resequencing 50 accessions of cultivated and wild rice yields markers for identifying agronomically important genes. *Nature Biotechnology* 30(1)**,** 105-U157. doi: 10.1038/nbt.2050.

Xu, Y.F., Li, S.S., Li, L.H., Ma, F.F., Fu, X.Y., Shi, Z.L., et al. (2017). QTL mapping for yield and photosynthetic related traits under different water regimes in wheat. *Molecular Breeding* 37(3). doi: 10.1007/s11032-016-0583-7.

Xu, Y.F., Wang, R.F., Tong, Y.P., Zhao, H.T., Xie, Q.G., Liu, D.C., et al. (2014). Mapping QTLs for yield and nitrogen-related traits in wheat: influence of nitrogen and phosphorus fertilization on QTL expression. *Theoretical and Applied Genetics* 127(1)**,** 59-72. doi: 10.1007/s00122-013-2201-y.

Yao, J., Wang, L.X., Liu, L.H., Zhao, C.P., and Zheng, Y.L. (2009). Association mapping of agronomic traits on chromosome 2A of wheat. *Genetica* 137(1)**,** 67-75. doi: 10.1007/s10709-009-9351-5.

Yu, M., Mao, S.L., Chen, G.Y., Pu, Z.E., Wei, Y.M., and Zheng, Y.L. (2014). QTLs for uppermost internode and spike length in two wheat RIL populations and their affect upon plant height at an individual QTL level. *Euphytica* 200(1)**,** 95-108. doi: 10.1007/s10681-014-1156-7.

Yu, M., Mao, S.L., Hou, D.B., Chen, G.Y., Pu, Z.E., Li, W., et al. (2018). Analysis of contributors to grain yield in wheat at the individual quantitative trait locus level. *Plant Breeding* 137(1)**,** 35-49. doi: 10.1111/pbr.12555.

Yuan, Q.Q., Deng, Z.Y., Peng, T., and Tian, J.C. (2012). QTL-based analysis of heterosis for number of grains per spike in wheat using DH and immortalized F-2 populations. *Euphytica* 188(3)**,** 387-395. doi: 10.1007/s10681-012-0694-0.

Zhai, H.J., Feng, Z.Y., Li, J., Liu, X.Y., Xiao, S.H., Ni, Z.F., et al. (2016). QTL Analysis of Spike Morphological Traits and Plant Height in Winter Wheat (*Triticum aestivum* L.) Using a High-Density SNP and SSR-Based Linkage Map. *Frontiers in Plant Science* 7. doi: 10.3389/fpls.2016.01617.

Zhang, J.J., Dell, B., Biddulph, B., Drake-Brockman, F., Walker, E., Khan, N., et al. (2013). Wild-type alleles of *Rht-B1* and *Rht-D1* as independent determinants of thousand-grain weight and kernel number per spike in wheat. *Molecular Breeding* 32(4)**,** 771-783. doi: 10.1007/s11032-013-9905-1.

Zhang, X.Y., Deng, Z.Y., Wang, Y.R., Li, J.F., and Tian, J.C. (2014). Unconditional and conditional QTL analysis of kernel weight related traits in wheat (*Triticum aestivum* L.) in multiple genetic backgrounds. *Genetica* 142(4), 371-379. doi: 10.1007/s10709-014-9781-6
